# Grid-based methods for chemistry simulations on a quantum computer

**DOI:** 10.1126/sciadv.abo7484

**Published:** 2023-03-01

**Authors:** Hans Hon Sang Chan, Richard Meister, Tyson Jones, David P. Tew, Simon C. Benjamin

**Affiliations:** ^1^ Department of Materials, University of Oxford, Oxford OX1 3PH, UK.; ^2^ Department of Chemistry, University of Oxford, Oxford OX1 3TA, UK.; ^3^ Duality Quantum Photonics, 6 Lower Park Row, Bristol BS1 5BJ, UK.; ^4^ Quantum Motion, 9 Sterling Way, London N7 9HJ, UK.

## Abstract

First-quantized, grid-based methods for chemistry modeling are a natural and elegant fit for quantum computers. However, it is infeasible to use today’s quantum prototypes to explore the power of this approach because it requires a substantial number of near-perfect qubits. Here, we use exactly emulated quantum computers with up to 36 qubits to execute deep yet resource-frugal algorithms that model 2D and 3D atoms with single and paired particles. A range of tasks is explored, from ground state preparation and energy estimation to the dynamics of scattering and ionization; we evaluate various methods within the split-operator QFT (SO-QFT) Hamiltonian simulation paradigm, including protocols previously described in theoretical papers and our own techniques. While we identify certain restrictions and caveats, generally, the grid-based method is found to perform very well; our results are consistent with the view that first-quantized paradigms will be dominant from the early fault-tolerant quantum computing era onward.

## Teaser

Using emulated quantum computers, the impact that real quantum devices may have for chemistry dynamics simulation is studied.

## INTRODUCTION

Quantum computers may prove to be transformative tools for exploration and prediction in chemistry. When conventional computers are used for first-principles quantum molecular dynamics simulation, which is an important technique for predicting reaction outcomes and various experimental observables, the required resources (i.e., the hardware and time duration) scale exponentially with the number of simulated particles. However, these costs are expected to scale only polynomially for quantum computers, thus enabling simulations that are otherwise practically impossible. Whether and when this promise will be realized can only be predicted with a comprehensive exploration of the quantum approach. It is relevant to note that recently, a study concluded that there is as-yet no evidence of fundamental “exponential quantum advantage” in the task of computing molecular ground state energies (*1*). While that task is distinct from quantum dynamical simulation, the observation highlights a pressing need for clarity, which will doubtlessly increase as more powerful quantum computers emerge [see, e.g., (*2*)].

In this work, we investigate the prospects for accelerating chemical dynamics simulation on early fault-tolerant quantum computers using the first-quantized, real-space grid approach (*3*–*17*). By “early,” we mean machines that have a limited number of error-corrected qubits, as we presently explain. This approach involves representing wave functions over a grid of points; thus, the method explicitly encodes features such as particle symmetry (unlike the conventional second-quantized formulation). We select this approach as it is appealingly intuitive, but moreover, first-quantized simulation is anticipated by many researchers to offer the optimal resource scaling for complex and interesting molecules (*9*, *11*, *18*); some have even suggested that first-quantized simulation can efficiently encode both nuclear and electronic degrees of freedom on an equal footing, potentially addressing the gap in simulating non–Born-Oppenheimer processes in modern chemical physics (*5*).

Real-space grid methods have been used with classical computers since at least the 1980s (*19*–*23*); even if in practice, only simplified models with wave functions that are, so to speak, “heavily pixelated” can be stored and processed within conventional random-access memory. Even with quantum computers, first-quantized methods will require numerous qubits and deep circuits for meaningful realizations, making them impractical on the noise-burdened quantum computers of today. Most prior studies of such approaches have therefore focused on theoretical “pen and paper” analysis of the resource costs (*7*, *9*, *11*, *13*, *24*). In this study, we take a different approach: We deploy very substantial classical computing resources to perform exact emulations of small but noise-free quantum computers; these emulated computers then simulate representative quantum molecular dynamics. Thus, we are able to directly examine costs and performance measures.

The cost of emulation limits us to modest-sized quantum computers (we use at most 36 perfect qubits). However, we find that we can explore a number of informative scenarios within this restriction: two-dimensional (2D) and 3D simulations of one- and two-electron systems. We select specific scenarios to elucidate two key areas of interest in chemistry. We now describe these and identify small-to-medium molecules that would be important targets for early fault-tolerant quantum computers; here, we leverage our results to estimate the quantum resources required.

### Scenario I: Simulation of dynamics in the presence of strong external fields

Our exploratory work here involves a suddenly applied external field with resulting dipole oscillation and ionization of a single bound electron. Ultimately, efforts in this direction will encompass topics such as photochemistry and laser excitation. Some applications would require mature (rather than early) fault-tolerant quantum computers; for example, comprehensively modeling the dynamics of photosynthesis would be a profound accomplishment but would involve highly complex molecules, e.g., the Fenna-Matthews-Olson complex (*25*). A more near-term prospect is laser-driven dynamics: Coherent quantum control of small molecules in this way has been considered one of the “holy grails” of chemical science (*26*). A modest molecule well worthy of study would be ammonia (NH_3_), investigated in the context of selective hydrogen atom removal (*27*). If quantum modeling of its dynamics under laser control were to reveal new synthesis options, then the consequences could be profound since ammonia use lies at the heart of modern agriculture.

### Scenario II: Simulating the dynamics of particle scattering

Our exploratory work here involves an incident electron scattering from a bound electron and potentially ionizing it. In general, electron-molecule scattering is relevant in spectroscopy, astrochemistry, atmospheric chemistry, and manufacturing processes (*28*). While part of the computational challenge is scanning through possible initial energies of the incoming electron, predicting what happens upon collision and scattering is a highly quantum dynamical process that is difficult to model classically. Many cases involve reaction intermediates with fleeting lifetimes that are hard to observe and occur under conditions that are experimentally challenging to access. An example currently beyond the reach of full-dimensional quantum dynamics simulation is hexafluoro ethane (C_2_F_6_), a representative example of fluorocarbons (*29*) relevant in the chemistry of the ozone layer and in plasma etching.

Our adopted approach is to perform wave packet simulations with the split-operator quantum Fourier transform (SO-QFT) Hamiltonian simulation approach (*5*, *10*, *12*) based on the Lie-Suzuki-Trotter product formula. We model subatomic particles interacting directly via the Coulomb potential, a prerequisite for ultimately treating electrons and nuclei on a fully quantum basis. The classical SO-FT algorithm has had decades of demonstrated success in nuclear wave packet propagation (*30*) on electronic potential energy surfaces, but as far as we know, it was never used with Coulomb potentials. Compared with other first-quantized real-space Hamiltonian simulation methods [see, e.g., linear combination of unitaries (*9*) or qubitization (*11*, *13*)], SO-QFT can require the lowest number of qubits to implement time evolution (*5*).

Given that we use classically emulated quantum computers to perform grid-based simulations, the reader might wonder whether we are simply rehashing prior classical grid-based techniques under a new banner. We emphasize that this is not the case; our emulation is restricted to exactly the capabilities of real, albeit noise-free, quantum machines. This restriction is severe and manifests in multiple ways as we explore early fault-tolerant quantum computing techniques in the context of chemically relevant quantum dynamics.

Beyond the inherent value in executing previously proposed quantum algorithms and thus determining performance measures that hitherto could only be estimated, we make a number of contributions:

1) To perform scattering and ionization modeling within the finite “simulation box” of the grid-based method, we create and explore a nonunitary wave packet attenuation approach. It is inspired by complex absorbing potentials (CAPs) from classical simulation. The method uses a measurement of a single entangled ancilla qubit to attenuate particles that are ejected from the finite simulation environment, preventing them from returning at the periodic boundary and interfering with the simulation. We note that sampling the outcome of the ancilla measurement doubles up as a means of tracking escape probabilities of wave packets and has potential for quantum computing reaction rates. We present visualizations of such dynamical events that a quantum computer would enable; the user of a real quantum processor would have access to analogous images for far more complex systems.

2) Our use of the unmitigated Coulomb singularity creates a challenge in the spatial resolution, which we address by creating an augmented SO (ASO) approach. Here, we use an additional small quantum circuit to correct the Trotter error incurred at every SO-QFT time evolution step when low spatial and temporal resolution is used.

3) State preparation is a nontrivial challenge in quantum modeling. We assess prior methods and make our own contribution:

a) We investigate an approach that uses the single-ancilla iterative phase estimation (IPE) measurement to project out excited states.

b) We investigate an adaption of the single-ancilla probabilistic imaginary-time evolution (PITE) method (*12*) for approximating small imaginary-time evolution steps.

c) We build upon existing work (*31*) to create a method that explicitly generates the correct particle (anti)symmetry for first-quantized simulations.

4) Last, in light of the above studies, we estimate the quantum resource costs (time and hardware scale) for modeling the interesting molecules noted earlier, C_2_F_6_ and NH_3_. We also indicate the hardware layout of a suitable quantum computer.

The paper is structured as follows. In Results, we present a range of results from applying grid-based SO-QFT techniques to 2D and 3D systems with single and paired particles. We extrapolate from those results to estimate the quantum resources required for simulations beyond the reach of emulation, and we also present a suitable quantum hardware architecture. In Discussion, we discuss implications and remark that the SO-QFT may be advantageous in applications well beyond molecular dynamics. In Materials and Methods, we describe the methods used in SO-QFT: The “Theoretical framework” section in Materials and Methods sets out ideas described in prior works but which are provided here for a self-contained explanation with consistent notation; expert readers may care to skip directly to the “Techniques for SO-QFT modeling” section in Materials and Methods where we set out the specific methods that we use.

## RESULTS

The numerical results in this section were obtained from exactly emulated quantum processors, implemented using the open source tools QuEST (*32*), QuESTlink (*33*), and pyQuEST (*34*). Results are reported in Hartree atomic units, where the reduced Planck constant, electron mass, elementary charge, and Bohr radius are treated to be unity ℏ = *m_e_* = *e* = *a*_0_ = 1. The particular techniques used for each of the studies are specified in Materials and Methods and forward referenced from the present section. Details of important configuration choices including the alignment between the grid of pixel functions and the nuclear potential, as well as the specific hardware used, are given in the Supplementary Materials.

### Commonly used Hamiltonian and initial states

Here, we frequently use the 2D hydrogenic system described by the Hamiltonian

$${\hat{H}}_{\mathrm{tot}}=-\frac{\hbar^{2}}{2m_{e}}\nabla_{r}-\frac{e^{2}}{4\pi \epsilon_{0}|R-r|}$$ (1)

where we model the atomic nucleus as a classical discretized Coulomb potential, clamped with the origin between two pixels. Analytic solutions to Eq. 1 have been reported in (*35*, *36*). We use an equation from the former

$$\begin{array}{c} {\text{Ψ}}_{n,m}(r,\theta )=\sqrt{\frac{q_{0}^{3}(n-|m|)!}{\pi (n+|m|)!}}\times {\left(2q_{0}r\right)}^{|m|}\times e^{-q_{0}r}\\ \times L_{n-|m|}^{2|m|}(2q_{0}r)\times e^{im\theta } \end{array}$$ (2)

where $q_{0}=\frac{1}{n+1/2}$ and *L* are the generalized Laguerre polynomials. The quantum numbers *n* = 0,1,2, …, and there are (2*n* + 1) values of *m*. The energy eigenvalues are

$$E_{n}=-\frac{1}{2{\left(n+\frac{1}{2}\right)}^{2}}$$ (3)

**Fig. 1.**
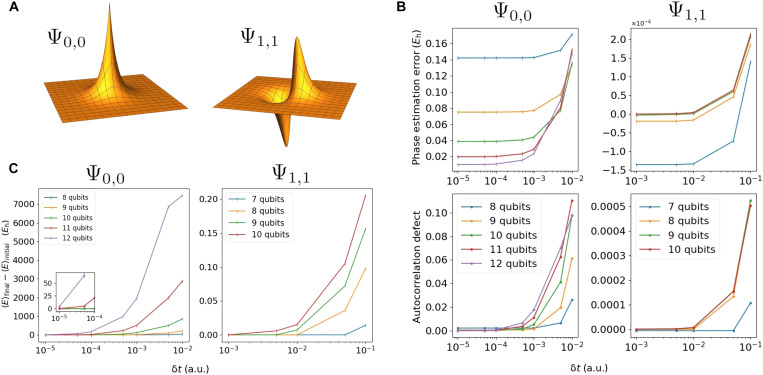
SO-QFT simulation of 2D hydrogenic electron. (**A**) Real projections of the ground ψ_0,0_ state (left) and a first excited ψ_1,1_ state (right) of 2D hydrogen. Note that the plots here do not reflect the choice of simulation box size and are not to scale. (**B**) Top represents difference between the energy from phase estimation and the analytic energy of 2D hydrogen. Bottom captures the deviation of the simulation fidelity at the end of the propagation. In this series of experiments, we initialized the ground state ψ_0,0_ centered in a simulation box with *L* = 10 a.u., such that the origin of the Coulomb singularity lies halfway between two central grid points. Each subregister has a budget of 8 ≤ *n_r_* ≤ 12 qubits to store the wave function, corresponding to spatial resolutions of 0.039 ≥ δ*r* ≥ 0.002 a.u. We also initialize the ψ_1,1_ excited state in a simulation box with sides of length 40 a.u., with budgets of 7 ≤ *n_r_* ≤ 10 qubits per subregister and corresponding resolutions of 0.313 ≥ δ*r* ≥ 0.039 a.u. These two states, represented at different spatial resolutions, are all time-propagated using the first-order SO for 1.5 atomic time units. We used time steps between 0.00001 ≤ δ*t* ≤ 0.01 (150,000 to 150 SO steps) for the ψ_0,0_ state and between 0.001 ≤ δ*t* ≤ 0.1 (1500 to 15 SO steps) for the ψ_1,1_ state. (**C**) The difference between the final and initial energy expectation value, measured by direct sampling of the state, of the ground state (left), and the first excited state (right), propagated at different spatial and time resolutions. The left inset plot zooms in on the energy error at high temporal resolutions for the ground state.

We show two of the eigenstates used in this work in Fig. 1. In a two-particle, 36-qubit simulation, we also use states related to the well-known 3D hydrogenic eigenstates

$$\begin{array}{c} {\text{Ψ}}_{n,l,m}(r,\theta ,\phi )=\sqrt{{\left(\frac{2Z}{n}\right)}^{3}\frac{(n-l-1)!}{2n(n+l)!}}\times {\left(\frac{2Zr}{n}\right)}^{l}\\ \times \;e^{-Zr/n}\times L_{n-l-1}^{2l+1}\left(\frac{2Zr}{n}\right)\\ \times \;Y_{l}^{m}(\theta ,\phi ) \end{array}$$ (4)

where $Y_{l}^{m}(\theta ,\phi )$ is a spherical harmonic and *Z* is the central nuclear charge. We also use Gaussian wave packets of the form

Ψ(x)=eIm(γ)[2Re(α)π]1/4e−α(x−xc)2+ipc(x−xc)+iγ (5)

where *x_c_*, *p*_c_, α and γ are continuous parameters.

### Spatial and temporal resolution

A key topic to explore is the number of qubits and the execution duration required to achieve simulations of a given accuracy. In the grid-based method, the model’s spatial resolution δ*r*^−1^ and the temporal resolution of the dynamics δ*t*^−1^ are crucial in determining accuracy; the qubit count per spatial dimension, *n_r_*, is logarithmically related to the former (see Eq. 27 in Materials and Methods).

To explore these requirements, we first propagate eigenstates of the 2D hydrogen with different choices of δ*r*^−1^ and δ*t*^−1^. We start with loading the discretized analytic ground and excited states Ψ_0,0_ and Ψ_1,1_ into emulated qubit registers with different numbers of qubits per spatial degree of freedom *n_r_* and then perform time evolution experiments using the first-order SO-QFT. States were propagated for 1.5 atomic time units at different time step resolutions. Figure 1 summarizes these results.

As the initial states are eigenstates, ideally, they would be static up to a global phase. Thus, the final absolute value of the autocorrelation for each propagation sequence, specifically the deviation from unity, is a suitable fidelity metric. The bottom pair of plots in Fig. 1B displays this metric for Ψ_0,0_ (left) and Ψ_1,1_ (right). When a higher spatial resolution is used, correspondingly finer time steps are needed to conserve the fidelity (see the “Coulomb potential: Demands on spatial and temporal resolution” section in Materials and Methods). This is true of both cases, but the relationship is more marked for Ψ_0,0_, as expected, given that its amplitude is peaked at the central Coulomb singularity.

Single-ancilla IPE (see the “Energy observable” section in Materials and Methods) is a simple means to extract an estimate of a system’s energy from a simulation of its dynamics on a quantum computer. Figure 1B (top) plots the deviation of this estimate from the exact analytic result. One observes qualitatively the same behavior as for the autocorrelation; there is a strong divergence when the δ*t* is insufficient and that depends on both the δ*r* and the modeled state. For the ground Ψ_0,0_ state, the error in the most accurate energy prediction (attained with the smallest time step propagation) halves when we increment *n_r_*. While deviation of the energy inferred from phase estimation versus the exact value falls exponentially with the number of qubits *n_r_*, chemical accuracy is not yet reached with *n_r_* = 12.

We note that in additional simulations, not shown in the figure, we found that by quartering the size of the simulation box and increasing *n_r_* by 1 (effectively an additional 4 qubits) and propagating at δ*t* = 10^−5^ atomic units (a.u.) for 4 a.u., we were able to achieve an error of 0.652 m*E_h_* from the estimated phase. In this case, 400,000 SO-QFT cycles were used to cover only ≈0.1 fs of physical process. While these time requirements for accurate simulation of core-peaked states like Ψ_0,0_ may seem daunting, it is worth reiterating that the approach taken here is not optimized, and we used only the first-order Trotter sequence. Moreover, as we show in the “Augmented split-operator” section in Results, an ASO approach can obtain accurate phase estimation with far fewer qubits and lower time resolution than the “brute force” method reported here. It is also noteworthy that accurate modeling of the Ψ_1,1_ state is remarkably tolerant of low resolutions.

### Cautionary tale: A “bad” energy observable

Here, we examine a sampling-based method of estimating the system’s energy that proves to be highly sensitive to inevitable imperfections in the model. Ultimately, it will converge to give the correct expected energy once spatial and temporal resolutions are sufficiently high. However, it is profoundly inaccurate at resolutions where the IPE can already provide reliable, well-converged results.

We are referring to the energy expectation as given by Eq. 37, viz. $\left\langle E\rangle =\langle {\hat{H}}_{\mathrm{kin}}\rangle +\langle {\hat{H}}_{\mathrm{pot}}\right\rangle$, and supposing that this would be obtained from our quantum computer as follows: Generate the desired state at time *t*, measure in the *k*-space representation, and repeat this many times to estimate the first term. Apply the same process but measuring in the real-space representation to estimate the second term. This method is inefficient in terms of the sampling cost and is therefore already unattractive compared to phase estimation, but more problematically, it is very sensitive to the resolution parameters. As shown in Fig. 1, the error between the initial and final expected energy grows exponentially with the size of the elementary time step δ*t*: For the ground state Ψ_0,0_, the energy difference was more than 7000 *E_h_*, accumulated over less than 40 as of simulated time in the worst offending case. In the Ψ_1,1_ simulation, the nonconservation of expected energy is also apparent when the temporal resolution does not match the spatial resolution but is more contained relative to the ground state (in the worst case, it goes up to 0.20 *E_h_*). It is evident that the core-peaked nature of the Ψ_0,0_ state is a key issue.

It is the kinetic energy term $\left\langle {\hat{H}}_{\mathrm{kin}}\right\rangle$ that exhibits this diverging behavior. The explanation is as follows: Imbalance between the extreme potential and extreme kinetic energy near the Coulomb origin, inevitable in our discrete grid representation, can allow a small amount of the amplitude to diffuse toward high-frequency states in the plane wave representation. The extent of this diffusion may be small relative to the initial state so that the fidelity of the state remains high (see Fig. 1), and thus, phase estimation methods can perform well. However, simply sampling ∣*k*⟩ and squaring it to estimate $\left\langle {\hat{H}}_{\mathrm{kin}}\right\rangle$ gives direct weight to this error that actually worsens as we improve the fineness of our resolution. Reducing δ*r*, the separation of our spatial pixels, can reduce the leakage of amplitude (increasing the state’s fidelity), but the maximum kinetic energy that the model can represent goes as 1/(δ*r*)^2^. Amplitude leakage declines less rapidly than the rate at which the energy of the leaked states increases; thus, the problem worsens. One must use extremely high time resolution to ameliorate the effect.

A higher-order Trotter formula would also presumably help, in the sense that a more modest time resolution could control the leakage. Nevertheless, we anticipate that this approach to estimating energy will always be inferior to phase estimation.

### State preparation

#### State editing

**Fig. 2.**
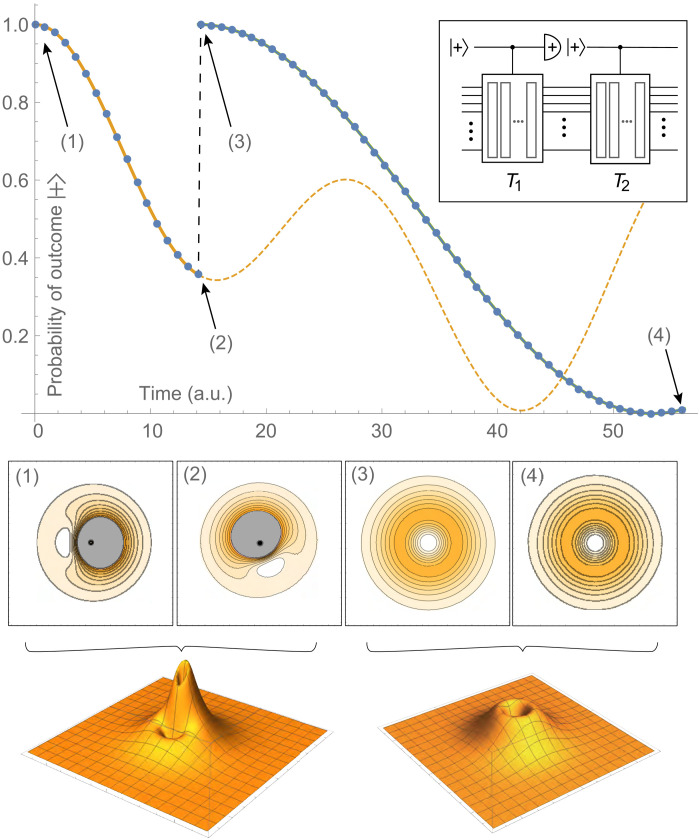
Demonstrating state editing by phase measurement. A 1 + 2 × 8–qubit quantum computer was emulated. The initial state is a superposition of nondegenerate eigenstates of 2D hydrogen. The representation uses eight qubits for each of the two dimensions, with a total simulation box width of *L* = 56 a.u. The state evolves, conditional on a controlling ancilla, for time *T*_1_ chosen such that *T*_1_*E*_1_ = π; in this period, the conditional evolution is a rotation of the state due to the accumulated phase difference between states (bottom left plots). At *T*_1_, the controlling ancilla is measured in ∣+⟩ as shown in the top right inset. This projects the state into the ψ_2,2_ state.

Earlier plots (Fig. 1) have presented the results of IPE for energy estimation. Here, we demonstrate the preparation of an initial wave packet state on a set of qubits using IPE (the “State preparation” section in Materials and Methods). Figure 2 shows the results of a simulation where the initial state [shown at the bottom left and inset (1)] is a superposition of two eigenstates of 2D hydrogen

$$\frac{1}{\sqrt{2}}({\text{Ψ}}_{1,1}+{\text{Ψ}}_{2,2})$$

The state’s amplitude is not symmetric about the nucleus, and when we apply our SO-QFT cycles, we observe a rotation of the state due to the different rates at which the two superposed eigenstates acquire phase. At the time marked (2), the total phase acquired by the more tightly bound state Ψ_1,1_ (having a more negative energy) has reached π; thus, if this state was the sole one present, a control ancilla initially in state ∣+⟩ would now certainly be in state ∣−⟩. We measure the ancilla at this point but postselect on seeing the outcome ∣+⟩. The probability of the desired outcome depends on the probability associated with the target Ψ_2,2_ within the initial superposition (which was 0.5) and the probability that this state, had it been prepared alone, would yield a ∣+⟩ outcome at this point. The latter is 0.713 in the present case. In a scenario where the states to be distinguished are closer in energy, it may be optimal to simulate for several complete cycles of the undesired state before measuring.

In our numerical emulation, we assume that the desired ∣+⟩ state is obtained, and we continue our time propagation. The new evolution of the ancilla state (green curve in the figure) is exactly that of the pure Ψ_2,2_ state. The contour plots of the simulated state [insets (3) and (4)] confirm that we have prepared that pure state. Fidelity with respect to Ψ_2,2_ was essentially identical to an initial state prepared directly in that state.

This is a demonstration of the practically useful capability to take an initial state that is not fully understood and remove from it the components corresponding to states with energies that are understood. More generally, one could use Fourier analysis (see the Supplementary Materials) to identify the components in the plot of Prob(∣+⟩) for the full state and then use the postselection method to stochastically isolate given components.

#### Probabilistic imaginary-time evolution

We now compare with an alternative approach for preparing real-space ground states on a quantum computer, which approximates imaginary-time evolution. As before, we model an attractive nucleus centered in a square simulation box. Instead of starting from an explicitly defined superposition of eigenstates as in the previous example, we initialize a Gaussian wave packet centered about the origin of the Coulomb potential. The initial Gaussian wave packet can obviously also be expanded in the eigenstate basis, which we assume has a large component corresponding to the ground state of the Hamiltonian.

**Fig. 3.**
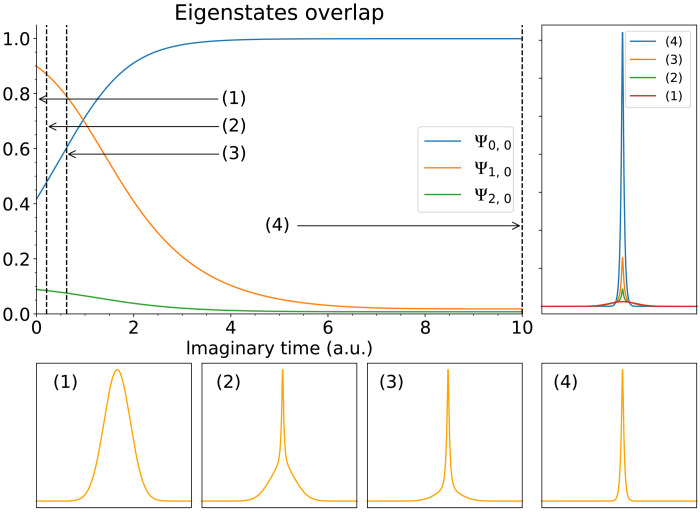
Preparing the ground state of 2D hydrogen using the PITE technique. The method was emulated on a 1 + 2 × 10–qubit quantum computer. Postselect the successful outcome at all times. The main plot shows the overlap of the propagated state with analytic eigenstates. Bottom shows scaled cross sections of the electron probability density sampled at labeled points during the imaginary-time evolution. Top right shows the same probability densities plotted on the same scale.

We then propagate the state using PITE for 300,000 steps, using *m*_0_ = 0.9 and δ*t* = 2 × 10^−5^ (note that the actual imaginary-time δτ rescales δ*t*). Figure 3 shows the state evolving under the approximate imaginary-time evolution. The main plot shows the overlap of the state with the analytic eigenstates in the “Commonly used Hamiltonian and initial states” section in Results. Only eigenstates peaked at the origin (the equivalent of *s* states in 3D) have large overlap with the state throughout the propagation, with the *n* = 1 excited state contributing more to the initial Gaussian wave packet than the ground state. In the long time limit, the ground state overlap approaches unity, whereas its overlap with higher energy states decays. The overlap signal does not go exactly to 1 and nor do the contributions of higher energy states go exactly to 0; this is because the state prepared is the ground state of the pixelated model Hamiltonian, which nonetheless has a high overlap with the true analytic ground state Ψ_0,0_ digitized to the same spatial resolution. This disparity should vanish with higher spatial resolution.

The probability distribution of the state, plotted at the bottom, also shows that the broad initial wave packet contracting to a sharp state peaked at the origin. Visually, it would appear that at a short evolved time, the state already resembles the ground state with a singular central peak. However, superimposing the sampled distributions (top right), the subsequent long time evolution appears necessary to render increased sharpness.

We further assess the state prepared from PITE by subjecting it to real-time SO-QFT propagation for 4 a.u. and estimate its energy via IPE. The fidelity does not drop below 4 × 10^−8^, and the estimated phase agrees with the converged energy at this spatial resolution reported in the previous section, further confirming that the PITE converges to the ground state of the model at this resolution.

This scenario, however, demonstrates clearly the main drawback of PITE: At every measurement, there is a substantial probability of measuring the undesired outcome; this is then a failure of the procedure. In the case described here that probability is about 0.33, this means that the cumulative success probability falls before 10^−4^ after only about 23 measurement steps. The demonstration here, with 300,000 steps, would therefore have (essentially) zero success probability on a real quantum computer. However, the method may be useful in “quantum-inspired” classical algorithms given its attractive feature of not requiring a priori knowledge of the states. Moreover, the authors of (*12*) suggest that amplitude amplification methods might address the issue of vanishing success probability.

### Quantum dynamics demonstrations

**Fig. 4.**
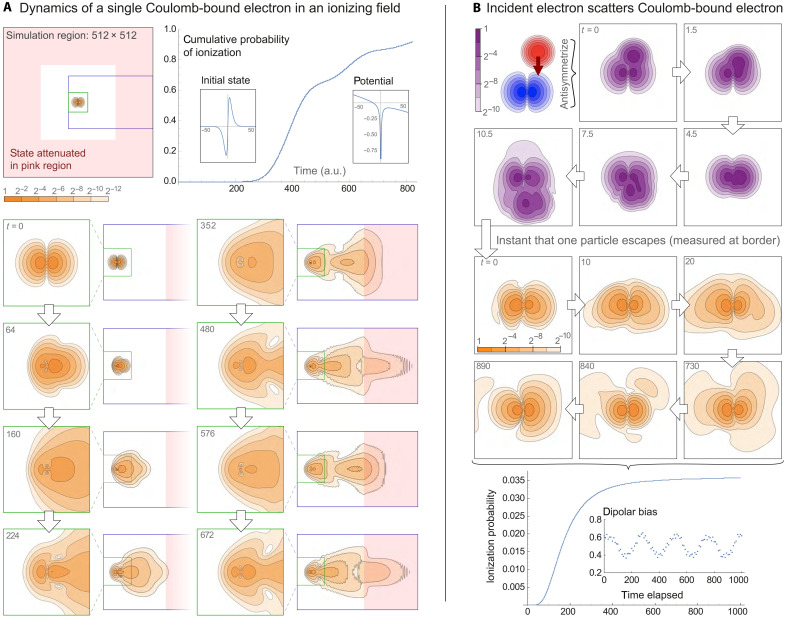
Single- and two-electron dynamics. (**A**) Figures showing a 2D simulation of a single electron ionized by a strong electric field, executed on a 1 + 2 × 9–qubit quantum computer. The initial state is a low-lying bound state of 2D hydrogen (ψ_1,1_ + ψ_1,−1_). Top left shows the initial state as a contour plot; it occupies a small region in the center of the entire simulation box (black boundary). The regions marked with green and blue borders are the zones into which we zoom in the bottom, which are a series of contour plots with the time index shown in the corner of each plot. The pink region corresponds to attenuation (a complex potential). The total probability of the particle having been found in the outer pink region is shown by the top right plot. (**B**) 2D simulation of two-particle scattering using a 25-qubit emulated quantum computer. The initial state is the antisymmetrized version of the following: an electron (blue) in a low-lying bound state of 2D hydrogen, while a second electron (red) is in a Gaussian state and moving in the negative *y* direction (downward in the plots). The simulation reveals the scattering event (purple plots) until we deem one particle to have exited. A second phase of simulation shows the evolution of the state of the remaining electron (orange), which has a probability of about 3.5% of ultimately ionizing (escaping the nuclear potential). In the event that the remaining electron does not ionize, it nevertheless remains in a state where it oscillates back and forth; the inset plot shows the quantity ⟨*x_L_*⟩> as defined in the main text.

We describe two studies that are proof-of-concept real-space grid simulations relevant to the two scenarios that we described in Introduction: ionization by strong external field and electron-electron scattering. The corresponding data are shown in Fig. 4. In both these studies, we use a method of amplitude attenuation via weak measurements, which we developed as an analog of the CAPs used in modeling with nonquantum computers. Our method allows one to track the rate at which particles exit the simulation box and that prevents them from becoming incident due to the periodic boundary conditions; it is explained in the “Attenuation and scattering” section in Materials and Methods.

#### Scenario I: Electric field ionization

In Fig. 4A, we show the performance of a 19-qubit emulated quantum computer, modeling a single 2D particle (9 + 9 qubits represent the state, and 1 qubit is used for the weak measurements). The modeled system at *t* = 0 is a state within the first excited manifold of 2D hydrogen, specifically

$$\frac{1}{\sqrt{2}}({\text{Ψ}}_{1,1}+{\text{Ψ}}_{1,-1})$$

An additional *E_x_* term in the Hamiltonian corresponds to a strong, static electric field applied in the horizontal direction; combination of the Coulomb potential and the electric field is shown in the figure inset. Because of the electric component, the initial state is no longer an eigenstate, and the simulation can determine whether the electron will be removed from the nucleus.

The initial state occupies only a small central region in the simulation box. The contour plots in the main part of the figure show the evolution, both in the center part of the simulation box (the box bordered in green) and a larger region encompassing the center and the region to the right (the box bordered in blue). The pink region, constituting the outermost 50% of the simulation box in both the *x* and *y* directions, is the region that is monitored by weak measurements.

The figure also shows the cumulative probability that the particle will have “escaped,” i.e., that it will have been measured to be in the pink region. The curve ultimately approaches unity, indicating that the particle will eventually ionize with certainty. We observe oscillations in this curve, which we can account for by examining the contour plots of particle density shown below.

Note that the contour plots show the particle’s probability density postselected on it not having yet escaped; therefore, the normalization is unity in each case. Focusing on the green boxes, those zoomed in close to the nucleus, we observe that the part of the state that remains close to the nuclear core actually oscillates in a dipole-like fashion. Note that the sequence of green panels, labeled 352 to 672, exhibits this: Panel 352 is similar to 576, while panel 480 is similar to 672. Moreover, examining the corresponding blue regions, we observe waves of density propagating away from the nucleus, synchronized with the dipole oscillation; whenever the oscillation favors the “downstream” right side, there is an enhanced probability that the particle will escape; in due course, this leads to a fluctuation in the probability of observing the particle in the pink attenuating region. It is interesting to note that this fluctuation probability is reminiscent of the bond breaking of sodium iodide observed with femtosecond pulsed lasers (*37*), an experiment recognized by the Nobel Prize in Chemistry in 1999.

On a real quantum processor, the contour plots in Fig. 4 can be obtained by repeated sampling, simply by measuring the state of the particle’s register at a given time *t*. Obtaining these outputs through brute force sampling would obviously represent a multiplicative cost depending on the accuracy with which we require the plots. The plot of the cumulative probability can also be obtained by repeatedly executing the simulation; however, it only requires measurement on a single ancilla and directly produces particle location data that can be used to determine, for example, rates in a chemical reaction dynamics simulation. We argue therefore that this approach is more useful for studying real chemistry problems on early fault-tolerant quantum computers than direct state sampling.

#### Scenario II: Two-particle scattering

Figure 4B shows the results of a two-particle scattering simulation. There are two simulation phases. In the initial phase, we have the both interacting particles present: an electron initially in a bound state of 2D hydrogen corresponding to $({\text{Ψ}}_{1,1}+{\text{Ψ}}_{1,-1})/\sqrt{2}$ and an incident electron in a Gaussian state (but with the total state properly antisymmetrized). This simulation runs until one of the particles is measured to have escaped our simulation box. We then proceed to a second phase of simulation where we study the dynamics of the surviving particle. We find that it has a small (∼3.5%) probability of ionizing due to the perturbation of the prior “impact” with the incident particle.

In the first phase, we use a 25 = 1 + 4 × 6–qubit emulated quantum processor, where the *x* and *y* coordinates of each particle are represented with 2^6^ = 64 states. As in the case of the electric field ionization described, we monitor with weak measurement a set of spatial pixels near the boundary of the simulation box. In the initial phase of this two-particle simulation, the width of that region is 25% rather than the 50% width used in the electric field case. The contour plot panels in the figure show the central nonattenuated region. The state is antisymmetrized so that the probability density plots do not distinguish one particle from the other, but informally, we can say that the incident particle interacts with the bound particle before passing on, away from the nucleus. In the case shown in the figure, we deem that a particle has been detected in the attenuation region exactly at the point when the cumulative probability of detection reaches 50% (this is an arbitrary choice; in a real quantum processor, the user would of course be unable to specify this). This event occurs shortly after the last of the panels in the top of the figure.

The simulation to that point would not teach us much about the nature of the scattering event. We could, in principle, measure, e.g., any deflection in the trajectory of the incident particle, and we can confirm (from longer simulations) that there is near-100% probability of a particle exiting the simulation region; the incident particle does not become bound. However, it is more interesting to study the subsequent behavior of the remaining particle. Because this particle is represented by a register in the quantum processor that has not “collapsed,” we can simply continue to simulate its evolution; the component *U_V_* in our SO-QFT cycle, corresponding to particle-particle interaction, will no longer be applied. We can now choose to vary other parameters such as δ*t*, anticipating that further dynamics are on a slower time scale since the high-energy particle has exited the simulation. Moreover, we can reallocate some of the qubits that were previously used to model the now-exited particle, repurposing them to model a larger simulation box. Given that the outer regions of such a box have zero amplitude associated with them at the moment that the first particle exits, there is no difficulty in simply introducing those qubits [in the case that we use the complement of the two, so that the coordinate (0,0) is in the center of the simulation box, we simply append the new qubits to the high-order end of each subregister and perform a controlled-NOT (CNOT) gate on each new qubit controlled by the prior highest-order qubit]. This was performed in the simulation shown on the right of Fig. 4, and the second phase of the simulation uses 1 + 2 × 8 qubits to provide a much larger attenuation region.

In this second phase, we observe that the remaining particle has been perturbed by the passage of the incident particle: Its distribution at *t* = 0 (now measuring time relative to the exit of the other particle) is noticeably more irregular than the simple symmetric initial form. The probability distribution is lopsided, favoring the left side. Over the remaining period of the simulation, the particle exhibits mild dipolar oscillation between a left-favored and a right-favored distribution. Defining *P*_left_(*t*) as the probability that the particle would be found left of center, we observe the oscillation shown in the inset to the time-series plot on the right of Fig. 4. Moreover, as the particle oscillates, it sheds probability, i.e., there is a finite probability that the particle will escape the simulation region. In contrast to the electric field simulation, this probability is shed symmetrically (both left and right), and it converges to a small cumulative probability of about 3.5% (see the main plot).

### Augmented split-operator

The concept of the ASO is described in the “Augmented split-operator” section in Materials and Methods. The intent is to optimize the fidelity of simulation without resorting to very high spatial or temporal resolutions, by introducing additional elements to the basic SO-QFT cycle. We assess the method by using it to simulate the dynamics of states peaked at the singularity, i.e., the most challenging cases.

**Fig. 5.**
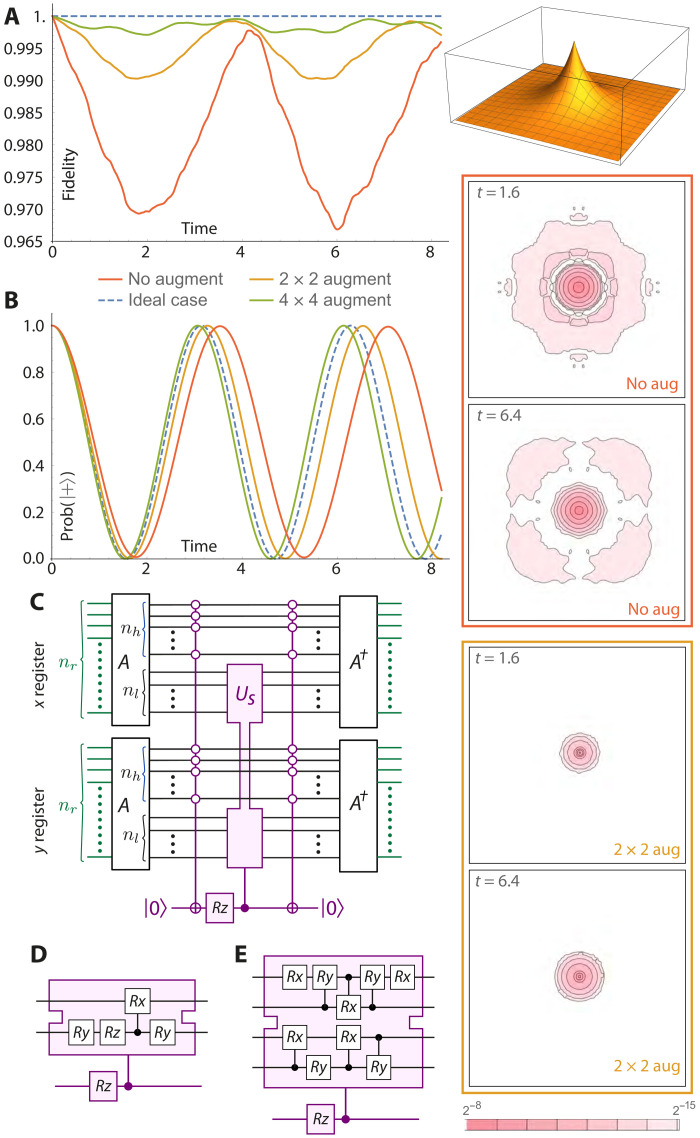
The performance of the ASO technique. The emulated quantum computer has 13 = 1 + 6 × 6 qubits. (**A**) The 3D inset depicts the eigenstate ψ_0,0_ of 2D hydrogen within its simulation box. The graph shows the autocorrelation of the state at time *t* with respect to the initial state, which ideally remains at unity (blue dashed line). The red, orange, and green lines are respectively the cases of no augmentation, a 2 × 2 augmentation, and a 4 × 4 augmentation. (**B**) The result of phase estimation for the same three cases, with the ideal again shown with a blue dashed line. The contour plots on the right show the absolute difference in probability density, with respect to the initial state, for the case of no augmentation (red) and the 2 × 2 augmentation (orange). (**C**) Generic circuit used. (**D**) and (**E**) specify the particular circuits used for our 2 × 2 and 4 × 4 augmentations, respectively. The *A* operators simply increment the indexing of the spatial “pixels” so that the bottom right pixel is (0,0). In the case of the 2 × 2 augmentation, the increment used is *G* = 1 so that *A* maps the indices of the pixels of interest, i.e., (−1,−1), (−1,0), (0,−1), and (0,0) to (0,0), (0,1), (1,0), and (1,1), respectively. These indices are now exactly those states for which the most important *n_r_* − 1 qubits are zero, facilitating the application of the small-augmentation circuit to only the four target states.

In Fig. 5, we present numerical results from our study of the ASO method. We consider the ground state of 2D hydrogen Ψ_0,0_, which is peaked (with discontinuous gradient) at the origin of the classical Coulomb potential. We use a relatively modest resolution corresponding to *n_r_* = 6 qubits per register (i.e., a 64 × 64 grid of spatial pixels) to represent the state and set the simulation box to be optimal (see the top right graphic in the figure).

Step 2 in the “Augmented split-operator” section in Materials and Methods states that, having decided that our core patch will involve a subspace of *Q* pixels, we “derive a small *Q* × *Q* unitary *U*_core_ that closely matches *U*_repair_ in that subspace.” The operator *U*_repair_ is simply the matrix that maps from the SO cycle as actually applied to the ideal time increment operator. We therefore begin by calculating these matrices, each being a 2^2*n_r_*^ × 2^2*n_r_*^ object, explicitly using Mathematica. Having thus obtained *U*_repair_, we can proceed.

In simulations reported in Fig. 5, we consider two cases: core patches of size *Q* = 2 × 2 and *Q* = 4 × 4 pixels. For each case, we write down the *Q* × *Q* matrix *M*_core_, which is composed of the elements of *U*_repair_ that lie in the *Q*-pixel subspace. *M*_core_ will not be unitary and therefore cannot be implemented deterministically on our emulated quantum computer; we need a unitary matrix *U*_core_ that is close to *M*_core_. This was obtained by performing a standard singular value decomposition of *M*_core_ and using the components to construct *U*_core_

$$M_{\mathrm{core}}=u\;\text{Σ}\;v^{†}\;\mathrm{then}\;U_{\mathrm{core}}\equiv u\;v^{†}$$

Note that Σ matrix would be the identity if *M*_core_ were unitary; it is not, but by simply omitting Σ, we generate our unitary approximation. The final step 4 of finding a circuit to implement the stabilization was performed using the circuit synthesis tool described in (*38*) for the sizes used here, which is a trivial task.

We perform a series of standard and ASO simulations, in all cases fixing the time resolution at δ*t* = 0.004, and monitor the absolute value of the autocorrelation. Figure 5A shows the result for three cases: the simple SO-QFT protocol (red) and protocols with a small (orange)– and a medium (green)–scale core stabilization augmentation. The small augmentation involves a circuit that modifies only the amplitudes associated with the 2 × 2 spatial pixels that are closest to the singularity (note that we align the spatial pixel lattice such that the singularity is midway between the four central pixels). The medium augmentation involves a larger set of 4 × 4 spatial pixels. We observe that there is a marked improvement in the autocorrelation by an order of magnitude between the red and green plots.

The plots in Fig. 5B show the result of the single-qubit phase estimation method described in the “Energy observable” section in Materials and Methods. Ideally, the autocorrelation plot would match the blue dashed curve, which corresponds to phase acquisition according to the analytically derived energy. We observe that the red, orange, and green lines are again progressively closer to the ideal. We should emphasize that the ASO method is agnostic to the state simulated; therefore, while it would be trivial to “cheat” and apply an exactly compensating phase to obtain the blue dashed curve, the circuits we have used are derived without foreknowledge of the specific simulation task (i.e., Ψ_0,0_ in no way enters the derivation of *U*_core_).

Last, a third lens on the simulation fidelity is provided by evaluating the difference between the *t* = 0 probability density (over the grid of 64^2^ spatial pixels) and the density at a later time. Ideally, of course, this difference would be zero. In the contour plots on the right of Fig. 5, we contrast the case with no augmentation with the small-augmentation scenario. While there is still a discrepancy, it is far more localized and stable (the unaugmented simulation involves wider, more marked fluctuations in the probability distribution).

The circuits that implement both the small and the medium augmentations are shown on the bottom left of Fig. 5. They were derived through the process explained in the “Augmented split-operator” section in Materials and Methods. Note the preparatory step of applying a unitary *A* that simply adds an integer to all states in the spatial representation, i.e.

A∣n⟩=∣n+G⟩

where the addition is understood to be modulo 2*^n_r_^*. The main figure shows the implementation of this for a general “size” of the augmentation patch *n_l_*, where in our numerical studies *n_l_* = 1 for the small case and *n_l_* = 2 for the medium case. The figure caption defines the shift for the case of the 2 × 2 augmentation, where we will use *G* = 1. The *A* operators can be avoided entirely if we simply define the origin of our spatial coordinates to a bounding corner of our core stabilization region. The implementation of the spatial part of the SO would need to be corrected for such shift but that may prove to have lower total cost. Regardless, we include the *A* operators in the figure so as to make it directly consistent with the other circuits and expressions in the present paper.

We note that the multicontrolled-NOT operation appearing in the circuits of Fig. 5 can be compactly realized by recently discovered circuits (*39*) that involve 4*n* − 6 T-gates (single-qubit phase gates) and a comparable number of control-NOTs, together with an ancilla that is measured during the process. We anticipate that the time cost of moving from the simple SO-QFT approach to the ASO method should be far less than the cost of the brute force increase to the temporal resolution needed to assure proper behavior of core-peaked states (see the “Coulomb potential: Demands on spatial and temporal resolution” section in Materials and Methods). For the present demonstration, the performance of the *n_r_* = 6 qubit registers was able to approximately match that of the *n_r_* = 11 qubit registers under a brute force approach (Fig. 1, top left), and moreover this, was achieved with a time step δ*t* that is an order of magnitude greater, so facilitating more rapid runtimes. The ASO method is therefore highly relevant when one operates with the strict Coulomb interaction, as in all the numerical studies in the present paper. It remains to be seen whether it would also be useful in scenarios where the Coulomb singularity is approximated by some of the other means listed in the “Coulomb potential: Demands on spatial and temporal resolution” section in Materials and Methods. The ASO method is distinct from, and compatible with, the use of higher-order Trotter sequences. Throughout the paper, we restrict to the lowest-order Trotter pattern, but extending this is an interesting direction for further work.

### 3D helium simulation

Last, we extend the low-dimensional demonstrations to simulating the dynamics of a helium atom: two electrons interacting via a repulsive Coulomb interaction, both bound by a central attractive Coulomb potential, in three spatial dimensions. As the true electron eigenstates of the helium atom cannot be solved exactly, we approximate the two-electron initial state by combining two single-electron solutions of the 3D hydrogen-like Schrödinger equation (Eq. 4), with a central nuclear charge of *Z* = 2. Note that this would be an exact eigenstate if there were no electron-electron interactions. The two sets of quantum numbers (*n*, *l*, *m*) that we used were (2,1,0) and (2,1, −1), of which the former is the 2*p_z_* orbital and the latter is the atomic orbital $2p_{-1}=(2p_{x}-i\;2p_{y})/\sqrt{2}$. The complete initial (triplet) state is then the antisymmetric wave function

$${\text{Ψ}}_{\mathrm{init}}({\vec{r}}_{1},{\vec{r}}_{2})={\text{Ψ}}_{2,1,0}({\vec{r}}_{1})\;{\text{Ψ}}_{2,1,-1}({\vec{r}}_{2})-{\text{Ψ}}_{2,1,-1}({\vec{r}}_{1})\;{\text{Ψ}}_{2,1,0}({\vec{r}}_{2})$$ (6)

As our simulation box, we use a cube with side lengths of 25 a.u. and discretize the initial function Ψ_init_ as per Eq. 30 using *n_r_* = 6 qubits per register, providing 64 divisions per axis and per particle. We then propagate the full 36-qubit state forward in time for 500 steps, where each step is of length 0.05 a.u., thus having a total evolution of 25 a.u. It is worth noting that this relatively simple initial state has symmetries that could be exploited for a more compact representation; however, we wished to test the full 3D two-particle grid representation, and therefore, we did not exploit any such properties.

**Fig. 6.**
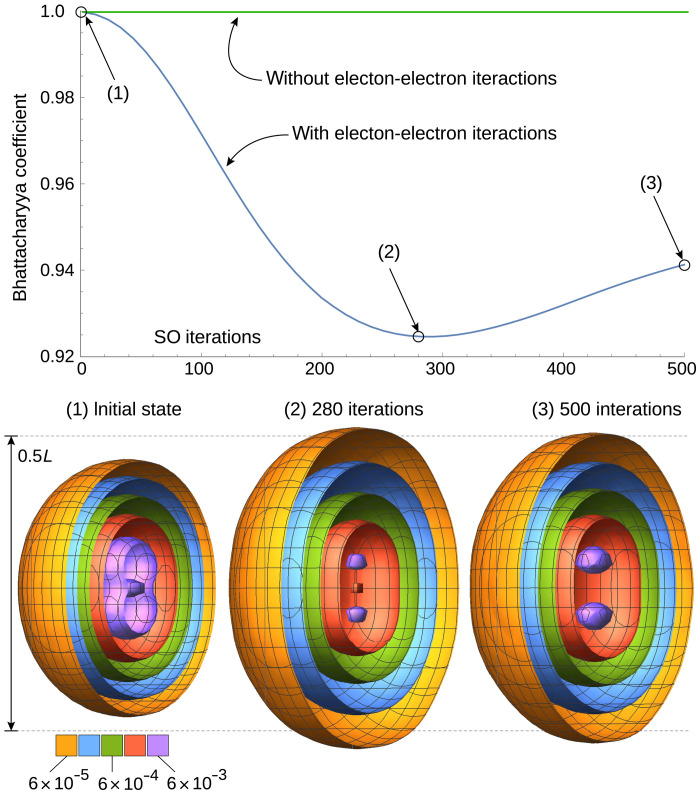
Simulation of a helium atom in real space. The top plot is the Bhattacharyya coefficient (with and without enabled electron-electron interactions), and the bottom is the real-space electron density distributions during the time evolution of the helium atom simulation. Colored shells are electron probability density isosurfaces, within a simulation box with *L* = 25 a.u. Distributions are shown for the initial state, at the time where it is maximally spread out, and at the end of the simulation. The 500 SO cycles correspond to the propagation of 25 a.u. (≈0.6 fs).

The single-ancilla phase estimation method was used in previous sections (see, e.g., Fig. 2) to track the evolution of the simulated system’s state. However, this requires our emulator to use double the memory that would be needed simply to represent and propagate the state. Since the resource costs for the present emulation are already considerable, we opted instead to compute and record, at every SO-QFT time step, the probability density of one of the electrons (since the two electrons are indistinguishable, the probability density is equal). Specifically, we record the probability associated with each computational basis state of the three registers corresponding to one of the particles. Using these probability distributions, we can compute the Bhattacharyya coefficient (*40*) of the distribution at time *t* with respect to the distribution of the initial state Ψ_init_. This quantity is

$$\sum_{i}\sqrt{p_{i}}\sqrt{q_{i}}$$

for our two discrete probability distributions *p* and *q*. It can be thought of as a classical analog of the usual inner product fidelity. It is plotted in Fig. 6.

As the initial state is not an eigenstate, we expect the distribution to vary over time. As shown in Fig. 6, the electron density is initially distributed with rotational symmetry around the vertical *z* axis, with charge accumulations in the positive and negative *z* directions. Because the electrons partly shield each other from the core, the chosen central charge of the initial state *Z* = 2 is too large, and thus, the electron orbitals are too close to the core. The time evolution shows that the charge initially spreads out away from the core in every direction and then returns slightly but not to its original distribution. To confirm that the interaction between the electrons is the cause for this behavior, we also performed an identical calculation but with the *e*-*e* interaction disabled. Figure 6 shows that, in this case, the probability distribution simply stays constant. We thus confirm that our simulation directly shows the effect of electron shielding in this hypothetical configuration.

One could repeat the experiment to find the value for the effective nuclear charge that allows our analytic initial state to most closely approximate a true helium eigenstate; moreover, one could use the methods of the “State preparation” section in Materials and Methods to actually prepare the eigenstate from such an initial approximation. These are interesting tasks for further study.

### Antisymmetrization of the initial state

In the “State preparation” section in Materials and Methods, we discuss the preparation of an initial state of our grid-based simulator with proper antisymmetrization of the electrons. We assume that there is some set of *P* single-particle basis states ψ*_i_*, each of which we know how to prepare on a register (possibly via a repeat-until-success probabilistic method). We note that it would be convenient to simply prepare a product state over our *P* particle registers and subsequently antisymmetrize it.

We observe that one means of doing so involves first finding a Hamiltonian *H*_synth_ with the property that

$$H_{\mathrm{synth}}|\psi_{i}\rangle =E_{i}|\psi_{i}\rangle ,\;E_{i+1}>E_{i}$$ (7)

Here, *H*_synth_ need not correspond to any physically legitimate scenario. One means of obtaining *H*_synth_ would be to start from a description of the chemical system of interest and then introduce modifications to conveniently localize Hamiltonian terms and to break any degeneracies in the single-particle solutions. In this way, the available basis states ∣ψ*_i_*⟩ will be close to the canonical choice of basis states that might be made in, e.g., chemistry modeling with a conventional computer. While this is an interesting topic to consider further, we will not do so in the present paper, but rather, we will simply assume that *H*_synth_ can be found.

Given that *H*_synth_ is synthetic, it can of course be scaled and shifted arbitrarily. We will align the energies conveniently with respect to the binary states that can be represented by a register of *t* bits. For example, we can set *E*_0_ = 0 and *E*_*P*−1_ = 2*^t^* − 1 where *t* is sufficiently large that the smallest energy gap min (*E*_*i*+1_ − *E_i_*) is at least unity.

We can proceed in at least two distinct ways, and we will describe the first in detail. It requires a modest number of additional qubits: For each of the *P* particle registers, introduce a “tag” ancilla register of *t* qubits.

1) Prepare the particle registers as ∣ψ_0_⟩∣ψ_1_⟩…∣ψ_*P*−1_⟩.

2) Set the *i*th tag register to the integer closest to *E_i_*, which we write as $E_{i}^{\prime }$.

3) Permute the entire object (the particle registers and their tag registers) into antisymmetric form by any means that can permute an initially ordered list of integers, for example, the inverse sorting network method of (*41*). We simply apply this method to the tags while “dragging along” the particle registers “for the ride.” The result is the state

$$\frac{1}{\sqrt{P!}}\sum_{i}^{P!}{\mathrm{perm}}_{i}[(|E_{0}^{\prime }\rangle |\psi_{0}\rangle )\ldots (|E_{P-1}^{\prime }\rangle |\psi_{P-1}\rangle )]$$ (8)

with the notation (∣tag⟩∣particle state⟩).

4) Erase the tag registers through phase estimation: Apply a QFT to every tag register and then apply operations of the form controlled exp (*iH*_sym_*C*π) from each tag register qubit to its main register, where *C* is an appropriate power of two.

5) Measure out the tag register qubits in the *x* basis; with high probability, all should be in the ∣+⟩ state, and this outcome is our success criterion.

If all energies *E_i_* can be exactly represented with *t* bits, then $E_{i}^{\prime }=E_{i}$ for all *i* values and the method will succeed with certainty. Moreover, even in the in the event that the energies cannot be perfectly represented in this binary form (as will be the case, for example, if two energies differ by an irrational number), the only consequence is that the success criterion in step 5 will have a reduced probability of occurring. Given that it does occur, the final state remains ideal.

We explored this using classical emulation, on inputs of up to *P* = 5 particles. In this case, 30 qubits were used: *t* = 3 tag qubits for each particle and a further 3 qubits to represent each corresponding ψ state. We considered several choices of spectrum *E_i_* and proceeded in each case as follows. We prepared the state given in Eq. 8 directly in the emulator’s random-access memory: It is readily verifiable by inspection that the first three steps lead to this state regardless of the choice of *E_i_*, whereas the additional qubits required to permute via the sorting network would have greatly increased the emulation cost. We then performed the final steps explicitly and noted the performance.

For *P* = 5 particles, with the ideal case that all energies correspond to integers, we confirmed the expected success probability of unity. For cases where the all energies differed from an integer, we found success probabilities of 0.990, 0.960, and 0.850 for deviations of 0.025, 0.05, and 0.1, respectively, showing an anticipated quadratic behavior. However, when only one of the energies differs from an integer by some specified discrepancy, then we find that the success probability is essentially constant regardless of *P*. Thus, the performance for a large number of particles will depend on how many of the energies differ from an integer and to what extent (with respect to 2^−*t*^). We note that in each emulated scenario, we confirmed that given the “all-∣+⟩” success criterion, the resulting state is ideal (up to a meaningless global phase). In the Supplementary Materials, we outline the alternative method, which is similar but allows one to create and destroy the tag register on the fly, thus reducing the qubit count while increasing the computation time.

### Quantum computer resources and architectures

In the following two subsections, we assess on the resource requirements for undertaking modeling beyond the reach of classical algorithms and the related question of suitable quantum architectures.

#### Resources for postclassical modeling

In this section, we reflect upon the implications of our numerical results for the resource demands of postclassical chemistry modeling. We will not undertake a formal resource scaling analysis, noting instead that asymptotic analyses have been made in the past few years for real-space grid, first-quantized Hamiltonian simulation (*7*, *9*, *11*) and specifically the SO-QFT method (*13*). Complementing these analyses, our present work implemented grid-based simulations using emulated quantum computers that have proven large enough to elucidate practical issues, such as the number of basis functions required for given levels of accuracy in specific observables. Data of this kind will be of use in estimating the constants that must appear in any resource scaling analysis.

The lowest possible qubit count (for a given simulation accuracy) results from selecting the smallest adequate simulation box length *L* and setting the spatial resolution δ*r* (Eq. 27) to be just sufficient to capture the most curved elements of the wave function (see the “Coulomb potential: Demands on spatial and temporal resolution” section in Materials and Methods). The number of qubits per particle, per spatial dimension, is then *n*_*r*_ = log_2_ (*L*/δ*r*). The quantity *L* simply specifies the region of space outside of which our multiparticle wave function only has negligible amplitude “clipped” by the boundary. Moreover, as we explain in the “Attenuation and scattering” section in Materials and Methods, we can study processes that would, over the simulation time, go beyond the simulation box due to scattering or ionization. It is reasonable to assume, as in (*12*), for example, that the volume *L*^3^ goes linearly with the number of particles *P*; a molecule with twice the number of particles requires (of order) twice the volume. Meanwhile, the severity of the wave function’s curvature should scale directly with *Z*_max_, the maximum nuclear charge of any of the nuclei in the system (the “Coulomb potential: Demands on spatial and temporal resolution” section in Materials and Methods). Thus, increasing the molecule’s size without increasing *Z*_max_ should not require any adjustment in δ*r*, but the simulation box may have to be larger to accommodate more particles. The accuracy with which we model the particle is essentially unaffected by this change.

In view of the above observations, we can expect that for suitable constants *C_i_*

$$\begin{array}{rl} n_{r}=\log_{2}\left(\frac{L}{\delta r}\right) & \approx \log_{2}\left(\frac{C_{1}P^{\frac{1}{3}}}{C_{2}Z_{\max}^{-1}}\right)\\ & =C_{3}+\log_{2}(Z_{\max})+\frac{1}{3}\log_{2}(P) \end{array}$$ (9)

The total qubit count will be 3*Pn_r_* for *P* particles in 3D. Note that this simple expression for *n_r_* does not account for the potentially helpful fact that atomic radii have a highly nonlinear (and sublinear) dependence on the number of electrons (*42*). In the Supplementary Materials, we make an estimate of the root term *C*_3_ from our numerical results; we argue that *C*_3_ ∼ 10 is optimistic but not unreasonable.

Using some further assumptions, we can make an estimate of the number of qubits necessary to model the important scenarios that we described in Introduction: electron scattering of hexafluoro ethane (C_2_F_6_) and quantum coherent control of the ammonia molecule (NH_3_). In the Supplementary Materials, we suggest that grid-based modeling of C_2_F_6_ may require about 2250 computational qubits, even with frugal use of ancilla qubits. Fortunately, our estimate for the coherent control of NH_3_, being one of smallest relevant cases, is far lower at 450 qubits (with relatively optimistic assumptions).

The overall time cost for simulation depends of course on the hardware realization but is certainly interesting to discuss. As the methods we propose will almost certainly require a fault-tolerant quantum computer, the most relevant metric for time complexity on such a machine may be the T-gate count. This is a measure of how many steps in the algorithm correspond to the costly non-Clifford operations that cannot be directly performed in stabilizer codes, and efforts to minimize the count for standard subroutines are ongoing (*43*). For example, a recent note has reduced the number of such gates needed for multicontrolled rotations to a remarkably frugal level (*39*); such rotations are key in both our attenuation and ASO techniques. More generally, the trade-off between time and qubit count is a research topic in its own right, and recent papers have shown how markedly this balance can be adjusted (*44*, *45*).

The overall “wall clock” duration of a simulation is determined by the number of T-gates needed for each complete SO-QFT cycle, the duration each of these steps represents (the time resolution δ*t*), and the total duration of the dynamical process under investigation. In the Supplementary Materials, we use various observations and assumptions to estimate the gate depth for simulating interesting chemical physics, noting that dynamical processes where quantum effects are meaningful can occur from subfemtosecond to picosecond time scales. For simulations of rapid events such as ionization, we estimate an algorithmic gate depth of *O*(10^8^), while for a more challenging simulation of physics over longer time scales, we suggest that a depth of *O*(10^11^) may be required.

Translating gate count to execution time will vary markedly depending on the native physical gate error rate (typically assumed to be 10^−3^ or 10^−4^), the speed of a stabilizer cycle, and the option to trade computation speed for higher qubit overheads (*45*). For surface code–based implementations with solid-state platforms, a credible stabilizer speed (*46*) is 1 μs (with faster speeds being conceivable). Assuming that the state preparation procedure only requires a polynomial-scaling overhead and is thus not the dominant cost, this gives a clock time of the order of minutes for the more simple simulations, while the challenging long–time scale process would require a clock time on the order of a day.

We must also note that generating certain interesting plots (equivalent to those shown here) will require many repeated executions of the simulation. Fortunately, such repetitions can be perfectly parallelized over independent quantum processors, which need not have quantum interlinks or even be colocated.

The same full-dimensional real-space grid simulation of the reaction on classical machines of course is not possible, simply because the sheer number of pixels in the simulation is beyond the memory available on most high-performance computer clusters. An equivalent classical procedure of selecting reduced reaction coordinates where the dynamics is expected to be relevant, computing the corresponding electronic potential energy surfaces, and subsequent dynamics propagation can require months of effort; finalizing the electronic structure itself can often be the main bottleneck. We therefore conclude that the quantum Hamiltonian simulation approach presented can be more efficient than an equivalent classical method, based on the fact that a more complete picture is used and the effort of computing potential energy surfaces is circumvented.

#### Quantum computer architectures

The preceding section obtained back-of-the-envelope estimates of qubit counts ranging from several hundreds to several thousands. While this sounds encouraging, we note the very likely need for fault tolerance and the resulting multiplicative increase in physical qubit count. Presently, the most well-understood codes can require many hundreds of physical qubits per logical qubit, assuming relatively deep algorithms and physical error rates comparable to today’s best quantum computer prototypes. Even if one makes the very optimistic assumption that some form of error mitigation can suffice in place of full fault tolerance, at least for small molecular simulations, the more powerful forms of mitigation can require a multiplicative increase in the number of physical qubits (*47*, *48*). Thus, the number of physical qubits required for the modeling considered in the preceding section could easily reach the high thousands or millions. This raises the question of what kind of architecture would be needed.

In particular, we are motivated to explore whether some form of network architecture might be compatible with the SO (or ASO) approach. Such a network might involve quantum computers interlinked within a building, analogously to a conventional High Performance Computing facility, and relevant methods of linking processors have been experimentally realized. Alternatively, for suitably compact platforms, the network might correspond to linked quantum computing processors on a single chip, analogous to today’s multicore central processing units; multicore quantum computing has recently been explored (*49*). In either case, it is realistic to assume that in a network of processor nodes, the internode operations are slower than the intranode ones.

Fortunately, the SO-QFT method is quite compatible with a network paradigm; there is a natural partitioning of the problem into nodes that each contain the registers (or subregisters) associated with a given simulated particle. While data exchange between nodes is obviously required, it need not be a dominant component of the overall resource costing even if the physical links are slow.

**Fig. 7.**
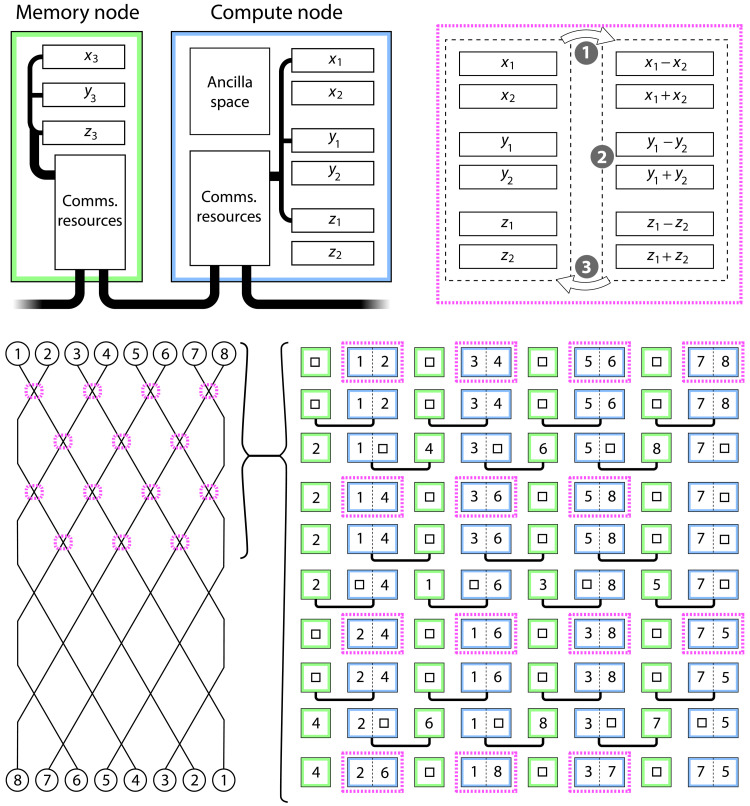
A possible “multicore” architecture. On the basis of distributing the *P* particle state over *P*/2 memory nodes (3*n_r_* qubits) and *P*/2 compute nodes (6*n_r_* qubits), which are interlinked either on-chip (*49*) or at the macroscale. Top right indicates that three steps occur in a compute node. In step 1, quantum addition and subtraction are used to move to the relative and total coordinates (and step 3 will reverse this). In the middle, step 2, the relative coordinate is used to apply phase shifts required by the SO cycle, and optionally, we apply an augmentation step. Bottom shows how parallelized phases alternatingly process and permute the data. The numbers within the circles and squares are the labels of particles; they correspond to the subscripts of the *x*, *y*, and *z* symbols on the top right.

In Fig. 7, we show one possible partitioning: It is not the most granular, since one could assign individual subregisters to cores, but it does strike a good balance between the intra- and intercore operations. Note that the required connectivity between cores is merely linear and nearest-neighbor. We suppose that there are two forms of core: The compute nodes are responsible for all the processing that is associated with the SO-QFT method, while the simpler memory nodes only store registers transiently.

Each node includes communication resources that facilitate transfer of quantum information between nodes, for example, through the use of teleportation enabled by high-quality shared Bell pairs. The “comms resources” would thus correspond to Bell pair distribution, purification, and buffering. Such processes can occur independently of the main computation and simultaneously with it and need not involve a large number of qubits; see, for example, the analysis in (*49*, *50*).

In the scheme illustrated in Fig. 7, the transfer of a register from one node to another involves writing into an “empty” register where all qubits are in state ∣0⟩. If the individual qubits are encoded logical states formed of many physical qubits, then this would introduce a multiplicative factor in the Bell pair count, but it would remain linear in the register size. Ideally, the “comms” hardware would be capable of generating the required Bell pairs within the time that the compute node requires for a full implementation of the particle-particle interaction component for the current pair (and any augmentation, as per the “Augmented split-operator” section in Materials and Methods). Given that this computation will require a gate depth of at least $n_{r}^{2}$, there is scope for a factor of 10 to 100 in the relative speeds of the intracore computations and the intercode Bell preparation before the latter would become a bottleneck.

## DISCUSSION

In this work, we explore the SO-QFT approach on exactly emulated qubits and test resource-frugal techniques that facilitate augmentation and monitoring of first quantized, real-space quantum chemistry simulations. We test known quantum techniques and others that we introduce, covering all key aspects of quantum simulation: state preparation, Hamiltonian simulation, and the extraction of physical observables. Thus, we characterize the resources needed to realize a “digital experiment” of quantum molecular dynamics (*18*) on early fault-tolerant quantum computers.

The methodologies that we presented can become part of a learning/prediction cycle that augments physical experiments, providing accurate datasets for machine-learned emulators that can accelerate chemical discovery. We believe that the SO-QFT method, in tandem with the resource-frugal approaches presented here, may prove itself superior to classical quantum molecular dynamics simulations relatively early in the era of fault-tolerant machines. We have already noted that the technique itself leads to robust methods for measuring observables such as phase estimation. We have also noted that we are free to check certain properties quite cheaply at any time, analogous to stabilizers of a code, and their cost of evaluation can be low even if preparing the state was not. In the PITE and attenuation cases, we have also used frequent measurement to modify the evolution of the system in a nonunitary manner. In light of this, it is interesting to ask whether the grid method can be inherently robust to errors. It may be possible to craft a version of the algorithm where most harmful errors will cause the state to fail a validation check, while less damaging errors are mitigated by a nonunitary component in the dynamics.

While combining SO-QFT with other Hamiltonian simulation methods may lead to hybrid quantum-classical approaches for real-space simulations, the high qubit count for encoding the first-quantized grid representation and generally deep circuits will likely prevent its applicability in the Noisy Intermediate Scale Quantum (NISQ) era. Nevertheless, in the early fault-tolerant regime, small executions of these methods might offer synergies with real-space electronic structure approaches such as density functional theory (DFT); one can imagine using small exact calculations enabled by real-space quantum simulations to improve DFT functionals or using particle densities provided by an initial DFT precomputation to inform the single-particle functions that are loaded into particle registers.

A natural next step is to explore the use of multiresolution grids as used in, e.g., MADNESS (*23*, *51*), the highly successful classical computing program for real-space grid simulations. We are well motivated to incorporate such ideas given that the present paper has revealed remarkable variation in resolution requirements, even across different states of a single system. Moreover, particles within many-body systems can often be considered as localized, presenting the opportunity for frugal representations based on that locality. However, while multiresolution grids might reduce the qubit count considerably, it would likely be at the cost of more sophisticated time propagators and basis transformations. Thus, it is important to establish whether the methods in, e.g., (*23*, *51*), can be translated successfully to the quantum context.

It is obvious that the SO-QFT simulation techniques presented here can be generalized to modeling systems beyond quantum chemistry. The solution to any Cauchy-type initial value problem

$$\frac{\partial }{\partial t}\text{Ψ}(x,t)=\hat{D}(t)\text{Ψ}(x,t)$$ (10)

with a time-dependent differential operator that may be separated into operators that are respectively diagonal in position and momentum space

$$\hat{D}(t)={\hat{D}}_{1}(t)+{\hat{D}}_{2}(t)$$ (11)

can be approximated with the SO-QFT approach (*52*). Many problems of interest can be modeled with Cauchy-type partial differential equations. For example, Dirac and Klein-Gordon equations, which are of Cauchy form, reconcile quantum mechanics with special relativity and may be used for modeling of high-energy particles (*53*). A very different application is financial engineering. Quantum advantage is often promised for the modeling of how the prices of assets, such as options and derivatives, evolve over time (*54*, *55*). This is key to executing purchases and sales that maximize the eventual payoff from trading such assets. These assets often have complex underlying dependence on random variables, which have, in practice, been modeled using computationally expensive stochastic Monte Carlo methods. In the same manner as pixelating real-space wave functions and storing them in the computational basis states of a quantum computer, probability distributions corresponding to asset prices can be discretized and loaded into quantum registers. A very relevant model for time propagation of these distributions is the Black-Scholes-Merton equation (*56*, *57*), which is a Cauchy-type partial differential equation. Beyond these use cases, it remains to be seen how nonunitary operations achieved through ancilla measurements can extend the applications of the SO-QFT model.

## MATERIALS AND METHODS

This section is divided into two parts. The first introduces the theoretical framework, i.e., the essential physics and notation, as well as the core concepts for the grid paradigm. The second describes the specific methods explored and evaluated in Results, including techniques developed for this study.

### Theoretical framework

The nonrelativistic time evolution of a quantum state is governed by the time-dependent Schrödinger equation

$$\frac{\partial }{\partial t}\text{Ψ}(r,s,t)=-i\hat{H}\text{Ψ}(r,s,t)$$ (12)

where Ψ is the normalized, complex-valued, many-body wave function defined by the spatial **r** and spin **s** coordinates of the constituent particles (note that throughout this work, we are using atomic units where ℏ = 1). For the systems of interest here, the Hamiltonian $\hat{H}$ is

$${\hat{H}}_{\mathrm{tot}}={\hat{H}}_{\mathrm{kin}}+{\hat{H}}_{\mathrm{int}}$$ (13)

where

$${\hat{H}}_{\mathrm{kin}}=-\sum_{p=1}^{P}\frac{1}{2m_{p}}\nabla_{p}^{2}$$ (14)

represents the kinetic energy of each of the *P* particles present, and ${\hat{H}}_{\mathrm{int}}$ encompasses all interactions. In many cases, it is convenient to further resolve according to

$${\hat{H}}_{\mathrm{int}}={\hat{H}}_{U}+{\hat{H}}_{V}$$ (15)

where ${\hat{H}}_{U}$ represents single-particle interactions with, e.g., classical fields, while ${\hat{H}}_{V}$ represents particle-particle interactions. For interacting charged particles (electron-electron, nucleus-electron, and nucleus-nucleus), we would write

$${\hat{H}}_{V}=\sum_{p,q=1;p\neq q}^{P}\frac{q_{p,q}}{|r_{p}-r_{q}|}$$ (16)

for suitable constants *q*. In the case of atomic and molecular systems, we could opt to model each nucleus as a full quantum particle in its own right, in which case, we may set ${\hat{H}}_{U}=0$ unless there are external, e.g., electric or magnetic fields. In their seminal paper, Kassal *et al.* (*5*) argue that this is the natural choice given the relatively modest additional resources needed.

If the nuclei are not treated explicitly within the model, we then opt to model only the electrons and use classical fields to represent the *M* nuclear potentials originating at fixed locations **R***_m_* according to

$$\begin{array}{rl} {\hat{H}}_{U} & =\sum_{m=1}^{M}\;\sum_{p=1}^{P}\frac{Z_{p,m}}{|r_{p}-R_{m}|}\\ & =\sum_{p=1}^{P}\frac{Z_{p}}{|r_{p}|}\;\mathrm{for}\;M=1\;\text{nucleus at}\;R_{0}=0 \end{array}$$ (17)

for suitable values of nuclear charges *Z_p_* or *Z*_*p*,*m*_. In the 2D and 3D atomic simulations we report, we consider *M* = 1 nucleus as in the equation above. However, the techniques generalize naturally to *M* > 1. External and possibly time-dependent potentials can also be included in the Hamiltonian. Presently, we will consider the case of uniform electric field **E**, by including within ${\hat{H}}_{U}$ a term

$${\hat{H}}_{E}=\sum_{p=1}^{P}Q_{p}r_{p}\;\cdot \;E$$

Formally, the solution to Eq. 12 is

$$\text{Ψ}(r,s,t)=e^{-i\hat{H}t}\text{Ψ}(r,s,0)$$ (18)

For the multielectron Hamiltonian with more than two particles, it is not possible to analytically evaluate the action of the time evolution operator $e^{-i\hat{H}t}$, and one has to resort to numerical techniques to solve the initial value problem. A practical time propagation method therefore involves selecting a representation of the state and then applying (some approximation of) the time evolution operator.

We begin by providing a brief summary of the real- and momentum-space (*k*-space) grid representations of a many-body wave function, suitably encoded on a quantum computer. We then discuss the SO-QFT method for simulating Hamiltonian dynamics. We refer the reader to the Supplementary Materials for a detailed presentation of these topics. We highlight that this method of exploiting quantum computers, explored by earlier authors in (*5*–*8*, *10*–*12*, *16*, *17*), is an adaption of the classical computing methods developed in (*30*, *58*–*60*), which we also review in the Supplementary Materials.

#### Representations in real and momentum space

Consider a system of *P* quantum particles in *d* spatial dimensions, well localized within a region of volume *L^d^* (has negligible amplitudes beyond throughout the simulation), which we refer to as the simulation box. In this work, we use the approach where this system is represented on a quantum computer by partitioning the qubits into *P* registers, and each register is further divided into *d* spatial subregisters each with *n_r_* qubits. Each particle is thus discretized into an evenly spaced grid with 2*^dn_r_^* basis functions, either in a spectal, finite basis representation (FBR) or its dual pseudo-spectral basis, also called the discrete variable representation (DVR). The coefficients of the wave function expansion in this grid representation map directly onto the amplitudes of the computational basis (*3*–*5*). The number of qubits in the register therefore scales linearly as *O*(*dPn_r_*) and logarithmically with the number of grid basis functions. The favorable asymptotic scaling is one of the main advantages of first-quantized real-space grid–based encoding; in the second-quantized representation, the required number of qubits scales linearly with the number of basis functions (sites or orbitals) (*5*, *11*).

We choose an FBR where the plane wave basis state of the modeled system is represented by a state of the computer’s subregister as follows

$$\phi_{k}(x)=L^{-\frac{1}{2}}\;\exp\left(\frac{i2\pi kx}{L}\right)↔|k\rangle$$ (19)

Here, *k* is an integer, and ∣*k*⟩ refers to the computational basis state which, regarded as a binary string, corresponds to *k*. Defining ρ = 2^*n_r_*−1^ and noting that we have 2ρ basis states in our computer’s subregister, a natural choice for the allowed *k* is to run from −ρ through zero to ρ − 1. Therefore, a 1D single-particle state Ψ would be represented by our subregister ∣ψ*_x_*⟩ according to

$$\text{Ψ}=L^{-\frac{1}{2}}\sum_{k=-\rho }^{\rho -1}a_{k}e^{i2\pi kx/L}↔|\psi_{x}^{\mathrm{KS}}\rangle =\sum_{k=-\rho }^{\rho -1}a_{k}|k\rangle$$ (20)

The superscript KS denotes the *k*-space representation.

We generate the dual DVR on a quantum computer by applying, to each subregister, a QFT denoted by *U*_QFT_ (see the Supplementary Materials for its quantum circuit). The subregister as a whole will be transformed as

$${|\psi \rangle }_{x}^{\mathrm{RS}}=U_{\mathrm{QFT}}{|\psi \rangle }_{x}^{\mathrm{KS}}=\sum_{n=-\rho }^{\rho -1}b_{n}|n\rangle$$ (21)

where

$$b_{n}=\frac{1}{\sqrt{2\rho }}\sum_{k=-\rho }^{\rho -1}\exp\left(i\frac{n\pi }{\rho }k\right)a_{k}$$ (22)

The superscript RS indicates the real-space representation. When we wish to return to the original *k*-space representation, we use the inverse QFT

$${|\psi \rangle }_{x}^{\mathrm{KS}}=U_{\mathrm{QFT}}^{†}{|\psi \rangle }_{x}^{\mathrm{RS}}=\sum_{k=-\rho }^{\rho -1}a_{k}|k\rangle$$ (23)

where of course

$$a_{k}=\frac{1}{\sqrt{2\rho }}\sum_{n=-\rho }^{\rho -1}\exp\left(-i\frac{k\pi }{\rho }n\right)\;b_{n}$$ (24)

**Fig. 8.**
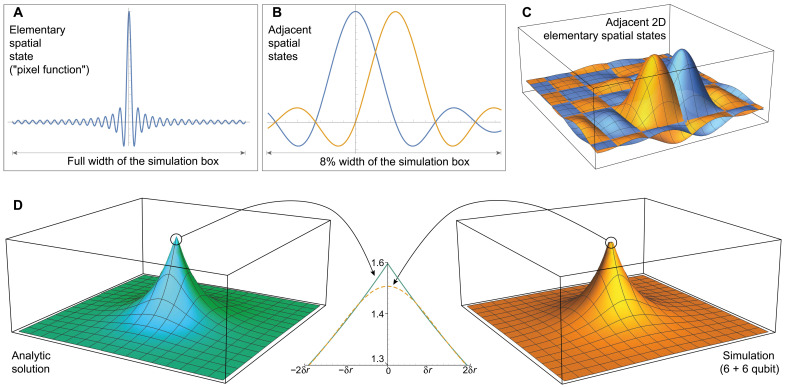
Basis of spatial DVR wave functions modeled by a quantum computer. (**A**) A pixel function $P_{n_{r}}^{n}(x)$ with *n_r_* = 6 qubits and *n* = 0 (neglecting its complex phase; see Eq. 27). (**B**) Adjacent pixel functions *n* = 0 and *n* = 1; where a function has its primary peak, all others are zero. (**C**) 2D pixel functions formed from products of the 1D cases. (**D**) Representing a continuous state in this DVR. Left (blue green) shows the exact ground state ψ_0,0_ of 2D hydrogen. Right (orange) is the same state approximately modeled by a 6 + 6 = 12 qubit quantum computer. The fidelity of the modeled state with respect to the analytic state (integrating over all space) is 0.99946. If instead we project the analytic state into the box and renormalize, then the infidelity between the model and the analytic solution falls below 10^−4^. The discrepancy is a ≈6% variation localized at the Coulomb singularity, where the analytic expression has a gradient discontinuity, as highlighted by the central plot. The sharpness of the approximation is characterized by δ*r*^−1^ and so increases exponentially with *n_r_*.

We find that the basis functions represented by each computational basis ∣*n*⟩ appearing in Eq. 21 are peaked at (but not strictly localized around) the spatial point $x_{n}=\frac{n}{\rho }\frac{L}{2}$. The top left of Fig. 8 shows a plot for the case that *n_r_* = 6, *n* = 0. Specifically, the inferred mapping is

$$\phi_{n}(x)=P_{n_{r}}^{n}(x)↔|n\rangle$$ (25)

with

$$P_{n_{r}}^{n}(x)=\exp\left(-\frac{i\pi x^{\prime }}{L}\right)\sqrt{\frac{2}{\rho L}}\;\sum_{j=1}^{\rho }\cos\frac{\pi (2j-1)x^{\prime }}{L}$$ (26)

where *x*′ = *x* − *x_n_* with $x_{n}=\frac{nL}{2\rho }$, and ρ = 2*^n_r_^* − 1. The function $P_{n_{r}}^{n}(x)$, which we informally refer to as a “pixel function,” serves the role of an approximation or “smear” of the Dirac delta function Δ(*x* − *x_n_*), the sharpness increasing as 2*^n_r_^* tends to infinity. The separation between the peaks of adjacent pixel functions, e.g., *x*_2_ − *x*_1_, is δ*r* = *L*/2*^n_r_^*, and we now define the model’s spatial resolution as the reciprocal of this quantity, i.e., as the number of pixel functions per unit distance

$$\delta r^{-1}=2^{n_{r}}/L$$ (27)

A suitable decomposition to represent any 1D wave function Ψ(*x*) in our quantum register is therefore very intuitive

$$\text{Ψ}(x)\approx \frac{C}{\sqrt{\delta r}}\sum_{n=-\rho }^{\rho -1}\text{Ψ}(x_{n})P_{n_{r}}^{n}(x)↔\frac{C}{\sqrt{\delta r}}\sum_{n=-\rho }^{\rho -1}\text{Ψ}(x_{n})|n\rangle$$ (28)

i.e., the required amplitude of ∣*n*⟩, the state representing the wave function peaked at point *x_n_*, is found simply by sampling the target wave function at that point. Here, *C* is a normalization constant that will be close to unity providing that (i) the target wave function has negligible amplitude outside of the simulation box and (ii) the target wave function varies slowly with respect to δ*r*. Intuitively, one can think of these spatial basis functions $P_{n_{r}}^{n}(x)$ analogously to pixels as used in conventional digital photographs: The greater the number of spatial pixels or grid points, the more features of the wave function are adequately captured.

The duality between the momentum- and real-space representations under the *U*_QFT_ has been explored in multiple grid-based quantum simulation studies, including (*5*, *7*, *10*, *12*). Whereas the *k*-space representation maps qubit basis states to wave functions with sharp values of *k*, in the dual representation, the mapping is to wave functions that are not perfectly sharp around points in real space. Nonetheless, because of their Dirac-like nature, one can analyze techniques and protocols as if they were Dirac functions, secure in the knowledge that, in the high-resolution limit, this becomes exact. This gives the visually intuitive picture that a particle is a (pixelated) wave function in real space, supported by a basis of sharp, evenly spaced functions; this insight is used in most prior works (*5*, *10*, *12*, *13*). The appealing conceptual simplicity over second quantization can be regarded as another merit of first-quantized grid representation.

The generalization to 2D or 3D is the natural one: The subregisters tensor together to form the complete representation of a given particle. The 3D analog of Eq. 28 is

$$\begin{array}{rl} \text{Ψ}(r) & \approx C\delta r^{-\frac{3}{2}}\sum_{n,m,l=-\rho }^{\rho -1}\text{Ψ}(x_{n},y_{m},z_{l})P_{n_{r}}^{n}(x)P_{n_{r}}^{m}(y)P_{n_{r}}^{l}(z)\\ & ↔C\delta r^{-\frac{3}{2}}\sum_{n,m,l=-\rho }^{\rho -1}\text{Ψ}(x_{n},y_{m},z_{l})|n\rangle |m\rangle |l\rangle \end{array}$$ (29)

When we generalize to represent a *P* particle wave function Ψ(**r**_1_, …, **r***_P_*), we need only to extend in the natural fashion

$$C\delta r^{-\frac{3P}{2}}\sum_{\{n_{1}..l_{P}\}=-\rho }^{\rho -1}\text{Ψ}(x_{n_{1}},y_{m_{1}},\ldots ,z_{l_{P}})|n_{1}\rangle |m_{1}\rangle \ldots |l_{P}\rangle$$ (30)

#### Split-operator propagation

The SO-QFT exploits these representation choices, and the low computational cost incurred to transform between them, to approximate the time evolution operator *e*^−*iH*δ*t*^. Evolution by a total time *t* is discretized into short time intervals δ*t* such that

$$e^{-i\hat{H}t}=\underset{N\;\mathrm{times}}{\underbrace{e^{-i\hat{H}\delta t}e^{-i\hat{H}\delta t}\ldots e^{-i\hat{H}\delta t}}}=U^{N}(\delta t)$$ (31)

and the SO-QFT approximates the unitary short-time propagator using Lie-Trotter-Suzuki product formula (or Trotterization), splitting the Hamiltonian into its kinetic and interacting parts

$$\begin{array}{rl} e^{-i\hat{H}\delta t} & =e^{-i({\hat{H}}_{\mathrm{kin}}+{\hat{H}}_{\mathrm{int}})\delta t}\\ & =e^{-i{\hat{H}}_{\mathrm{kin}}\delta t}e^{-i{\hat{H}}_{\mathrm{int}}\delta t}+O(\delta t^{2})\\ & =U_{\mathrm{SO}}(\delta t)+O(\delta t^{2}) \end{array}$$

**Fig. 9.**
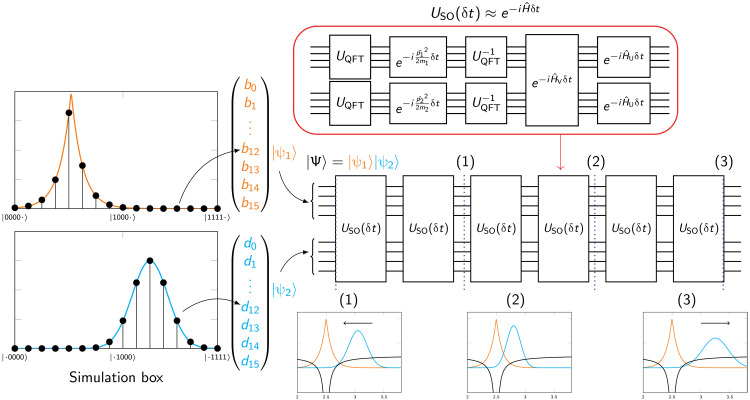
Real-space grid Hamiltonian simulation using the SO-QFT approach. In this example, the dynamics of two electrons in a simulation box is simulated: One is modeled as a Gaussian wave packet, and the other is modeled as the bound ground-state hydrogen solution. The two interact via a repulsive Coulomb potential, and the atomic nucleus is modeled as a classical attractive Coulomb potential. The particles are digitized into regularly spaced pixels, encoded into the state of two qubit subregisters of the same size. The starting state is a tensor of these two states (a Hartree product), and upon antisymmetrization of the two particles (which would generate a Slater determinant), the initial state can then be propagated in time by repeated application of the SO-QFT small-time evolution operator *U*_SO_(δ*t*) (depicted by the top circuit). A possible sequence of the subsequent dynamics, where the unbound electron scatters with the bound electron, is plotted at the bottom.

Higher-order splitting schemes and their numerical properties are well documented (*61*–*65*). For simplicity and to compare different techniques on an equal basis, in this work, we focus on the first-order SO-QFT, summarized in Fig. 9. The methods that we use are equally compatible with any Trotter sequence; although where $[{\hat{H}}_{\mathrm{kin}},{\hat{H}}_{\mathrm{int}}]\neq 0$, the dynamics will be imperfectly modeled and gives rise to the Trotter error terms as in Eq. 32; we discuss further in the “Coulomb potential: Demands on spatial and temporal resolution” section in Materials and Methods.

The real- and momentum-space grid representations (detailed in the “Representations in real and momentum space” section in Materials and Methods) are natural options for state representation when computing the approximate time evolution operator of Eq. 32: In the *k*-space representation, the kinetic part of the Hamiltonian ${\hat{H}}_{\mathrm{kin}}$ is separable and exactly local (diagonal); in the real-space representation, the interaction part of the Hamiltonian ${\hat{H}}_{\mathrm{int}}$ is approximately diagonal and would be exact if the basis of pixel functions were Dirac delta functions. Because the gate complexity for the *U*_QFT_ only scales quadratically with the number of qubits per particle subregister *n_r_* (see the Supplementary Materials for the QFT circuit), the SO-QFT can very efficiently compute the two phases of the short-time propagator by periodically transforming each subregister independently into their preferred, diagonal basis

$${|\psi (t+\delta t)\rangle }^{\mathrm{RS}}=U_{\mathrm{SO}}(\delta t)\;{|\psi (t)\rangle }^{\mathrm{RS}}$$

where

$$U_{\mathrm{SO}}(\delta t)=e^{-iD_{\mathrm{int}}\delta t}(U_{\mathrm{QFT}}^{†}\;e^{-iD_{\mathrm{kin}}\delta t}\;U_{\mathrm{QFT}})$$ (32)

Here, *D*_kin_ and *D*_int_ are diagonal real matrices, and *U*_QFT_ is the QFT applied to all subregisters.

We discuss in detail the evaluation of these operators on quantum computers in the Supplementary Materials, but we provide a summary here. From Eq. 14, we observe that propagation under the kinetic Hamiltonian ${\hat{H}}_{\mathrm{kin}}$ separates exactly into a product of operators acting independently on each particle and in each spatial subregister because the components commute

$$U_{\mathrm{kin}}(\delta t)=\prod_{p=1}^{P}\prod_{q\in \{x,y,z\}}\exp\left(-i\frac{\delta t}{2m_{p}}k_{q}^{2}\right)$$

where *k* again refers to the momentum state *k* from Eq. 19. The required quadratic phases are introduced onto our computational basis states in the momentum-space representation according to

$$|k\rangle \Rightarrow e^{-iC\delta t\;k^{2}}|k\rangle$$ (33)

and can be achieved using a sequence of single- and two-qubit phase gates [see, for example, (*7*, *10*)], which scales as $O(n_{r}^{2})$.

Propagation under the interaction potentials ${\hat{H}}_{U}$ and ${\hat{H}}_{V}$ is only modestly more complex than the former kinetic propagation. For interaction of particles with classical fields ${\hat{H}}_{U}$, we are primarily interested in an attractive Coulomb potential representing a nucleus (although we discuss a variation including a static electric field presently). For a single nucleus with charge *Z*, we write a time evolution operator

$$U_{U}(\delta t)=\prod_{p=1}^{P}\exp\left(-i\frac{Z\delta t}{\sqrt{x^{2}+y^{2}+z^{2}}}\right)$$

The operations are independent between the registers corresponding to different particles but not independent between subregisters assigned to a given particle. The phases changes that we apply to our quantum registers are

$$\begin{array}{rl} |n\rangle |m\rangle |l\rangle & \Rightarrow \exp\left(\frac{-iZ\;\delta t}{\sqrt{x_{n}^{2}+y_{m}^{2}+z_{l}^{2}}}\right)|n\rangle |m\rangle |l\rangle \\ & =\exp\left(\frac{-iZ\;\delta t}{\delta r\sqrt{n^{2}+m^{2}+l^{2}}}\right)|n\rangle |m\rangle |l\rangle \end{array}$$ (34)

Efficient evaluation of functions such as the inverse square root on quantum computers is an active area of development; we highlight (*5*, *7*, *14*, *66*) in these directions. Here, it suffices to note that the number of gates required can scale quadratically with the number of qubits *n_r_* (*7*, *66*), and we discuss this further in the Supplementary Materials.

For the interaction between quantum particles ${\hat{H}}_{V}$, the propagation is of the form

$$U_{V}(\delta t)=\exp\left(-i\delta t\sum_{p,q=1;p\neq q}^{P}\frac{q_{p,q}}{|r_{p}-r_{q}|}\right)$$ (35)

To compute this propagation, we consider two particle registers, each composed of three subregisters, which represent a given pair of particles *p* = 1 and *p* = 2. The basis states will be updated as

$$|n_{1}\;m_{1}\;l_{1}\rangle |n_{2}\;m_{2}\;l_{2}\rangle \Rightarrow e^{-i\text{Θ}}n|n_{1}\;m_{1}\;l_{1}\rangle |n_{2}\;m_{2}\;l_{2}\rangle$$

where

$$\begin{array}{rl} \text{Θ} & =\frac{Z_{p,q}\;\delta t}{\sqrt{{\left(x_{n_{1}}-x_{n_{2}}\right)}^{2}+{\left(y_{m_{1}}-y_{m_{2}}\right)}^{2}+{\left(z_{l_{1}}-z_{l_{2}}\right)}^{2}}}\\ & =\frac{Z_{p,q}\;\delta t}{\delta r\sqrt{{\left(n_{1}-n_{2}\right)}^{2}+{\left(m_{1}-m_{2}\right)}^{2}+{\left(l_{1}-l_{2}\right)}^{2}}} \end{array}$$ (36)

We discuss how this is computed in the “Quantum computer architectures” section in Results when we consider appropriate computer architectures and in further detail in the Supplementary Materials.

We again emphasize that, when propagating *H*_U_ and *H*_V_ in the real-space representation, the operators are approximated to be diagonal. This would be exact in the limit of infinite spatial resolution, and the spatial states were Dirac delta functions. Under this approximation, the two terms commute, and we expect that for a sufficiently small δ*t* and a smoothly varying potential, an adequate spatial resolution δ*r*^−1^ will result in dynamics that converge to the exact behavior. However, the Coulomb potential is singular at **r** = 0, and consequently, we will need to investigate the behavior in this region carefully.

### Techniques for SO-QFT modeling

We now present techniques and important considerations specific to SO-QFT modeling. We outline the methods known in the literature but tested here in a quantum chemistry setting while highlighting those methods that (to our knowledge) have not been previously described.

#### Energy observable

In this first section, we review energy measurement for grid-based quantum simulations; the experienced reader may care to skip to the “State preparation” section in Materials and Methods.

##### Energy expectation

The energy expectation is the most ubiquitous observable in quantum chemistry. In the grid-based approach, it is possible to split the Hamiltonian and calculate the kinetic energy expectation in *k* space, followed by the potential energy expectation in real space

$$\left\langle E\rangle =\langle \text{Ψ}|U_{\mathrm{QFT}}^{†}{\hat{H}}_{\mathrm{kin}}U_{\mathrm{QFT}}|\text{Ψ}\rangle +\langle \text{Ψ}|{\hat{H}}_{\mathrm{int}}|\text{Ψ}\right\rangle$$ (37)

where, here, ∣Ψ⟩ is understood to be the spatial representation. Aside from requiring inefficient repeated sampling of the quantum state, as we demonstrate in the “Cautionary tale: A “bad” energy observable” section in Results, the aforementioned Trotter error gives rise to nonconservation of this energy expectation and renders the method unsuitable for extracting the correct physics from simulation.

##### Iterative phase estimation

The energy of a state can also be extracted by tracking the global phase that it acquires during time propagation. This is given by the phase of the autocorrelation function, which is a discrete time series of the inner product between a propagated state and the initial state. Suppose that we time propagate an eigenstate Ψ*_n_* of ${\hat{H}}_{\mathrm{tot}}$ with energy *E_n_*. The corresponding autocorrelation signal is

$$\langle {\text{Ψ}}_{n}(t)|{\text{Ψ}}_{n}(t=0)\rangle =e^{-iE_{n}t}$$ (38)

where the absolute value ∣⟨Ψ(*t* = 0)∣Ψ(*t*)⟩∣ should be close to unity, and in this work, we use it as one measure of a simulation’s veracity.

On a quantum computer, this otherwise-unobservable global phase is efficiently extracted using phase estimation. Phase estimation through the use of ancilla qubits (*67*) is one of the fundamental techniques used in diverse applications of quantum computing, and its utility in the context of SO-QFT and first-quantized simulation is well recognized [see, e.g., (*5*)].

**Fig. 10.**
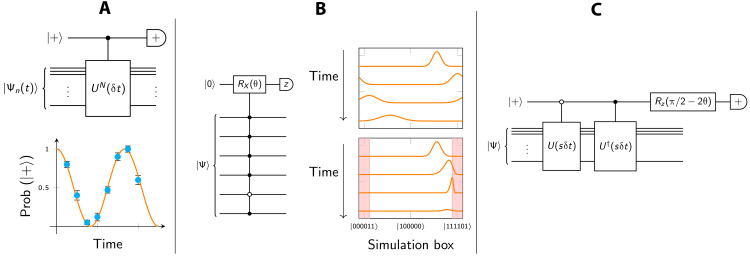
Three early fault-tolerant quantum circuit techniques for real-space chemistry explored in this work. (**A**) The single-ancilla IPE method emulated in this work. The global phase is encoded in the probability of measuring an entangled ancilla qubit, which controls the application of *N* SO cycles, in the ∣+⟩ state. To obtain this probability, one must repeat the propagation and measurement at each point in time where one wishes to sample the signal. (**B**) Attenuation of a wave packet on a qubit register. Top right depicts the dispersion of a Gaussian wave packet across the periodic boundary. Addition of complex absorbing region in pink (bottom right) attenuates the scattered wave packet by reducing its norm. In the illustrated case, the process is not quite perfect: There is some reflection caused by the attenuation being too severe. Left is a circuit that performs probabilistic wave packet attenuation at a select pixel. The pixel ∣111101⟩ is selected, corresponding to the attenuating region on the right-hand side of the simulation box in the figure. (**C**) Preparing ground states using the PITE circuit. The filled circles indicate “control by ∣1⟩,” and the open circles indicate “control by 0.” Postselecting on the ∣+⟩ outcome yields a state with, to first order, an imaginary-time evolution step applied. Given a choice of an appropriate parameter *m*_0_, the time-rescaling factor $s=m_{0}/\sqrt{1-m_{0}^{2}}$ and the rotation $\theta =\kappa \;\arccos\;[m_{0}+\sqrt{1-m_{0}^{2}}/\sqrt{2}]$, where $\kappa =\mathrm{sgn}(m_{0}-1/\sqrt{2})$ [see (*12*)].

In the IPE approach, even a single ancilla (*8*, *68*) is sufficient to learn this phase; a resource cost saving that will be welcome in the early fault-tolerant regime. The method is summarized on the left of Fig. 10. We conditionally apply *N* SO-QFT steps *U^N^*(δ*t*) controlled by an ancillary qubit in the ∣+⟩ state. At the *N*th step, we measure the ancillary qubit in the ∣+⟩ basis, at which point the state is discarded. We see that for an eigenstate, the global phase information is encoded in the relative phase between the ∣0⟩ and ∣1⟩ state of the ancillary qubit

$$\begin{array}{rl} |+\rangle |{\text{Ψ}}_{n}(0)\rangle & \Rightarrow \frac{1}{\sqrt{2}}[|0\rangle |{\text{Ψ}}_{n}(0)\rangle +|1\rangle |{\text{Ψ}}_{n}(t)\rangle ]\\ & =\frac{1}{\sqrt{2}}(|0\rangle +e^{-{iE}_{n}t}|1\rangle )|{\text{Ψ}}_{n}(0)\rangle \end{array}$$ (39)

The probability of finding the ancillary qubit in state ∣+⟩ fluctuates as the phase. We use this to extract a periodic time signal *a*(*t*) where the frequency is proportional to the energy of the simulated wave function

$$a(t)=\cos^{2}\left(\frac{E_{n}}{2}t\right)$$ (40)

Because the number of qubits that we can classically emulate is limited, using the single-ancilla IPE for our demonstrations here is a natural choice. We report the exact evolution of *a*(*t*) plotted at regular time points; this is straightforward since we use classically emulated quantum processors. On a real device, because the single-ancilla projection probability is statistical in nature, the time propagation and measurement will have to be repeated multiple times.

If more ancilla qubits are available, then this naturally extends to the standard Fourier phase estimation; for completeness, we include this in the Supplementary Materials, where we also note the use of classical Fourier analysis to extract features if the hardware is limited to a single ancilla.

#### State preparation

Preparing an appropriate starting state is a crucial and nontrivial component of dynamics simulation. To be consistent with the performance of the subsequent time evolution, we should ideally perform state preparation in polynomial time. Furthermore, since we are working in the first-quantized picture, we must explicitly realize the correct (anti)symmetry therein. The canonical quantum algorithmic approach is to start by initializing an easily prepared reference state and then drive it toward, e.g., the ground state; recent studies (*1*) have highlighted ongoing challenges and uncertainties associated with this task. In this section, we briefly review them and introduce the state preparation methods assessed in this work: a quantum algorithm for preparing antisymmetric states, state preparation with IPE described in the “Energy observable” section in Materials and Methods, and PITE (*12*) for preparing ground states. We provide a brief review of the state preparation literature in the Supplementary Materials.

##### Initial state loading and antisymmetrization

For many-body systems in which most particles are indistinguishable, the challenge of preparing an initial state with proper exchange symmetry is nontrivial. Here, we will assume that we wish to prepare antisymmetric states, but the methods to prepare bosonic symmetric states are near identical. The choice of method to accomplish antisymmetrization determines the options for actually loading the initial single-particle basis states, as we now explain.

Protocols for creating an antisymmetric superposition on a quantum computer have been explored in several publications dating back at least as far as 1997 by Abrams and Lloyd (*69*). The relatively recent 2018 paper of Berry *et al.* (*41*) describes a deterministic, polylogarithmic algorithm to create a state of the form

$$|\mathrm{prm}\rangle \equiv \frac{1}{\sqrt{P!}}\sum_{i}^{P!}{\mathrm{perm}}_{i}(|0\rangle |1\rangle \ldots |P-1\rangle )$$ (41)

Here, we have *P* sets of qubits, each of size *s* = ⌈ ln *P*⌉, which represent the binary numbers 0 to *P* − 1. The notation perm*_i_*() indicates the *i*th permutation and is understood to include the sign appropriate to antisymmetry [that is, (−1)*^k^* if there are *k* pairwise swaps needed to permute from the canonical ascending order].

The state ∣prm⟩ is a superposition of binary labels rather than a superposition of states in the grid-based representation as we require. In principle, we can obtain the latter once we have the former. Specifically, we prepare ∣prm⟩ and distribute each of the *P* sets of qubits as follows: Each will occupy the first *s* qubits of one of our *P* particle-representing registers, while the remaining qubits within each particle register are in state zero, $|0_{\;\_}\rangle$. We now apply to each register an operator that maps these integer numbers to the desired grid-based eigenstates as follows: $M_{\mathrm{unpack}}|(j)\rangle |0_{\;\_}\rangle =|\psi_{j}\rangle$ where ψ*_j_* with *j* = 0…(*P* − 1) are the single-particle basis states from which we are building the initial state. While this is conceptually simple, the operator *M*_unpack_ may be challenging to implement since it must encode knowledge of every ψ*_j_*, and moreover, it should be (near) deterministic if it is to succeed over all *P* particle registers.

Instead, one might wish to begin by preparing a simple product state across our particle registers, i.e.

$$|\psi_{0}\rangle |\psi_{1}\rangle \ldots |\psi_{P-1}\rangle$$ (42)

and then find some means to antisymmetrize this, thus generating a Slater determinant (*70*). The preparation of each state ∣ψ*_j_*⟩ would be relatively easy, performed independently for each particle register and optionally involving nondeterministic approaches with a high success probability per attempt. In the Supplementary Materials, we highlight recent advances in preparing known (analytic) single-particle states ∣ψ⟩ on a quantum register. This route is attractive but relies on an efficient means of subsequently antisymmetrizing the product state in Eq. 42.

In (*31*), the authors propose performing antisymmetrization on a product state using an adaption of the methods in (*69*) (i.e., permuting a product state by cosorting a state of the form ∣prm⟩); however, they do not explicitly specify how the resulting swap flags are to be uncomputed [moving from equations 4 to 5 in (*31*) appears nontrivial]. In the “Antisymmetrization of the initial state” section in Results, we introduce two simple solutions to this issue. We will rely on the following assumption (which may itself be nontrivial): We must specify a Hamiltonian *H*_synth_ with the property that

$$H_{\mathrm{synth}}|\psi_{i}\rangle =E_{i}|\psi_{i}\rangle \;E_{i+1}>E_{i}$$

(reiterating Eq. 7). This Hamiltonian is synthetic in the sense that it need not correspond to any physically legitimate scenario. The condition implies that the states ∣ψ*_i_*⟩ must be orthogonal; moreover, we desire well-separated energies. The “Antisymmetrization of the initial state” section in Results not only specifies the key steps but also gives indicative performance numbers from numerical emulation of antisymmetrization for *P* = 5 particles.

##### Refining the initial state: Ground state preparation

The remarks above concern the initialization of our grid-based machine into a state that we understand and can describe analytically. Of course, we may then wish to drive this state into a more accurate representation of, e.g., the ground state of the real system before exploring its dynamics, assuming nonzero overlap between the initial reference and the target state. While many quantum algorithms have been proposed for this (see the Supplementary Materials), in this work, we demonstrate two techniques that exploit the nonunitary nature of single-ancilla measurements.

1) State editing: Use of ancilla measurements in phase estimation is a well-established technique for ground state preparation (*41*, *67*, *71*). Here, we consider the single-ancilla variant that uses IPE as described in the “Energy observable” section in Materials and Methods, where we regard the ancilla measurement as a midpoint rather than an end goal. We emulate an approach that uses this measurement to remove a known state from superposition to 2D hydrogen, which we report in the “State editing” section in Results.

Suppose that we can prepare an initial state that contains a superposition of eigenstates including our desired state. Let us assume that we know the energy *E*_κ_ of the state that we wish to remove, which can be revealed using the method in the “Energy observable” section in Materials and Methods and classical Fourier analysis. We prepare our ancilla in the ∣+⟩ state and then perform conditional evolution as previously described. To remove state(s) Ψ_κ_ with eigenvalue *E*_κ_, we require measurement at *T*_κ_, such that *e*^−*iE*_κ_*T*_κ_^ = −1, thus

$$T_{\kappa }=N_{\kappa }\delta t=\frac{\pi }{E_{\kappa }}$$ (43)

Now, the undesired component has zero probability of yielding ∣ + ⟩ from the ancilla measurement, so postselecting on that outcome will entirely eliminate its contribution from the register. The more conventional approach for state preparation with phase estimation, which seeks to amplify a target state with known energy, is reviewed in the Supplementary Materials.

2) PITE: The second state preparation method that we explore is a recently introduced approach for performing imaginary-time evolution (*12*, *72*) on quantum computers. The method does not require a priori knowledge of Hamiltonian eigenvalues; however, unfortunately, it does suffer an exponentially vanishing success probability, as the authors note and we presently explore.

Suppose again that we have an initial state that contains a superposition of eigenstates ∣Ψ⟩ = ∑*_n_c_n_*∣Ψ*_n_*⟩. Imaginary-time evolution applies the nonunitary evolution operator $e^{-\hat{H}\tau }$ to the initial state

$$|\text{Ψ}(\tau )\rangle =e^{-\hat{H}\tau }|\text{Ψ}\rangle =\sum_{n}c_{n}e^{-E_{n}\tau }|{\text{Ψ}}_{n}\rangle$$ (44)

and therefore approaches the lowest-energy state in the initial superposition exponentially fast at the long-τ limit. The PITE approximates, to first order, the Hermitian small imaginary-time evolution operator $m_{0}e^{-\hat{H}\delta \tau }$ (where 0 < *m*_0_ < 1 and $m_{0}\neq 1/\sqrt{2}$) applied to the state on the main register, by controlling both forward and reverse real-time evolution operators *U*(δ*t*) and *U*^†^(δ*t*) with a single ancillary qubit prepared in superposition. Postselecting on the ancilla measurement outcome of ∣0⟩ applies the desired evolution on the register. We prepare the same ∣+⟩ ancilla state as per the IPE and start from the circuit in Fig. 10, noting that it achieves the exact same nonunitary operation proposed in (*12*) but with fewer gates. In the “Probabilistic imaginary-time evolution” section in Results, we emulate such a circuit and test it numerically in preparing the ground state of a 2D hydrogen atom.

Similar to state editing by phase estimation, this approach is also probabilistic, and preparing an initial state with large overlap with the ground state is key to maximizing the rate of success. In this case, however, the probability that every sequential ancilla measurement yields the desired outcome decays exponentially with the number of measurements. We refer the reader to (*12*), which introduced the technique and applied it to wave packets in parabolic potentials, for details of this drawback and possible ways to address it. We highlight that, while the current method may be impractical on a real quantum computer, it is nonetheless an effective quantum-inspired ground state preparation method for classical emulations.

#### Attenuation and scattering

In this work, we developed a quantum circuit analog of CAPs, a well-established technique from classical simulation (*60*). Here, we present this method and show that it can also be used to track the probabilities of wave packets being found at specific pixels, which are important to the determination of reaction rates in molecular processes. We test these techniques in the ionization of a single bound electron by a strong applied electric field and two-particle scattering scenarios in the “Quantum dynamics demonstrations” section in Results.

The use of the *k*-space mapping imposes a periodic boundary condition in real space. For nonperiodic systems, this introduces artificial interactions and state interference through the boundary, and one of the requirements on the simulation box is that it is sufficiently large that these have negligible impact on the dynamics. Given that, for fixed spatial resolution, the width can be increased with only a logarithmic cost both in the number of additional qubits and the wall clock execution time (see the “Quantum computer resources and architecture” section in Results), this does not usually pose a problem. However, there are simulation tasks where simply enlarging the box may not be an ideal solution: for example, a study of a scattering process where the quantum states of scattered particles have contributions that travel at speed toward the edges of the simulation box.

One alternative is to attenuate wave function amplitude near the boundary. Any such attenuation would be a nonunitary process involving measurement and implying some probabilistic element; however, the cost associated with any need for repetition may be preferable to the increase in the number of qubits required in the former approach. Furthermore, we show that from the rate of failure of this probabilistic process, one can infer the probability of a particle being measured at a given pixel.

The interaction potential part of the Hamiltonian ${\hat{H}}_{\mathrm{pot}}$ is modified to contain both a real and (negative) imaginary part

$${\hat{H}}_{\mathrm{int}}={\hat{H}}_{U}+{\hat{H}}_{V}-i\hat{V}$$ (45)

The time evolution operator is thus

$$\text{Ψ}(r,t)=e^{-i\hat{H}t}e^{-\hat{V}t}\text{Ψ}(r,0)$$ (46)

The absorbing potential $\hat{V}=V(r)$ should be balanced between sharp, abrupt attenuation, which reduces the size of the attenuation region while suppressing transmission, and smooth, gradual attenuation, which suppresses reflection. For brevity, we will only assert that it is possible to construct CAPs that are completely reflection and transmission free (*73*) and refer the reader to the rich body of classical quantum dynamics literature on complex potentials (*74*–*79*).

On a quantum computer, the objective is to achieve the following nonunitary transformation in (although not limited to) the real-space representation at every SO-QFT step

$$\sum a_{n}|n\rangle \Rightarrow \sum a_{n}e^{-V(x_{n})\delta t}|n\rangle$$ (47)

followed of course by the necessary renormalization. Here, *x_n_* is the location of the peak of the pixel function $P_{n_{r}}^{n}(x)$ as per Eq. 25. Of course, δ*t* is merely a constant, corresponding to the temporal granularity of the simulation. The analogous mapping should occur for *y* and *z* and for each particle in the system; ideally, this should be done in a fashion that perfectly preserves any exchange symmetry relevant to the system.

To achieve this, we could proceed as follows for each *x_n_* that lies within our attenuating region [i.e., where *V*(*x_n_*) ≠ 0]. At the end of each spatial part of our SO-QFT cycle, we prepare an ancilla qubit in state ∣0⟩, and we perform a multicontrolled rotation *e*^*i*σ*_x_*θ^ on that ancilla. The rotation occurs if and only if the subregister is exactly in state ∣*n*⟩, which corresponds to the pixel function peaked at *x_n_*. We then measure the ancilla. If we obtain outcome ∣1⟩, we say that the particle in question has been measured to be that point, and we may either halt the simulation or proceed with the remaining particles—both scenarios are examined in the numerical emulations presented. However, if we obtain outcome ∣0⟩, then the simulation simply proceeds, except that the amplitude of state ∣*n*⟩ has been attenuated from *a_n_* to *a_n_* cos (θ) (and the state has been renormalized). By repeating this process for all *x_n_* within the attenuating region, selecting the proper θ = arccos [*e*^−*V*(*x_n_*)δ*t*^] in each case, we implement Eq. 47 (see middle of Fig. 10). In parallel, the same process can be applied for other subregisters and other particles.

If the attenuation region is narrow, corresponding to only a few values of *x_n_*, then the above protocol can be relatively efficient. Remarkably frugal constructions for multicontrolled Paulis do exist (*39*), and of course, a controlled Pauli can implement the general θ rotation described above with the use of additional single-qubit rotations. However, if the attenuation region is a main portion of the simulation box, then the method would be exponentially inefficient as it requires action for each element of the spatial superposition. Fortunately, however, one can simply set a uniform attenuation strength *V* over a range of *x_n_* values and implement it in one step using an appropriate subset of register qubits in the control process. The simplest example occurs when the width of the attenuating region is 2^−*m*^*L* for some integer *m* (*L* being the width of the simulation box). In this case, only the first *m* qubits from the subregister control the rotation. This is the approach taken in the numerical emulations presented in this work.

By repeating the entire simulation multiple times and keeping track of the probability that the ancilla qubit yields ∣1⟩, we are also recording the time-dependent probability that a particle has reached a given pixel in the simulation box. One could, in principle, obtain this information by generating the probability distribution of particles at different times through direct sampling of particle subregisters; we generate plots that would be obtained this way. However, on a real quantum computer, selecting specific pixels and entangling them with a single qubit that would then be measured is a much more cost-efficient statistical approach. One might envisage the determination of reaction rates by selecting key pixels, much like the selection of a reaction coordinate in traditional reaction dynamics, and using an equivalent approach to track the probability that a particle wave packet crosses said pixels. We show a proof of concept here by directly measuring particle scattering and ionization probabilities, in combination with CAPs.

#### Coulomb potential: Demands on spatial and temporal resolution

Here, we review considerations regarding the granularity of simulating the Coulomb potential using either the classical or quantum SO; a more detailed discussion can be found in the Supplementary Materials. The SO uses finite spatial basis set and discrete time approximations, which become exact in the limit of infinite resolution. To optimize computation cost, it is sensible to use the minimum finite resolution necessary for obtaining physically accurate results. The spatial discretization determines the fidelity of a model’s state and (potential) operators to reality and bounds the maximum attainable accuracy of measurable properties. The time resolution is chosen such that the Trotter error during time propagation is sufficiently low; however, because the error term of any Trotter sequence depends on the commutator between ${\hat{H}}_{\mathrm{kin}}$ and ${\hat{H}}_{\mathrm{int}}$, which, in turn, depends on the spatial derivatives of the model potential, using a fine spatial resolution will therefore require commensurately fine time stepping to suppress Trotter error during propagation (*65*).

For propagation under smooth (e.g., harmonic) potentials, low spatial and time resolutions are generally sufficient. On the other hand, because of the sharp singularity in the Coulomb potential, accurately describing wave function propagation near the singularity is more involved, even if we are to effectively model a capped potential by never collocating the origin at a grid point. We expect that a high spatial resolution is necessary to attain accurate operator physics for states with large amplitudes at the singularity, which will demand a high time resolution to control propagation errors. In the “Spatial and temporal resolution” section in Results, we use numerical studies to estimate the minimum spatial and temporal resolution required for sufficiently accurate time propagation of chemically relevant systems, using the first-order SO-QFT.

#### Augmented split-operator

We now describe an alternative means to tackle simulation of states that may have large amplitudes at the Coulomb singularity instead of using a brute force increase in spatial and temporal resolution. We call the approach the ASO as it involves introducing an additional step in the SO-QFT cycle. The results of applying the protocol below with an emulated quantum computer are presented in the “Augmented split-operator” section in Results.

Following the implementation of the exponential operators in a SO-QFT time step and while still in the real-space representation, we apply a core stabilization process. This is intended to “fix” the most severe deviations of the applied unitary from the ideal unitary $e^{-i{\hat{H}}_{\mathrm{tot}}\delta t}$. We emphasize that the fix is at the operator level and is agnostic to the specific state that is being modeled. However, the motivation is to make the dynamics of core-peaked states more accurate. We aim to reduce the overall time cost of a simulation with a given accuracy, without increasing the qubit count.

We describe the process for a simulation of a single particle and comment on the generalization to *N* particle systems further below. The following is performed on a classical computer, as a one-off preparation for our simulation. We make a fixed choice of δ*t*, and we must repeat the analysis for each value of δ*t* that we wish to use.

1) We compute the ideal iterate $U_{\mathrm{ideal}}=e^{-i{\hat{H}}_{\mathrm{tot}}\delta t}$ and the actual iterate defined by Eq. 32 viz.

$$U_{\mathrm{SO}}(\delta t)=e^{-iD_{\mathrm{int}}\delta t}(U_{\mathrm{QFT}}^{†}\;e^{-iD_{\mathrm{kin}}\delta t}\;U_{\mathrm{QFT}})$$

These objects are computed in the full basis of pixel functions for the particle (and are therefore far from diagonal).

2) We examine *U*_repair_, which is defined by

$$U_{\mathrm{ideal}}=U_{\mathrm{repair}}U_{\mathrm{SO}}(\delta t)$$

and we will find that *U*_repair_ is close to the identity except for elements corresponding to pixel functions that are near the Coulomb singularity. Therefore, we select a set of *Q* such pixels and derive a small *Q* × *Q* unitary *U*_core_ that closely matches *U*_repair_ in that subspace.

3) We define augmentation *U*_aug_ as the identity operator except for the *Q*-state subspace, where it corresponds to *U*_core_.

4) We find a circuit *C*_aug_ that can implement *U*_aug_ to a good approximation. This circuit is not merely a set of phase operations since *U*_aug_ is not diagonal in the *Q*-state subspace; *C*_aug_ will cause a flow between the amplitudes of those states.

This classical analysis is nontrivial but tractable since the unitaries involved are “only” of size 2*^n_r_D^* for a *D*-dimensional problem where *n_r_* is the number of qubits per subregister. Moreover, there are helpful symmetries in the analysis, and notwithstanding the description in step 1, we need not compute *U*_ideal_ and *U*_SO_ entirely but only in the subspace where we are likely to identify the *Q* core pixels. With these steps completed, we are in a position to perform simulations with the ASO iterate

$$\begin{array}{rl} U_{\mathrm{ASO}}(\delta t) & \equiv U_{\mathrm{aug}}\;U_{\mathrm{SO}}(\delta t)\\ & =U_{\mathrm{aug}}\;e^{-{iD}_{\mathrm{int}}\delta t}(U_{\mathrm{QFT}}^{†}\;e^{-{iD}_{\mathrm{kin}}\delta t}\;U_{\mathrm{QFT}}) \end{array}$$ (48)

In Results, we report on the performance of this method for two cases, 2 × 2 and 4 × 4 pixels. As demonstrated by those examples, even with such small corrections, the method is quite effective, and the cost of the augmentation can be modest: The circuit *C*_aug_ is compact and need only target a small subset of qubits, with the others acting as controls.

The generalization of the ASO method to the *P* particle case is straightforward except for a caveat. The main time cost of the SO-QFT method lies in the implementation of the $e^{-i{\hat{H}}_{\mathrm{int}}\delta t}$ part, because of the *P*(*P* − 1)/2 particle pairings involved. The ASO method can be efficiently implemented by applying an *U*_aug_ augmentation after each such pairing, subsequent to implementing the *e*^−*iC*δ*t*/∣**r**∣^ and while the registers are still representing relative coordinates *x_i_* − *x_j_*, *y_i_* − *y_j_*, etc. Because *U*_aug_ is not diagonal, ensuring the proper evolution requires implementing the full orthogonal transformation to relative and central coordinates

$$\left\{x_{i},x_{j}\}\Rightarrow \{x_{i}-x_{j},x_{i}+x_{j}\}\;\text{rather than}\;\{x_{i}-x_{j},x_{i}\right\}$$

and similarly for *y* and *z*. Fortunately, the additional arithmetic step that this implies is trivial.

## Supplementary Materials

### This PDF file includes:

Sections S1 to S8 Figs. S1 and S2 Table S1 References
