# Supplementary material for: Grid-based methods for chemistry simulations on a quantum computer
Source: Sci Adv. 2023 Mar 1;9(9):eabo7484. doi: 10.1126/sciadv.abo7484 (PMC9977186; doi:10.1126/sciadv.abo7484)
Supplement: Supplementary file 1 — Sections S1 to S8 Figs. S1 and S2 Table S1 References [file sciadv.abo7484_sm.pdf]

Science Advances
AAAS

Supplementary Materials for

Grid-based methods for chemistry simulations on a quantum computer

Hans Hon Sang Chan *et al.*

Corresponding author: Hans Hon Sang Chan, hans.chan@materials.ox.ac.uk

Sci. Adv. **9**, eabo7484 (2023)
DOI: 10.1126/sciadv.abo7484

This PDF file includes:

Sections S1 to S8
Figs. S1 and S2
Table S1
References

SUPPLEMENTARY MATERIALS

S1. BACKGROUND

In seeking to use a controlled quantum machine as our simulator, one may adapt the rich set of numerical methods for solving the time dependent Schrödinger equation on 'classical' (pre-quantum) computing platforms. Though many state representation and time propagation schemes exist [20–22, 63, 79, 81, 82], the one of particular interest here is the dynamic Fourier or split-operator Fourier transform (SO-FT) method, which dates back at least as far as the work of Feit and Fleck in 1976 [30, 58, 59]. The scheme is popular and often employed in small-molecule applications, predominantly in propagating nuclear wavepackets on pre-calculated static or time-dependent electronic potential energy surfaces. It has found uses in reactive scattering [83, 84] and excited-state chemistry [85–89], as well as modelling quantum processes like tunnelling [90, 91] and superfluid turbulence [92], and indeed quantum control for designing quantum devices [93, 94]. Application to the relativistic Klein-Gordon equation [53] and non-linear Schrödinger equation [95–97] extends the method's scope. Its success is partly attributed to the simplicity of its construction, as it only involves updating the vector describing the state, and partly to the low computational price paid for obtaining efficiently converging results [98].

Building on the works of Zalka [4] and Wiesner [3] from the mid 1990's, it was observed just over a decade ago by Kassal *et al.* [5] that it will be advantageous to store high-dimensional spatial grid representations of molecular wavefunctions on digital quantum computers using qubits that scale only linearly with the degrees of freedom. They also showed that, by exploiting the remarkably efficient quantum Fourier transform (QFT), a quantum version of the SO method (the SO-QFT) will be able to simulate the dynamics of a chemical Hamiltonian in polynomial time. Indeed, the authors challenged the *status quo* of limiting the split-operator method to propagating nuclei on electronic potential energy surfaces.

Although the SO-QFT method is oriented towards fault-tolerant quantum computing, it is nonetheless gaining increasing interest. The method has been investigated in the context of propagating one-dimensional Gaussian wavepackets [6], quantum harmonic oscillator [8], non-adiabatic dynamics across coupled, pre-computed potential energy surfaces [10], imaginary-time evolution for molecular geometry optimisation [17] as well as ground state, Gibbs state and partition function determination [12]. We highlight works geared towards understanding the scaling of the SO-QFT at mid-to-large scale, specifically that of Jones *et al.* which looked at the resource necessary for a fault-tolerant implementation [7], the extensive investigation into first quantized basis-set simulations for chemistry of Su *et al.*, which also included block-encoding the SO-QFT Hamiltonian for simulation using qubitization and interaction picture techniques [11], and analysis by Childs *et al.* which provided concrete upper bounds for the gate complexity of SO-QFT to arbitrary order [13]. Other attempts at direct quantum simulations in real-space grids include using finite difference stencils to approximate the kinetic operator in second quantization [99], and in first quantization with propagation based on the truncated Taylor series algorithm [9], as well as a Cartesian component-separated approach [14].

S2. THEORETICAL FRAMEWORK

A. Representations in momentum- and real-space

We wish to model the dynamics of a multi-particle problem with Hamiltonian $\hat{H}_{\text{tot}}$. We divide the qubits of our quantum computer into registers, each associated with one of our particles, then further divide each register into sub-registers corresponding to the dimensions of the model. In general, the registers corresponding to different particles need not be the same size, nor do the sub-registers need to match in size. Indeed, many scenarios would naturally suggest variations, e.g. a nuclear particle may be adequately modelled within a smaller 'box' than its bound electrons, or we might model e.g. a solid-state interface where the z-direction is restricted versus the x-y plane. In the present work we confine ourselves to considering cases where all sub-registers are of equal size, so that the computer has size of order $O(d\,P\,n_r)$ (neglecting ancillas used in temporary roles) where d is the spatial dimensionality of the problem (2 or 3 for us), P is the number of particles (1 or 2 in our simulations), and n_r is the number of qubits in each sub-register.

We now elaborate on the finite basis (plane-wave) and discrete variable (real-space) representations described in the introduction. Suppose that we wish to model a 1D system which is well-localised within a region $-\frac{L}{2} < x < \frac{L}{2}$. We refer to this region as the 'simulation box' and thus L is the box width. For now we will assume that the real system's state has negligible amplitude outside this box, both initially and throughout the anticipated simulation. This condition is relaxed presently when we consider scattering and ionisation. We choose a k-space (or 'spectral') representation in which a plane wave basis state of the modelled system is represented by a state of the computer's sub-register as

$$\phi_k(x) = L^{-\frac{1}{2}} \exp\left(\frac{i2\pi kx}{L}\right) \quad \leftrightarrow \quad |k\rangle\,. \tag{S1}$$

Note that a negative value of k implies the two's complement binary representation. The same meaning is intended whenever we write a Latin letter in a ket.

Defining $\rho = 2^{n_r-1}$ and noting that we have 2ρ basis states in our computer's sub-register, a natural choice for the allowed k is to run from $-\rho$ through zero to $\rho-1$. With this choice subsequent expressions have a compact form; but the mapping from plane wave to computational basis state can employ a shift so that k runs from (say) 0 to $2\rho-1$. Indeed, in the simulations performed presently, underlying code using both conventions is tested. The eventual choice will be a matter of optimising circuit depths in the real quantum processor. As a further aside, note that an interesting alternative to Eqn. S1 is to use $2\pi(k+1/2)$ rather than $2\pi k$. Then any state of the model must satisfy $\Psi(x=L/2) = -\Psi(x=-L/2)$. Therefore state Ψ of a 1D modelled system would be represented by our

sub-register $|\psi\rangle_x$ according to

$$\Psi = L^{-\frac{1}{2}} \sum_{k=-\rho}^{\rho-1} a_k e^{i2\pi kx/L} \quad \leftrightarrow \quad |\psi\rangle_x^{\mathrm{KS}} = \sum_{k=-\rho}^{\rho-1} a_k |k\rangle \,. \tag{S2}$$

Formally this is a bijective encoding of the Fourier components of the modelled state as computational basis states. Throughout this paper we use the double-headed arrow $\leftrightarrow$ to indicate such a mapping: encoding of Fourier components (modelled system) on the left, to the quantum computer's state on the right. Practically, the meaning of $\leftrightarrow$ is that when we apply operations to the qubit register, we will do so with our mapping to the modelled system in mind; this will be clear presently. We use the superscript KS for k-space. For simplicity we do not put '$p = 1$' in the subscript, but we understand this is for a given particle.

Our choice of mapping in Eqn. (S2) together with the equivalent representations for other particles and dimensions allows our quantum computer to represent an arbitrary k-space wavefunction of the multi-particle system, up to the limitation that plane wave components of spatial frequency higher than 2^{n_r-1} are omitted. Note that the represented state is periodic in space, since $|\psi\rangle_{x+1} = |\psi\rangle_x$ and similarly for other sub-registers. Throughout this paper we restrict our interest to the range $-L/2 < x < L/2$ and refer to this as the 'simulation box' with L therefore the simulation box width. In the present section we have, and will, set $L = 1$ in order to keep the expressions in a clean form. The periodicity is not exploited but we do need to be mindful of it in, e.g., the scattering/ionisation modelling described.

It would be possible to exclusively use the k-space representation in grid-based modelling, and indeed there may be advantages to doing so [11, 24]. However, in the present study we employ the approach in which the registers are periodically transformed into a 'dual' representation [99]. Specifically, we apply to each sub-register a quantum Fourier transform (QFT) denoted by U_{QFT} and defined by the circuit shown in Fig. S1:

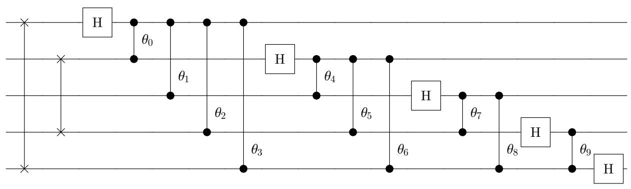

FIG. S1. The QFT circuit used to transform from the k-space to the real-space representation. The circuit shown is for a 5-qubit sub-register.

The sub-register as a whole will be transformed as

$$|\psi\rangle_x^{\mathrm{RS}} = U_{\mathrm{QFT}} |\psi\rangle_x^{\mathrm{KS}} = \sum_{n=-\rho}^{\rho-1} b_n |n\rangle \,, \tag{S3}$$

where

$$b_n = \frac{1}{\sqrt{2\rho}} \sum_{k=-\rho}^{\rho-1} \exp\left(i\frac{n\pi}{\rho}k\right) a_k. \tag{S4}$$

The superscript RS indicates that we call this the real-space mode of representation; we will justify this label momentarily.

When we wish to return to the original, k-space representation we employ the inverse QFT:

$$|\psi\rangle_x^{\mathrm{KS}} = U_{\mathrm{QFT}}^{-1} |\psi\rangle_x^{\mathrm{RS}} = \sum_{k=-\rho}^{\rho-1} a_k |k\rangle \,, \tag{S5}$$

where of course

$$a_k = \frac{1}{\sqrt{2\rho}} \sum_{n=-\rho}^{\rho-1} \exp\left(-i\frac{k\pi}{\rho}n\right) b_n. \tag{S6}$$

We can now ask, what wavefunction must be represented by each computational basis state $|n\rangle$ appearing in Eqn. (S3)? We can answer by setting all b coefficients to zero except for b_n (i.e. $b_m = \delta_{m,n}$), transforming to k-space using Eqns. (S5) and (S6), and finally referring back to our original declared mapping in Eqn. (S1). We find that each $|n\rangle$ maps to a wavefunction peaked at (but not strictly localised around) the spatial point $x_n = \frac{n}{\rho}\frac{L}{2}$. Specifically, the inferred mapping is

$$\phi_n(x) = P^n_{n_r}(x) \quad \leftrightarrow \quad |n\rangle \tag{S7}$$

with

$$P^n_{n_r}(x) = \exp\left(-\frac{i\pi x'}{L}\right) \sqrt{\frac{2}{\rho L}} \sum_{j=1}^{\rho} \cos\frac{\pi(2j-1)x'}{L} \tag{S8}$$

where $x' = x - x_n$ with $x_n = \frac{nL}{2\rho}$, and $\rho = 2^{n_r} - 1$.

Within our simulation box $-\frac{L}{2} < x < \frac{L}{2}$, the function $P^n_{n_r}(x)$ serves the role of an approximation or "smear" of the Dirac delta function $\Delta(x - x_n)$, the sharpness increasing as 2^{n_r} tends to infinity (strictly speaking it tends to a Dirac comb since the function is periodic beyond the simulation box; the function can also be recognised as a difference of two Dirichlet kernels e.g. for $L = \pi$, $P^0_{n_r}(x) \propto D_{2\rho}(x) - D_\rho(2x)$). Shortly we will motivate the informal term 'pixel functions' for our $P^n_{n_r}(x)$. The separation between the peaks of adjacent pixel functions, e.g. $x_2 - x_1$, is $\delta r = L/2^{n_r}$, and we now define the model's *spatial resolution* as the reciprocal of this quantity, i.e. as the number of pixel functions per unit distance:

$$\delta r^{-1} = 2^{n_r}/L. \tag{S9}$$

For the present paper, we note that there are four key observations regarding the set of spatial wavefunctions $P^n_{n_r}(x)$ which are true for any given qubit count n_r. They are mutually orthogonal, each is peaked at x_n, and importantly each is zero at all points $x_{m\neq n}$. That is to say, where a given spatial wavefunction has its primary peak, all other wavefunctions are strictly zero. Finally, we note that at its peak each function has the value

$$P^n_{n_r}(x = x_n) = \frac{1}{\sqrt{\delta r}}.$$

These properties mean that a suitable decomposition to represent any 1D wavefunction $\Psi(x)$ in our quantum register is very intuitive:

$$\Psi(x) \approx \frac{C}{\sqrt{\delta r}} \sum_{n=-\rho}^{\rho-1} \Psi(x_n) P^n_{n_r}(x) \leftrightarrow \frac{C}{\sqrt{\delta r}} \sum_{n=-\rho}^{\rho-1} \Psi(x_n) |n\rangle \tag{S10}$$

i.e. the required amplitude of $|n\rangle$, the state representing the wavefunction peaked at point x_n, is found simply by sampling

the target wavefunction at that point. Here C is a normalisation constant that will be close to unity providing that (a) the target wavefunction has negligible amplitude outside of the simulation box and (b) the target wavefunction varies slowly with respect to δr. If C differs significantly from unity then the model does not properly capture $\Psi(x)$, however C can be above or below unity and $C = 1$ does not imply optimality.

So far we have considered the role of one sub-register, which corresponds to one dimension of a given particle. The generalisation to 2D or 3D is the natural one: the sub-registers tensored together to form the complete representation of a given particle. The 3D analogue is

$$\begin{aligned}\Psi(\mathbf{r}) &\approx C\delta r^{-\frac{3}{2}}\sum_{n,m,l=-\rho}^{\rho-1}\Psi(x_n,y_m,z_l)P_{n_r}^n(x)P_{n_r}^m(y)P_{n_r}^l(z)\\ &\leftrightarrow C\delta r^{-\frac{3}{2}}\sum_{n,m,l=-\rho}^{\rho-1}\Psi(x_n,y_m,z_l)\left|n\right\rangle\left|m\right\rangle\left|l\right\rangle. \qquad \text{(S11)}\end{aligned}$$

(Our 2D numerical results of course omit the third sub-register.) When we generalise to represent a P-particle wavefunction $\Psi(\mathbf{r}_1, ..., \mathbf{r}_P)$ we need only extend in the natural fashion,

$$C\delta r^{-\frac{3P}{2}}\sum_{\{n_1..l_P\}=-\rho}^{\rho-1}\Psi(x_{n_1},y_{m_1},...,z_{l_P})\left|n_1\right\rangle\left|m_1\right\rangle...\left|l_P\right\rangle. \qquad \text{(S12)}$$

The first figure in the Introduction section of the main text shows the ground state of 2D hydrogen plotted from the analytic solution (lower left side, cyan) and constructed as a approximation using the 2D variant of Eqn. S11 for the case of $n_r = 6$ qubits per sub-register (right side, orange). This wavefunction will be discussed in more detail presently, and is one of the initial states we will use in our numerical simulations. It is apparent by inspection that the approximation is good over the majority of the simulation box. The analytic state of course extends beyond the box, where our modelled state is undefined; taking the modelled state to be zero there, the fidelity of the modelled state with respect to the analytic state (integrating over all space) is 0.99946. If instead we project the analytic state into the box and renormalize it, then the infidelity between the model and the analytic solution falls below 10^{-4}. The discrepancy is primarily localised at the Coulomb singularity, where the analytic expression has a gradient discontinuity. Of course, the reconstruction must have a continuous gradient (since all constituent functions have this property). The sharpness of the approximation is characterised by δr^{-1} and so increases exponentially with n_r.

We also show examples of $P_{n_r}^n(x)$ for the same case of $n_r = 6$ (but with the phase component removed so that the plots can be seen without an imaginary axis) in the same Figure. Recall that in our two's complement notation, integer indices can denote negative or positive coordinates; the register state $|00..0\rangle = |n = 0\rangle$ corresponds to the wavefunction peaked at the origin (the blue lines in the left and middle panels). The orthogonality property is clear from the middle panel, showing $|n = 0\rangle$ in blue and $|n = 1\rangle$ in orange.

Intuitively, one can think of these spatial wavefunctions analogously to pixels as used in conventional digital photographs: A pixel has a single colour corresponding to the (real, continuous) scene sampled at that point. Similarly each spatial basis state $|n, m, l\rangle$ in Eqn. S11 is associated with a single complex number corresponding to the continuous wavefunction sampled at that point. A photo should have sufficient pixels that all features of interest to the viewer are fully captured, and our simulation should have sufficiently many spatial basis states that all features of the wavefunction are adequately captured. Because of this close analogy, we sometimes refer to the $P_{n_r}^n$ wavefunctions as 'pixel functions'. As we will see presently, a moderate resolution δr^{-1} can prove to be sufficient and thus the number of qubits required can be remarkably low.

It is obvious that pixel wave functions resemble standard sinc functions; indeed if we increase the number of qubits n_r while commensurately increasing L so that the resolution δr^{-1} is held constant, then the pixel function $P_{n_r}^0$ tends to a sinc function in the interval $[-\frac{L}{2}, \frac{L}{2}]$. This is reminiscent of Whittaker-Shannon interpolation and indeed there is a close association between the grid-based quantum model and the theory of signal processing.

The representation described above involves simple Cartesian grids in momentum-space and position-space, with the conversion effected by a QFT; in principle, other coordinate systems could be used (cylindrical, spherical etc) or alternative transformations [100]. In the present paper we will consider the hybrid coordinates needed to evaluate particle-particle interactions.

A spin degree of freedom is trivial to add into the simulation: for spin one-half particles we would simply require a single additional qubit per complete register, while a spin-S particle would require $\ln(S)$ qubits. However in our results, we do not consider any Hamiltonians with a spin-dependent term and therefore we do not model the particle's spin. Importantly, particle symmetry is properly preserved by the SO-QFT; symmetry can be confirmed at any stage using e.g. a SWAP test [101] on the registers representing the relevant pair of particles.

B. Split-Operator Time Propagation

We now make some remarks regarding the SO-QFT method. We first note that Both the first-order $(O(\delta t^2))$ and second-order $(O(\delta t^3))$ Trotterisation split-operator have well-documented numerical advantages [61–63, 82], and higher order Lie-Trotter-Suzuki sequences can also be relevant [64, 65]. The real and k-space Cartesian grid dual representations are natural options for state representation when computing the approximate time evolution operator. In the k-space representation, the kinetic part of the Hamiltonian $\hat{H}_{\text{kin}}$ is separable and exactly local (diagonal). In the real-space representation the interaction part of the Hamiltonian $\hat{H}_{\text{int}}$, is approximately diagonal.

On a quantum computer it is relatively efficient to switch between the two representations using the QFT; the number of gates required per sub-register is quadratic in its number of qubits n_r, and all sub-registers can be transformed independently. Thus we can implement the two parts of the SO cycle each in their preferred, diagonal basis. We perform the following to approximate a time step δt using our quantum computer:

$$|\psi(t+\delta t)\rangle^{\text{RS}} = U_{\text{SO}}(\delta t)\ |\psi(t)\rangle^{\text{RS}}$$

where we recall that the superscript RS denotes the real space

representation, and

$$U_{\rm SO}(\delta t) = e^{-iD_{\rm int}\delta t}\left(U^{\dagger}_{\rm QFT}\; e^{-iD_{\rm kin}\delta t}\; U_{\rm QFT}\right). \qquad \text{(S13)}$$

Here $D_{\rm kin}$ and $D_{\rm int}$ are diagonal real matrices as explained above, and $U_{\rm QFT}$ is the quantum Fourier transform applied to all sub-registers.

We now discuss the evaluation of these operators on quantum computers in detail. We observe that (regardless of basis) any period of evolution under purely the kinetic part $\hat{H}_{\rm kin}$, i.e. any operator

$$U_{\rm kin}(\delta t) = e^{-i\hat{H}_{\rm kin}\delta t}$$

will separate exactly into a product of operators acting independently on each particle and in each dimension:

$$U_{\rm kin}(\delta t) = \prod_{p=1}^{P}\prod_{q\in\{x,y,z\}} \exp\left(i\frac{\hbar^2\delta t}{2m_p}\frac{\partial^2}{\partial q^2}\right)$$

because these components commute. Now suppose that a given quantum sub-register is currently in the k-space representation defined by Eqn (S2), and recall Eqn. (S1) for the meaning of each computational basis state, viz.

$$\phi_k(x) = L^{-\frac{1}{2}}\exp\left(\frac{i2\pi kx}{L}\right) \quad \leftrightarrow \quad |k\rangle\,.$$

We see that the proper way to account for the action of $U_{\rm kin}(\delta t)$ on the molecule is to introduce phases onto our computational basis states according to

$$|k\rangle \;\Rightarrow\; e^{iC\delta t\, k^2}\,|k\rangle \qquad \text{(S14)}$$

with constant $C = 2\hbar^2\pi^2/(L^2 m_p)$. Thus our task is simply to apply phase operations to our quantum computer, independently for each sub-register (and in parallel if our hardware has that capability). Each element of the matrix $D_{\rm kin}$ appearing in Eqn. (S13) is just the sum of $C(k_x^2+k_y^2+k_z^2)$ over all particles. It is trivial to implement the required phases as, for example, a sequence of single- and two-qubit phase gates (see for example [7, 10]). The number of gates required goes as the square of the number of qubits in the register.

We now consider the challenge of modelling evolution under the potential parts of $\hat{H}_{\rm tot}$. Consider first $\hat{H}_U$, which represents the energy of each particle in classical fields. We are primarily interested in an attractive Coulomb potential representing a nucleus (although we discuss a variation including a static electric field presently). For the Coulombic case we write

$$\hat{H}_{\rm U} = \sum_{p=1}^{P}\frac{Q_p}{|\mathbf{r}|_p} = Q\sum_{p=1}^{P}\frac{1}{\sqrt{x_p^2+y_p^2+z_p^2}}. \qquad \text{(S15)}$$

Here we took Q outside of the sum since all Q_p are the same in our atomic and molecular systems of interest; however there would be no difficulty in retaining distinct values. As before, we can write an time evolution operator

$$U_{\rm U}(\delta t) = \prod_{p=1}^{P}\exp\left(-i\frac{Q\delta t}{\sqrt{x^2+y^2+z^2}}\right).$$

The operations are independent between the registers corresponding to different particles, but not independent between sub-registers assigned to a given particle.

We will be in the real space representation when we implement the corresponding dynamics. Recall that the computational basis states of each sub-register will thus correspond to locally-peaked 1D single-particle wavefunctions,

$$\phi_n(x) = P^n_{n_r}(x) \quad \leftrightarrow \quad |n\rangle$$

with each such 'pixel function' having its primary peak at $x_n = nL/2^{n_r} = n\delta r$. Similar expressions describe the roles of the y sub-register states $|m\rangle$, peaked at y_m, and the z sub-register states $|l\rangle$ peaked at z_l.

We proceed to apply a period of time evolution under $\hat{H}_U$ using an approximation that *would* be exact in the limit of infinite spatial resolution. In that case we could apply a series of phase changes to our quantum registers to properly describe any time step δt.

$$\begin{aligned} |n\rangle\,|m\rangle\,|l\rangle \;\;\Rightarrow\;\; & \exp\left(\frac{-iQ\,\delta t}{\sqrt{x_n^2+y_m^2+z_l^2}}\right)|n\rangle\,|m\rangle\,|l\rangle \\ = \;\; & \exp\left(\frac{-iQ\,\delta t}{\delta r\sqrt{n^2+m^2+l^2}}\right)|n\rangle\,|m\rangle\,|l\rangle \quad \text{(S16)} \end{aligned}$$

We see that the task of implementing the latter is only modestly more complex than the former: Now, we need to compute on an entire register of all three sub-registers and the phases required are inverse-square-root instead of merely square. Efficient evaluation of functions such as the inverse-square-root on quantum computers is an active area of development. In particular, Kassal *et al.* [5], Jones *et al.* [7] and Häner *et al.* [66] proposed procedures for reversible computation of the inverse-square-root using fixed-point arithmetic and Newton-Raphson iteration. Polynomial function interpolation using quantum read-only memory (QROM) type circuits is another promising approach [102]. Recent work from Poirier *et al.* [14, 103] combined with other formulations of the Coulomb potential may also ease the efforts of computing the inverse-square-root. We refer to [7, 66] for resource estimates, but here it suffices to note that the number of gates required can potentially scale merely quadratically with the number of qubits n_r. We discuss this further in Supplementary S3.

We motivated the expression above by noting that this would be the exactly correct process if our spatial states were Dirac delta functions. Those states in fact have finite spatial extent but we can expect that, for any sufficiently short time δt and given a smoothly varying potential, an adequate spatial resolution δr^{-1} will result in dynamics that converge to the exact behaviour. However the Coulomb potential is singular at $\mathbf{r}=\mathbf{0}$ and consequently we will need to investigate the behaviour in this region carefully. As we presently explain, it is possible to augment the basic SO-QFT cycle of kinetic and potential evolution with a third phase, whose purpose is to stabilise behaviour at the core singularity.

Finally we must consider the part of the Hamiltonian corresponding to particle-particle interactions $\hat{H}_{\rm V}$. This is straightforward to handle using the same approximation above, i.e. by updating our registers *as if* the states they represent are Dirac delta functions. Then the operator,

$$U_{\rm V}(\delta t) = \exp\left(i\delta t\sum_{p,q=1;p\neq q}^{P}\frac{q_{p,q}}{|\mathbf{r}_p-\mathbf{r}_q|}\right) \qquad \text{(S17)}$$

will be approximated as having diagonal form (and will therefore commute with the $U_U(\delta t)$ operator). Consider the two

registers, each composed of three sub-registers, which represent a given pair of particles $p = 1$ and $p = 2$. Then basis states will be updated as

$$|n_1\, m_1\, l_1\rangle\, |n_2\, m_2\, l_2\rangle \;\Rightarrow\; e^{-i\Theta}\, |n_1\, m_1\, l_1\rangle\, |n_2\, m_2\, l_2\rangle$$

where

$$\begin{aligned}\Theta &= \frac{Q\,\delta t}{\sqrt{(x_{n_1} - x_{n_2})^2 + (y_{m_1} - y_{m_2})^2 + (z_{l_1} - z_{l_2})^2}} \\ &= \frac{Q\,\delta t}{\delta r \sqrt{(n_1 - n_2)^2 + (m_1 - m_2)^2 + (l_1 - l_2)^2}} \end{aligned} \tag{S18}$$

One means of computing the required phases is discussed in the main text. We would pair every particle with a partner and perform the following process (which can occur simultaneously over each pair of registers), before repeating with a different pairing, and so on until all pairings are considered. For each pair, we perform the computation

$$\begin{aligned}&|n_1, m_1, l_1\rangle\, |n_2, m_2, l_2\rangle \;\Rightarrow \\ &\quad |n_1 - n_2, m_1 - m_2, l_1 - l_2\rangle\, |n_2, m_2, l_2\rangle \end{aligned} \tag{S19}$$

which is a straightforward instance of quantum arithmetic on pairs of sub-registers. One suitable method uses the QFT which of course our machine will already have been optimised for [104]. This approach is frugal in terms of the qubit count: we need only a single additional qubit to fully represent each difference $n_1 - n_2$, etc (as the maximum magnitude is within a factor of two of the maximum magnitudes of n_1 and n_2). With the registers in this form, we can simply use the same procedure employed for the single-particle phases. Once the desired phases are applied, we perform the reverse of the computation Eqn. (S19) and then repeat the whole process with another set of particle pairings. For a system of P particles, the total number of particle pairings is $\frac{1}{2}P(P-1)$. However as we discuss in the Results section of the main text, there is a natural parallelism so that the time cost should scale only linearly with P. Moreover, there are methods to trade greater hardware resources to reduce the time cost; Jones *et al.* introduced a parallelised scheme for speeding up the computation of the particle pairings using only $O(\log P)$, or even $O(1)$, circuit depths [7]. Regardless of the implementation method used, the elements of the diagonal matrix D_{int} are thus simply sums of phases.

In the approach just described, we are finding the relative coordinates of each particle pairing. There was no need to compute the complete transformation by mapping the second particle's register to $|n_1 + n_2, m_1 + m_2, l_1 + l_2\rangle$. However, this would have been possible. A motivation for completing this full transformation is that general, non-diagonal operations could be performed if we wished; this observation relates to the augmented split-operator concept developed presently.

S3. NUMERICAL MODELLING

Here we describe further details of the hardware, software, and configuration details relating to the numerical simulations presented in the main paper.

A. Emulation software and hardware

All numerical results in this manuscript were ultimately obtained through the Quantum Exact Simulation Toolkit (QuEST) [32], though interfaced through the `Python` pyQuEST [34] and `Mathematica` QuESTlink [33] software packages. The core QuEST simulator is written in `C` behind a hardware agnostic interface, allowing redeployment of the simulations in this manuscript between laptops, GPUs and distributed supercomputing facilities. This enabled multi-threaded and GPU simulation of the modestly sized systems, such as the 25-qubit 2D Hydrogen scattering presented, directly within QuESTlink. While open-source, we note that the use of QuESTlink requires a Wolfram Engine environment, obtainable either through the commercial Mathematica product, or the recently released Wolfram Engine standalone [105].

Results for the largest scale systems we considered were generated with pyQuEST, which enables relatively low-level access to QuEST simulation primitives through a high-level `Python` interface. Importantly, pyQuEST inherits the capacity to run distributed tasks, where the numerical representation of a quantum state is partitioned between compute nodes cooperating over a network. This allows both the representation of states too large to fit into the memory of any single compute node, and their concurrent simulation – each multicore node is further able to parallelise its local simulation tasks through multithreading. In this manuscript, distributed pyQuEST was used to emulate 36-qubit quantum computers in our study of 3D helium, employing up to 32 compute nodes of the Oxford Advanced Research Computing (ARC) facility [80]. Each node contains 48 CPU cores, and took roughly 52 hours (≈ 50 000 core hours) to process its 64 GiB partition of the full 1 TiB quantum state-vector.

B. Emulation of SO-QFT

The QuEST family of emulators perform numerical simulation at the level of individual gates. Supported gates include all commonly used operations, and general unitaries of any number of control and target qubits, and therefore all operations required of the quantum algorithms presented in this manuscript. However, completing this work required the repeated simulation of circuits within which a series of contiguous gates could be more efficiently effected by a single invocation of a bespoke function. We implemented this optimisation to accelerate the simulations in this manuscript, which has since been integrated into the QuEST emulators. The interested reader may view these documented facilities here. We now describe these optimisations, and where they were invoked, in detail.

In the Methods section in the main text, we described how our grid-based description of 3D multi-electron systems meant that particle-field and particle-particle interactions admit a unitary time evolution operator which can be split into step

operators

$$U_{\rm U}\,|n\,m\,l\rangle = \exp\left(-i\frac{Q\,\delta t}{\sqrt{n^2+m^2+l^2}}\right)|n\,m\,l\rangle\,, \qquad \text{(S20)}$$

$$U_{\rm V}\,|n_1 m_1 l_1\rangle\,|n_2 m_2 l_2\rangle = \exp(-i\Theta)\,|n_1 m_1 l_1\rangle\,|n_2 m_2 l_2\rangle\,,$$

$$\Theta = \frac{2^{1-n_r}Q\delta t}{\sqrt{(n_1-n_2)^2+(m_1-m_2)^2+(l_1-l_2)^2}},$$

where sub-register $|n\,m\,l\rangle$ encode (z,y,x) coordinates of a single particles, which may be negative (encoded with two's complement binary). An experimentalist must effect these $(d\,n_r)$- and $(2\,d\,n_r)$-qubit operators through $\mathcal{O}({n_r}^2)$ single-qubit and double-qubit phase gates. However, we can leverage that $U_{\rm U}$ and $U_{\rm V}$ are diagonal in the real space representation to numerically simulate them cheaper than even a *single* general unitary gate, in an *embarrassingly parallel* manner. We formally present such a scheme to effect $U_{\rm U}$, the simplest of the split-operators, upon a distributed statevector in Algorithm 1. For clarity, we have excluded the pseudocode for additional functionality critical to our actual implementation, such as the ability to override the phases at particular l, m, n values to avoid phase divergences.

Algorithm 1: Embarassingly parallel distributed simulation of the 3D $U_{\rm U}$ split-operator upon an N-qubit statevector $|\psi\rangle$, which is uniformly distributed between 2^k nodes. $\vec{\psi}$ is the 2^{N-k} vector of complex amplitudes stored in each node, with i-th element $\vec{\psi}[i]$, indexed from 0. Each node has a unique rank $0 \le r < 2^k$. Integers q_l, q_m, q_n are the starting qubit indices of the contiguous n_r-qubit registers which together form substate $|n\rangle\,|m\rangle\,|l\rangle$ of Equation S20, via a two's complement signed binary encoding. Symbols & and $\gg$ notate bit-wise AND and bit right-shift operators respectively. The outer **for** loop of our algorithm is trivially parallelised using multithreading or GPU acceleration.

apply$\boldsymbol{U}_{\rm U}$($\vec{\psi}$, Q, δt, q_l, q_m, q_n, n_r)
  // iterate every local basis state
  for i **in** $\{0,\ \dots,\ 2^{N-k}-1\}$
    // determine global index of basis state
    $j = r\,2^{N-k}+i$
    // determine sub-register values of basis state
    l = **getRegVal**(j, q_l, n_r)
    m = **getRegVal**(j, q_m, n_r)
    n = **getRegVal**(j, q_n, n_r)
    // evaluate the phase
    $\theta = Q\ \delta t\ /\sqrt{l^2+m^2+n^2}$
    // modify the amplitude
    $\vec{\psi}[i] = \exp(-i\,\theta)\ \vec{\psi}[i]$

// returns $(b \ge 0)$-th bit of integer j
getBit(j, b)
  return $(j \gg b)$ & 1

// returns the signed value of the n_r contiguous qubits, starting at index q, in the j-th basis state
getRegVal(j, q, n_r)
  $v = 0$
  for k **in** $\{0,\ \dots,\ n_r-2\}$
    $v = v + 2^k$ **getBit**(j, $q+k$)
  if **getBit**(j, $q+n_r-1$) **is** 1
    $v = v - 2^{\,n_r-1}$
  return v

Use of Algorithm 1 and several similar strategies for the other split-operators was crucial in order to numerically study the various simulation scenarios presented in the main paper without incurring impractical time costs.

We stress that the use of these high level functions, which do not involve specifying a circuit-level description, does not compromise the exact nature of our numerical emulation of a quantum processor. It is simply an expediency allowing us to arrive at that the exact behaviour with a more modest use of classical resources that would be required if we were to explicitly use gate-level operations throughout. In the real device, such circuit-level prescriptions must of course be used.

For the $U_{\rm kin}$ operator, which simply applies a phase dependent on the square of the binary number k in each of the system's sub-registers, this is straightforward: we require only a series of single- and two-qubit phase gates applied directly to the sub-registers [8, 10]. Of $O(n_r^2)$ such gates would be required for each sub-register of n_r qubits.

The implementation of $U_{\rm U}$ and $U_{\rm V}$ is more complex as it involves the Coulomb potential $1/|\mathbf{r}|$. In the main paper, we describe the arithmetic that occurs in the case that we are applying $U_{\rm V}$ and therefore require sub-registers to represent $x_i - x_j$ etc. One could then apply a circuit C to compute the desired phase $1/r$ into an ancilla register (i.e. a binary representation of the desired phase to any given accuracy), followed by actually implementing that phase by a series of single-qubit phase gates applied to the ancilla qubits. Finally one would apply circuit $C^\dagger$ to un-compute the ancilla's state, disentangling it from the main registers.

Any classical algorithm that computes $1/\sqrt{x^2+y^2+z^2}$ from inputs x, y, z will in principle suffice; such an algorithm might be expressed in terms of irreversible operations (AND, OR, XOR etc) but any such circuit be be re-expressed as classical reversible circuit and thus provide a suitable C. It is of course interesting to optimise this, especially since quantum gate sets are can potentially implement classically-reversible circuits more efficiently and we will wish to be as frugal as possible with the use of ancilla qubits. However even an inefficient implementation would suffice in the sense that it would not alter the overall scaling of the SO-QFT method: the computation of $1/r$ happens at the register level, and register size scales only very weakly with the complexity of the simulated system as argued in the main text.

S4. MULTI-QUBIT PHASE ESTIMATION AND FOURIER ANALYSIS

In order that the present paper can be a useful resource for introducing the grid-based method, we now summarise the standard multi-qubit phase estimation method using the present paper's notation. This method is of course more powerful than the frugal single-ancilla technique described in the main paper.

The logarithmically-scaling phase estimation of Kitaev [67], trades higher qubit overhead for measuring the phase with far

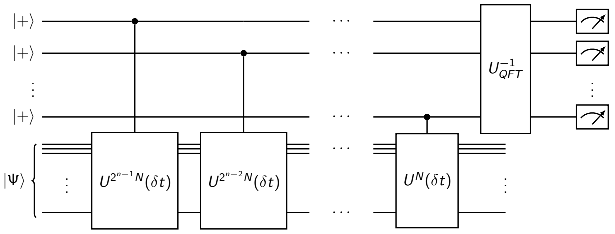

FIG. S2. Generalisation of the single-ancilla IPE to the 'canonical' phase estimation using an n-qubit ancilla register. The ith most significant ancilla qubit controls the propagation of $2^{n-i}N$ split-operator steps in the main register.

less sampling; in suitable cases, even in a single shot. Consider an ancillary register with S qubits and a working register containing the state of interest. We first conditionally apply $2^{S-1}N$ split-operator steps to the state, controlled by the most significant ancillary qubit. We then repeat by conditionally applying $2^{S-2}N$ split-operator cycles, now controlled by the next most significant ancillary qubit, and repeat again until every ancillary qubit has been used and the least significant qubit controls only N split-operator steps. At this point the quantum computer is in the state

$$\frac{1}{\sqrt{2^S}}\sum_{m=0}^{2^S-1}|m\rangle\, U^{mN}(\delta t)\,|\Psi\rangle\,. \tag{S21}$$

If $|\Psi\rangle$ is an eigenstate $|\Psi_n\rangle$ with energy E_n, the state is thus

$$\begin{aligned}&\frac{1}{\sqrt{2^S}}\,|\Psi_n\rangle\sum_{m=0}^{2^S-1}e^{-iE_n mN\delta t}\,|m\rangle\\ =&\frac{1}{\sqrt{2^S}}\,|\Psi_n\rangle\sum_{m=0}^{2^S-1}e^{-i\phi m}\,|m\rangle\end{aligned} \tag{S22}$$

where $\phi = E_n N\delta t$. The phase information ϕ is now encoded in the ancillary qubit register, and can be efficiently extracted using an inverse QFT on the ancillary register. Indeed, if $\phi = 2^k$ where $k \in \mathbb{N}$, then a QFT of the ancillary register should yield the binary representation of k upon measurement.

We also mention that classical Fourier analysis of the autocorrelation signal can also reveal the power spectrum, with resonance peaks that correspond to the stationary states of the system. This is in many ways the classical analogue of the canonical phase estimation method. Specifically, the power spectrum is peaked at the eigenvalues of $\hat{H}_{\text{tot}}$ with heights corresponding to $|c_n|^2$

$$\frac{1}{\sqrt{2\pi}}\int\langle\Psi(t)|\Psi(0)\rangle\, e^{i\omega t}dt = \sqrt{2\pi}\sum_n |c_n|^2\delta(\omega - E_n) \tag{S23}$$

In practice, the discrete and finite time integration limits the resolution of the power spectrum, given by

$$\delta\omega = \frac{2\pi}{M_{\text{tot}}N\delta t} \tag{S24}$$

where M_{tot} is the total number of time samples.

S5. STATE PREPARATION

A. State loading

As discussed in the main paper, our use of emulated quantum computers means that we can side-step the challenge of preparing a known, analytic initial state on our device – we simply load it into the emulator's memory. For completeness, we briefly review some of the literature that describes methods and costs for real quantum processors.

In 2009, Ward *et al.* [31] extended the work of Zalka [4] to develop such a method for generating physically relevant states in grid representations, which we summarise here. We begin with the task of loading suitable initial states into the particle quantum register. The objective is to distribute a single particle state ϕ centred about (x_0, y_0, z_0) within a simulation box of width L across the amplitudes of an N-qubit register

$$|0\rangle\,|0\rangle\,|0\rangle \Rightarrow \phi\,(\mathbf{r})\,|n\rangle\,|l\rangle\,|m\rangle \tag{S25}$$

where $\mathbf{r} = (n\delta r - x_0, l\delta r - y_0, m\delta r - z_0)$ and $\delta r = L/2^{n_r}$. L must be sufficiently large to capture the significant parts of the wavefuntion and the sum of all the amplitudes squared should be unity. In 1998, Zalka proposed a constructive algorithm for loading single particle distributions onto a register [4], which was subsequently either rediscovered or generalised, most notably by Grover and Rudolph in 2002 [106], in a number of contexts [107–110]. The method splits the norm of the single particle function N times across the N qubits by performing qubit rotations, the angles for which are determined by computing exponentially many integrals. Complex-valued functions will require additional procedures for including the phase in each amplitude. There are conflicting opinions on the efficiency of the method; some argue that the method has an exponential $O(2^N)$ overhead [109, 111], while others claim that it can be efficient [112], perhaps using quantum computers to solve the integrals [31]. In recent years, more novel approaches for initial state loading techniques have been proposed. Ollitrault *et al.* prepared Gaussian amplitudes using hybrid variational algorithms [10]. Holmes and Matsura used matrix product states to represent functions, which maps to state preparation circuits with linear-depth [111]. In particular, a noise-resistant technique for efficient preparation of distributions from Rattew *et al.* [113, 114] shows promise for generalisation to the single particle functions required.

B. Alternative antisymmetrisation method

In the main paper we described, and numerically tested, a means for antisymmetrising a state; the input to the procedure is simply as a tensor product of P unique single particle states represented by our P registers.

We now outline the second approach, also exploiting H_{synth}. Instead of fully representing all the tag registers, we can consider what happens if we compute and uncompute the energies of the states $|\psi\rangle$ 'on the fly'. We assume that we have available a sorting network formed of swap operations and 'controller qubits' that determine whether each swap operation will occur. Moreover we take it that these controller qubits are already properly primed in the appropriate superposition to create an antisymmetric permutation (as per the method of Berry *et al.* [41]). As we push our ini-

tially canonically-ordered state 'backwards' through this network, as per Berry, we must erase each controller qubit as we go: wherever two inputs $|A\rangle\,|B\rangle$ have indeed been swapped to $|B\rangle\,|A\rangle$, we must now flip the controller qubit from $|1\rangle$ to $|0\rangle$.

Ref. [41] describes this erasure for the case of integer labels, noting that if $|A\rangle$ and $|B\rangle$ are any two superpositions of integers, but all integers in $|A\rangle$ are smaller than all those in $|B\rangle$, then the reordering implied by the swap can be used to erase the controller. We must instead erase the controller qubit with an analogous condition based on the energies of our grid-based states. This can be achieved by introducing a set of t ancilla qubits in the state $|+\rangle^{\otimes t}$ and performing a phase estimation process, where the two registers involved in the swap operation are subject to controlled-$e^{i\,H_{synth}C\pi}$ and controlled-$e^{-i\,H_{synth}C\pi}$, respectively. Here, as earlier, C denotes the appropriate power of two for each ancilla qubit. As a result, the ancilla now encodes the QFT of a number that is either positive or negative; by performing an inverse-QFT, that sign is made manifest as the most-significant bit, and can be used to unset the swap controller qubit before the entire computation is reversed.

This latter approach has the merit that not all tag registers need to coexist simultaneously; the process can occur sequentially using only t rather than $P\,t$ qubits (albeit incurring a time cost). However the method is more computationally costly, requiring more phase-estimation processes: multiple uses to compute and uncompute at *each* swap in the sorting network. An interesting distinction is that there will be more validating measurements, one confirming each controller qubit is in state $|0\rangle$ after its swap process is complete. It would be interesting to evaluate the overall pros and cons of the two approaches in a further study.

C. Driving loaded state to desired state

Loading multiple uncorrelated single particle states onto separate quantum registers is not sufficient to represent many-particle eigenstates of real molecular Hamiltonians. As noted in the main text, a popular method is to drive an initial state that can be efficiently loaded onto a quantum computer towards the correlated molecular ground state. An advantage of this approach is that we need not have the ability to analytically describe the target state. Techniques include adiabatic evolution [115], projective eigenstate filtering [116], phase estimation [41, 67, 71], and imaginary time evolution [12, 72]. In this work we demonstrate a small subset of these methods that only require a single ancilla qubit, targetting early fault-tolerant quantum computers; these included a probabilistic variant of imaginary time evolution, and a protective IPE method. Recent advances in eigenstate filtering and quantum signal processing using only a single ancilla [117] can potentially be applicable to preparing ground states of grid-based states.

An alternative proposed by Ward *et al.* [31] is to load occupied Hartree-Fock orbitals [70] into real-space particle registers using the aforementioned method by Zalka, followed by (anti)symmetrisation, to generate Slater-determinant type states. They further generalise this to preparing superpositions of Slater-determinant type states in the real-space particle registers, yielding exact molecular ground states through the creation of Full Configuration Interaction (FCI) states. Their method however demands the classical or quantum precomputation of the electronic structure in second-quantisation, loading the result in ancilla qubits, and achieving a transformation which loads the information from the second-quantised ancilla to the real-space particle registers. The authors did not detail how such a transformation can be achieved, and loading a factorial number of Slater determinants from the FCI state may pose challenges.

For completeness, we also discuss the general approach for using the IPE for state preparation. Assuming we know the energy E of the state we wish to prepare, we apply controlled evolution as before, but choose a total time $T = N\delta t = 2n\pi/E$ for some integer n, to measure the ancilla. If the initial state had been exactly the desired state then the ancilla would now certainly be found in the $|+\rangle$. An initial state with a different energy $E_k \neq E$ would in general not yield the $|+\rangle$ with certainty but rather with probability $\cos^2(TE_k)$. We therefore demand a $|+\rangle$ outcome, and restart if we do not see it. On success, the post-selected state on the main register has a boosted amplitude associated with the desired state because other states have been suppressed by the factor $\sin^2(TE_k)$, analogous to PITE. We repeat the process to probabilistically purify the desired state (or if degeneracy is present, a state in the degenerate subspace with energy E). Our probability of success is precisely the total probability associated with the target state's component of the initial state, therefore it is important to prepare an initial state having reasonable overlap with the target. The total time required will depend on the inverse of the energy gap $E_k - E$ between the desired state and the nearest unwanted component.

S6. THE COULOMB POTENTIAL: DEMANDS ON SPATIAL AND TEMPORAL RESOLUTION

The standard split-operator and indeed the SO-QFT method involves three critical approximations: (a) a discretised representation of the state, (b) propagation of the potential phase assuming the interaction operator $\hat{H}_{\rm int}$ is exactly diagonal, and (c) the Trotter error from discretisation of $e^{-i\hat{H}_{\rm tot}\delta t}$ into the two non-commuting $e^{-i\hat{H}_{\rm kin}\delta t}$ and $e^{-i\hat{H}_{\rm int}\delta t}$ phases; see Ref. [13]. Both (a) and (b) become exact in the limit of infinite spatial resolution, while (c) becomes exact in the limit of infinitesimally small time steps.

It is worth stressing that approximation (a) has consequences for both the wavefunction representation and moreover for the operators forming the SO-QFT cycle: the representation of these continuous operators as a discrete matrix is an approximation, and moreover the nature of the off-diagonal terms in the potential part will depend on the resolution. The severity of the impact depends on the modelled interactions; if we were using quadratic potentials $V(\mathbf{r}) \propto r^2$, then a modest resolution could ensure that the potential is almost constant over interval between one spatial pixel function and the next, $x_{n+1} - x_n = \delta r$. We remark that the preliminary testing and debugging of our emulation code was performed on 'easy' scenarios of this kind.

However, the results presented in this paper concern the far more challenging Coulomb potentials which are singular at $\mathbf{r} = \mathbf{0}$, where the potential changes rapidly between neighbouring pixel functions. In this case, a relatively modest resolution can still suffice if the wavefunctions involved have near-zero amplitude at the origin; for example in 2D hydrogen, states with quantum number $m \neq 0$ have this prop-

erty, and this motivates our use of such states in several of the numerical studies presented in the main paper. Nevertheless, many important states (for example the ground state of atomic electrons) have significant amplitudes at the origin. It is well-established in the literature that any reasonable observable property of our system will converge properly as the spatial resolution δr^{-1} is increased (provided that other parameters are suitably updated; notably δt must decrease as explained below). This may not be intuitively obvious since increasing the resolution introduces new pixel functions that are closer to the origin and for whom the diagonal approximation is 'worse'. The most direct way to see this is by considering the unitary nature of the simulation; the SO-QFT method, while approximate, is nevertheless strictly unitary. As the resolution increases, the pixels that are closest to the Coulomb singularity may indeed be more imperfectly updated by our simulation steps, but they constitute an exponentially vanishing component of the entire simulation, both in terms of the Hilbert space and (crucially) the total amplitude associated with them. Thus it ultimately becomes impossible for any observable to distinguish between a state evolved under the ideal $e^{-i\hat{H}_{\rm tot}\delta t}$ and the same state evolved using SO-QFT at high resolution.

We can therefore be confident that there is *some* finite resolution δr that will suffice to adequately approximate the Coulomb operator, addressing the first two sources of error. But this comes with an important caveat that relates to the Trotter error. The problematic cost is not the qubit count but rather the time required for our simulation; as δr decreases we must reduce the time step δt proportionately and thus more time steps will be needed to simulate a given period of dynamics. The intuitive reason is clear: the effect of the kinetic operator is to change the shape of the wavepacket in real space, but if δt is too large, the change is significant on the scale of multiple pixel widths δr. Amplitude can then pass through otherwise impermeable features such as the Coulomb singularity. Instead, δt should be small enough that the kinetic part of the SO-QFT step only modestly alters the amplitudes associated with each pixel function.

One means of optimising the approach would be selecting an appropriate Trotter sequence [64, 65, 82]. In this paper we use only the simplest 1$^{\rm st}$ order sequence where the error $\epsilon = \frac{1}{2}[\hat{H}_{\rm kin}, \hat{H}_{\rm int}]\delta t^2$. Any order Trotter sequence will ultimately have an error involving on the same commutator (in nested form), which in turn depends on the higher order derivatives of the interaction potentials. In our finite discretisation of the Coulomb potential, it will again be elements very close to the singularity, where the derivatives are the largest, that are the most problematic, and the more so as the spatial resolution increases. Thus we anticipate that δt should still commensurately decrease, albeit its severity will vary with the Trotter order. Ultimately this cost will not prevent successful simulation of interesting molecules on a quantum computer; as discussed in the main text, the proper choice of δr^{-1} does not scale with system size but only with the highest nuclear charge. Nevertheless it is an impactful cost.

We conclude this Section by noting that methods for handling the Coulomb singularity in real-space have been explored in the literature. One could cap or truncate the potential [13, 118–120], or describe it with a Fourier expansion so that it cannot be more highly curved than the highest Fourier component (which might be chosen to match the highest k plane waves in the state's representation) [99, 121]. The nuclear potential could also be replaced with an effective potential representing which accounts for the inner shell electrons [122]. Methods of this kind may ultimately prove to be the optimal approach for quantum computer enabled simulations too. For the present paper we attempt to deal with the Coulomb potential in its 'natural' form by effectively capping it; we always define the singular origin of the potential between two grid points throughout this work.

S7. PLACEMENT OF NUCLEAR ORIGIN

We briefly report that placement of the nuclear origin between grid points does not significantly affect the dynamics (summarised in Table S1). When we time propagate the $\Psi_{1,1}$ state with the Coulomb potential centred at different fractions between two neighbouring grid points, the state visibly oscillates where grid points lie asymmetrically around the Coulomb origin. However, the magnitude of the loss in fidelity is limited. Most importantly, the energy from the phase estimation is not influenced at all by the skew. We conclude that discrepancies in the placement of grid points relative to the system can, if needed, be alleviated with a brute force increase in the spatial resolution and correspondingly the time resolution, but its influence on observables related to state fidelity can be negligible.

	0.5	0.4	0.3	0.2	0.1
Max loss	7×10^{-7}	0.00085	0.00021	0.0042	0.0022
ϵ ($10^{-5}E_h$)	-1.84	-1.11	0.29	-1.66	5.98

TABLE S1. Placement of the Coulomb nuclear potential between grid points and its influence on the autocorrelation as well as the energy predicted through phase estimation.

S8. RESOURCE SCALING

In this work we considered the way in which the grid-based method's resources can be expected to scale. We noted that there are detailed studies in the literature. The contribution to this topic made by the present paper is that its emulated algorithms may elucidate some of the constants that arise in any scaling analysis.

A. Space Cost

We argued that the qubit count should scale with particle count P according to $3n_rP$ with

$$n_r \approx C_3 + \log_2(Z_{\rm max}) + \frac{1}{3}\log_2(P) \qquad \text{(S26)}$$

(reiterating from main text) where $Z_{\rm max}$ accounts for the highest nuclear charge in the modelled system. We noted that this crude expression does not account for the fact that a given atom's radius has a sub-linear dependence on the number of electrons; accounting for this would lower the outcome.

Since n_r really depends on more properties of the problem besides $Z_{\rm max}$ and P, C_3 will not be an absolute constant,

but rather fluctuate with other system properties, such as the geometry and electron configuration. We can proceed to estimate the required resources for the two molecules we identified as interesting in the Introduction to arrive at two example values for C_3. These will then give us a rough idea of its range.

We begin by noting that in the numerical modelling reported here, remarkably accurate and stable simulations can be achieved with as few as 6 qubits per x, y or z sub-register (thus, 18 qubits per 3D particle). Methods such as iterative phase estimation, requiring only one additional qubit, can then obtain eigenenergies with accuracy up to 6 decimal places. Our results using the ASO method suggest that even core-peaked electronic states can be modelled with only a modest increase in resolution. For present purposes we therefore take an optimistic stance and assume that $n_r = 7$ can suffice for systems with $P = Z = 1$. For larger values of P and Z, more data points per dimension are necessary to compensate for differently sized simulation boxes and higher curvature of core states. We discuss momentarily how we estimate these required changes.

For molecules that are already well-understood, the ionization potential can be used to estimate their long-range behaviour [123], however one may wish to use a quantum simulator to explore molecular systems that have not been experimentally charatcterised. Therefore we will adopt a first-principles argument based only on the constituent atoms and their presumed locations. We place the centre of the highest occupied hydrogen-like wave function of each atom at the coordinates of each nucleus, and calculate its radial charge density according to Ref. [124], which also semi-empirically accounts for nucleus shielding. Summing up the contributions from all atoms gives us an approximation to the total charge density if the electrons of different atoms were not interacting with each other. We then find the maximum of this total density on the surface of the simulation box, which informs us about how strongly the electrons will interact with its boundary. For molecules of interest here, the atomic locations in their equilibrium geometry are presented in Ref. [125].

To get an idea of what an acceptable charge density at the box surface could be, we utilise the calculation of 3D helium without electron-electron interaction discussed in the main text. From the electron configuration and box size of that simulation, we derive its maximum surface electron density ρ_0 using the approximate method described above. Consequently, the simulation box for any other molecule might be considered sufficiently large whenever the method above yields a maximum surface electron density that does not exceed ρ_0. Taking a relatively optimistic line in our resource estimation, we will refer to this rule; but note that because it is only a rough approximation to the extent of the electron cloud, and specific scenarios might necessitate an increase. Interesting dynamics with substantial numbers of moving particles may require significantly larger boxes, as to not let significant amplitude collide with the simulation boundary (or to allow an adequate attenuation regions as described in the main paper).

Besides varying simulation box sizes, we should also account for changes in the required spatial resolution. For $Z > 1$, the features of hydrogenic wave functions shrink by a factor of exactly Z^{-1}, which is usually also a good approximation for low-lying core states. To accurately resolve these electronic states, we must therefore increase the number of grid points per unit length by a factor of $Z_{\max}$.

For Ammonia (NH_3), the more modest of the two molecules we identified as interesting in the Introduction, an optimistic resource audit can proceed as follows: The highest charge in the molecule is $Z_{\max} = 7$. Therefore, the number of grid points must increase to 7 times that of our estimate for $Z = 1$. At the same time, the above mentioned method to determine the box size yields a side length of ≈ 1.1 times the length required for a single hydrogen atom, giving a total factor of $\approx 1.1 \times 7 = 7.7 < 8 = 2^3$, which means we must increase n_r by 3 from its reference value of 7, giving $n_r = 10$ qubits per particle and dimension. Substituting these values in Eq. S26 and rounding where appropriate leads to a value of $C_3^{\mathrm{NH_3}} \approx 6$. For 14 particles (10 electrons and 4 nuclei) in 3 dimensions, we arrive at a total of $3 \times 10 \times 14 = 420$ qubits. We might round this to 450, recognising that multiple ancillas may be required even in a very frugal implementation.

The more challenging case we mentioned was C_2F_6. The highest-charge nuclei are those of fluorine, giving us $Z_{\max} = 9$, thus increasing the required spatial resolution 9-fold. Surprisingly, as the electron densities of C and F drop off quite rapidly with increasing distance from the atom, the above described method suggests that the required side length of the simulation box could be as small as 0.85 times that of a single hydrogen atom. This gives a total factor of $\approx 0.85 \times 9 = 7.65 < 8 = 2^3$, again meaning that 3 qubits must be added to the reference value, giving a total of $n_r = 10$. Using Eq. S26 again, we arrive at $C_3^{\mathrm{C_2F_6}} \approx 5$. The total number of required qubits is $3 \times 10 \times 74 = 2220$, which we might round up to 2250 to allow for a frugal ancilla overhead.

From these two data points, we cautiously estimate C_3 to be on the order of ~ 10, but note that it might be lower for advantageous circumstances as in the examples given, or may also increase in unfavourable situations.

B. Time cost

It is difficult to provide meaningful estimate of total 'wall clock' time for a significant quantum simulation, even to within an order of magnitude. We will nonetheless use some broad assumptions to provide generally indicative numbers for the time cost.

The timescale of the simulation depends heavily on the energy scales across which the events probed occur; the models in the paper (electron scattering, ionisation) correspond to high energy 'fast events' that occur on a sub-femtosecond timescale, whereas resulting dynamics (e.g. bond breaking, molecular fragmentation, energy transfer) are 'slow events' that happen at $O(1)$ even up to $O(10)$ picosecond timescales. We postulate this to be independent of system size. The resolution by which we discretise this total simulation length by δt depends only on the δr sufficient to describe the most tightly bound electrons of the element with highest nuclear charge, which also does not change with system size. Moreover, the methods explored here, and indeed proposals of e.g. Ref. [116], indicate that energy estimation and state preparation could also be achieved using a number of time steps that does not scale significantly with system size.

The time to complete each SO-QFT cycle does however scale with system size. There is a modest scaling due to the computation associated with introducing proper phases (equivalent to binary addition/subtraction, squaring, and inversion) which scales with the number of qubits in each register – although not, of course, with the number of qubits in

total. Since the n_r scales only logarithmically with simulation box size L, this time factor should be poly-logarithmic in e.g. the number of particles $O(\log^k P)$. A more significant factor comes from the need to compute the electron-electron interaction for all possible pairings of electrons. The task can be divided into a series stages within each of which all electrons are paired-off and we can assume that computations relevant to electron-electron dynamics occur in parallel (see [7]). The number of such stages is $P-1$ for P electrons, so that the total time for a complete SO-QFT implementation is of the order $O(P \log^k P)$. Considerations such as the time required to move from one pairing to another should also be borne in mind, but one finds that this should not scale with n even in devices with restricted connectivity.

Our QFT implementation used an explicit quantum circuit, while the conditional phases were applied directly using a bespoke algorithm (see Section S3); if we focus on the QFT component and use the same implementation employed in our emulator (Fig S1) then $O(n_r^2)$ gates are required. A full SO-QFT cycle may involve operations equivalent to several QFTs, since they constitute an approach to the addition and squaring operations that we employ. Moreover other operations may require n_r^2 gates, such as the application of the $\propto k^2$ phases for the kinetic operator. If we therefore take $10n_r^2$ as a plausible suggestion for the gate cost of each SO-QFT sub-step in a parallelised implementation, then for the C_2F_6 molecule for each SO-QFT cycle we have a total gate depth of $260 \times 10 \times 10^2$ which we conservatively call $O(10^6)$ (and $260/2 = 130$ such processes occurring in parallel throughout that cycle).

Many of the simulations presented in the main paper used $O(10^3)$ SO-QFT cycles to generate dynamics with good precision. This count would diminish with the adoption of more sophisticated Trotter sequences than the lowest-order pattern employed in the paper; one might expect an order of magnitude reduction is possible here. Thus, given the conclusion of the above paragraph that $O(10^6)$ gates are required for each SO-QFT cycle, one could assume that understanding a 'fast event' such as electron ionisation requires an algorithmic gate depth of hundreds of millions. In the main paper we indicate how such numbers may translate to clock time, finding one execution of the algorithm requires only minutes.

However, we might ask whether it is possible to encapsulate a 'slow event' like the subsequent dynamics of a molecule immediately after an interesting 'fast event'. Simplistically, this might seem to imply the need for a corresponding multiplicative factor in the number of SO-QFT cycles required for the complete simulation. Fortunately however, as we explored in our two-particle scattering simulation, it might be natural to use a coarser time stepping according to the speed of the physics that is unfolding. Accounting for these various considerations, we assume that $O(10^5)$ SO-QFT cycles should suffice to reveal desired 'slow', longer time quantum molecular dynamics. One concludes that the gate depth of the parallelised quantum algorithm for evolving such a system in the picosecond timescale is about a hundred billion. As noted in the main paper, this leads to a total clock time of the order of a day for this more challenging simulation.

REFERENCES AND NOTES

1. S. Lee, J. Lee, H. Zhai, Y. Tong, A. M. Dalzell, A. Kumar, P. Helms, J. Gray, Z.-H. Cui, W. Liu, M. Kastoryano, R. Babbush, J. Preskill, D. R. Reichman, E. T. Campbell, E. F. Valeev, L. Lin, G. K.-L. Chan, Is there evidence for exponential quantum advantage in quantum chemistry? arxiv:2208.02199 [physics.chem-ph] (3 August 2022).
2. F. Arute, K. Arya, R. Babbush, D. Bacon, J. C. Bardin, R. Barends, R. Biswas, S. Boixo, F. G. S. L. Brandao, D. A. Buell, B. Burkett, Y. Chen, Z. Chen, B. Chiaro, R. Collins, W. Courtney, A. Dunsworth, E. Farhi, B. Foxen, A. Fowler, C. Gidney, M. Giustina, R. Graff, K. Guerin, S. Habegger, M. P. Harrigan, M. J. Hartmann, A. Ho, M. Hoffmann, T. Huang, T. S. Humble, S. V. Isakov, E. Jeffrey, Z. Jiang, D. Kafri, K. Kechedzhi, J. Kelly, P. V. Klimov, S. Knysh, A. Korotkov, F. Kostritsa, D. Landhuis, M. Lindmark, E. Lucero, D. Lyakh, S. Mandrà, J. R. McClean, M. M. Ewen, A. Megrant, X. Mi, K. Michielsen, M. Mohseni, J. Mutus, O. Naaman, M. Neeley, C. Neill, M. Y. Niu, E. Ostby, A. Petukhov, J. C. Platt, C. Quintana, E. G. Rieffel, P. Roushan, N. C. Rubin, D. Sank, K. J. Satzinger, V. Smelyanskiy, K. J. Sung, M. D. Trevithick, A. Vainsencher, B. Villalonga, T. White, Z. J. Yao, P. Yeh, A. Zalcman, H. Neven, J. M. Martinis, Quantum supremacy using a programmable superconducting processor. *Nature* **574**, 505–510 (2019).
3. S. Wiesner, Simulations of many-body quantum systems by a quantum computer. arXiv:quant-ph/9603028 (quant-ph) (26 March 1996).
4. C. Zalka, Simulating quantum systems on a quantum computer. *Proc. R. Soc. Lond. A Math. Phys. Eng. Sci.* **454**, 313–322 (1998).
5. I. Kassal, S. P. Jordan, P. J. Love, M. Mohseni, A. Aspuru-Guzik, Polynomial-time quantum algorithm for the simulation of chemical dynamics. *Proc. Natl. Acad. Sci. U.S.A.* **105**, 18681–18686 (2008).
6. G. Benenti, G. Strini, Quantum simulation of the single-particle Schrödinger equation. *Am. J. Phys.* **76**, 657–662 (2008).
7. N. C. Jones, J. D. Whitfield, P. L. McMahon, M.-H. Yung, R. V. Meter, A. Aspuru-Guzik, Y. Yamamoto, Faster quantum chemistry simulation on fault-tolerant quantum computers. *New J. Phys.* **14**, 115023 (2012).
8. R. D. Somma, Quantum simulations of one dimensional quantum systems. arXiv:1503.06319 [quant-ph] (21 March 2016).
9. I. D. Kivlichan, N. Wiebe, R. Babbush, A. Aspuru-Guzik, Bounding the costs of quantum simulation of many-body physics in real space *J. Phys. A Math. Theor.* **50**, 305301 (2017).

10. P. J. Ollitrault, G. Mazzola, I. Tavernelli, Nonadiabatic molecular quantum dynamics with quantum computers. *Phys. Rev. Lett.* **125**, 260511 (2020).
11. Y. Su, D. W. Berry, N. Wiebe, N. Rubin, R. Babbush, Fault-tolerant quantum simulations of chemistry in first quantization. *PRX Quantum* **2**, 040332 (2021).
12. T. Kosugi, Y. Nishiya, H. Nishi, Y.-i. Matsushita, Imaginary-time evolution using forward and backward real-time evolution with a single ancilla: First-quantized eigensolver algorithm for quantum chemistry. *Phys. Rev. Res.* **4**, 033121 (2022).
13. A. M. Childs, J. Leng, T. Li, J.-P. Liu, C. Zhang, Quantum simulation of real-space dynamics. arXiv:2203.17006 [quant-ph] (31 March 2022).
14. B. Poirier, J. Jerke, Full-dimensional Schrödinger wavefunction calculations using tensors and quantum computers: The Cartesian component-separated approach. *Phys. Chem. Chem. Phys.* **24**, 4437–4454 (2022).
15. P. J. Ollitrault, S. Jandura, A. Miessen, I. Burghardt, R. Martinazzo, F. Tacchino, I. Tavernelli, Quantum algorithms for grid-based variational time evolution. arXiv:2203.02521 [quant-ph] (4 March 2022).
16. T. Kosugi, H. Nishi, Y. Matsushita, Exhaustive search for optimal molecular geometries using imaginary-time evolution on a quantum computer. arXiv:2210.09883 [quant-ph] (18 October 2022).
17. H. Hirai, T. Horiba, S. Shirai, K. Kanno, K. Omiya, Y. O. Nakagawa, S. Koh, Molecular structure optimization based on electrons–nuclei quantum dynamics computation. *ACS Omega* **7**, 19784–19793 (2022).
18. J. R. McClean, N. C. Rubin, J. Lee, M. P. Harrigan, T. E. O'Brien, R. Babbush, W. J. Huggins, H.-Y. Huang, What the foundations of quantum computer science teach us about chemistry. *J. Chem. Phys.* **155**, 150901 (2021).
19. C. Cerjan, *Numerical Grid Methods and Their Application to Schrödinger's Equation* (Springer Science & Business Media, 2013), vol. 412.
20. J. C. Light, T. Carrington Jr., in *Discrete-Variable Representations and their Utilization* (John Wiley and Sons Ltd, 2000), pp. 263–310.
21. C. Leforestier, R. H. Bisseling, C. Cerjan, M. D. Feit, R. Friesner, A. Guldberg, A. Hammerich, G. Jolicard, W. Karrlein, H.-D. Meyer, N. Lipkin, O. Roncero, R. Kosloff, A comparison of different propagation schemes for the time dependent Schrödinger equation. *J. Comput. Phys.* **94**, 59–80 (1991).

22. B. I. Schneider, L. A. Collins, S. X. Hu, Parallel solver for the time-dependent linear and nonlinear Schrödinger equation. *Phys. Rev. E* **73**, 036708 (2006).
23. R. J. Harrison, G. Beylkin, F. A. Bischoff, J. A. Calvin, G. I. Fann, J. Fosso-Tande, D. Galindo, J. R. Hammond, R. Hartman-Baker, J. C. Hill, J. Jia, J. S. Kottmann, M.-J. Yvonne Ou, J. Pei, L. E. Ratcliff, M. G. Reuter, A. C. Richie-Halford, N. A. Romero, H. Sekino, W. A. Shelton, B. E. Sundahl, W. S. Thornton, E. F. Valeev, Á. Vázquez-Mayagoitia, N. Vence, T. Yanai, Y. Yokoi, MADNESS: A multiresolution, adaptive numerical environment for scientific simulation. *SIAM J. Sci. Comput.* **38**, S123–S142 (2016).
24. R. Babbush, D. W. Berry, J. R. McClean, H. Neven, Quantum simulation of chemistry with sublinear scaling in basis size. *npj Quantum Inf.* **5**, 92 (2019).
25. R. E. Fenna, B. W. Matthews, Chlorophyll arrangement in a bacteriochlorophyll protein from *Chlorobium limicola*. *Nature* **258**, 573–577 (1975).
26. B. Kohler, J. L. Krause, F. Raksi, K. R. Wilson, V. V. Yakovlev, R. M. Whitnell, Y. Yan, Controlling the future of matter. *Acc. Chem. Res.* **28**, 133–140 (1995).
27. C. Sparrow, E. Martín-López, N. Maraviglia, A. Neville, C. Harrold, J. Carolan, Y. N. Joglekar, T. Hashimoto, N. Matsuda, J. L. O’Brien, D. P. Tew, A. Laing, Simulating the vibrational quantum dynamics of molecules using photonics. *Nature* **557**, 660–667 (2018.
28. M. Rühle, M. Wilkens, in *Physical Metallurgy (Fourth Edition),* R. W. Cahn, P. Haasen, Eds. (North-Holland, ed. 4, 1996), pp. 1033–1113.
29. J. E. Hudson, C. Vallance, M. Bart, P. W. Harland, Absolute electron-impact ionization cross sections for a range of C_1 to C_5 chlorocarbons. *J. Phys. B At. Mol. Opt. Phys.* **34**, 3025–3039 (2001).
30. J. A. Fleck Jr., J. Morris, M. Feit, Time-dependent propagation of high energy laser beams through the atmosphere. *Appl. Phys.* **10**, 129–160 (1976).
31. N. J. Ward, I. Kassal, A. Aspuru-Guzik, Preparation of many-body states for quantum simulation. *J. Chem. Phys.* **130**, 194105 (2009).
32. T. Jones, A. Brown, I. Bush, S. C. Benjamin, QuEST and high performance simulation of quantum computers. *Sci. Rep.* **9**, 10736 (2019).
33. T. Jones, S. C. Benjamin, QuESTlink—Mathematica embiggened by a hardware-optimised quantum emulator. *Quantum Sci. Technol.* **5**, 034012 (2020).
34. R. Meister, *pyQuEST - A Python interface for the Quantum Exact Simulation Toolkit (QuEST)* (2022); https://github.com/rrmeister/pyQuEST.

35. D. G. W. Parfitt, M. E. Portnoi, The two-dimensional hydrogen atom revisited. *J. Math. Phys.* **43**, 4681–4691 (2002).
36. X. L. Yang, S. H. Guo, F. T. Chan, K. W. Wong, W. Y. Ching, Analytic solution of a two-dimensional hydrogen atom. I. Nonrelativistic theory. *Phys. Rev. A* **43**, 1186–1196 (1991).
37. A. Mokhtari, P. Cong, J. L. Herek, A. H. Zewail, Direct femtosecond mapping of trajectories in a chemical reaction. *Nature* **348**, 225–227 (1990).
38. R. Meister, C. Gustiani, S. C. Benjamin, Exploring ab initio machine synthesis of quantum circuits. arXiv:2206.11245 [quant-ph] (22 June 2022).
39. C. Gidney, N. C. Jones, A CCCZ gate performed with 6 T gates. arXiv:2106.11513 [quant-ph] (22 June 2021).
40. A. Bhattacharyya, On a measure of divergence between two statistical populations defined by their probability distributions. *Bull. Calcutta Math. Soc.* **35**, 99–109 (1943).
41. D. W. Berry, M. Kieferová, A. Scherer, Y. R. Sanders, G. H. Low, N. Wiebe, C. Gidney, R. Babbush, Improved techniques for preparing eigenstates of fermionic Hamiltonians. *npj Quantum Inf.* **4**, 22 (2018).
42. Wikipedia page 'Atomic radii of the elements (data page)' collates multiple data sets; https://pnas.org/content/early/2008/11/24/0808245105.
43. M. Beverland, E. Campbell, M. Howard, V. Kliuchnikov, Lower bounds on the non-Clifford resources for quantum computations. *Quantum Sci. Technol.* **5**, 035009 (2020).
44. C. Gidney, A. G. Fowler, Flexible layout of surface code computations using AutoCCZ states. arXiv:1905.08916 [quant-ph] (2019).
45. D. Litinski, A game of surface codes: Large-scale quantum computing with lattice surgery. *Quantum* **3**, 128 (2019).
46. A. G. Fowler, M. Mariantoni, J. M. Martinis, A. N. Cleland, Surface codes: Towards practical large-scale quantum computation. *Phys. Rev. A* **86**, 032324 (2012).
47. B. Koczor, Exponential error suppression for near-term quantum devices. *Phys. Rev. X* **11**, 031057 (2021).
48. W. J. Huggins, S. McArdle, T. E. O'Brien, J. Lee, N. C. Rubin, S. Boixo, K. B. Whaley, R. Babbush, J. R. McClean, Virtual distillation for quantum error mitigation. *Phys. Rev. X* **11**, 041036 (2021).
49. H. Jnane, B. Undseth, Z. Cai, S. C. Benjamin, B. Koczor, Multicore quantum computing. arXiv:2201.08861 [quant-ph] (21 January 2022).

50. R. Nigmatullin, C. J. Ballance, N. de Beaudrap, S. C. Benjamin, Minimally complex ion traps as modules for quantum communication and computing. *New J. Phys.* **18**, 103028 (2016).
51. N. Vence, R. Harrison, P. Krstić, Attosecond electron dynamics: A multiresolution approach. *Phys. Rev. A* **85**, 033403 (2012).
52. H. Bauke, C. H. Keitel, Accelerating the Fourier split operator method via graphics processing units. *Comput. Phys. Commun.* **182**, 2454–2463 (2011).
53. M. Ruf, H. Bauke, C. H. Keitel, A real space split operator method for the Klein–Gordon equation. *J. Comput. Phys.* **228**, 9092–9106 (2009).
54. N. Stamatopoulos, D. J. Egger, Y. Sun, C. Zoufal, R. Iten, N. Shen, S. Woerner, Option pricing using quantum computers. *Quantum* **4**, 291 (2020).
55. S. Chakrabarti, R. Krishnakumar, G. Mazzola, N. Stamatopoulos, S. Woerner, W. J. Zeng, A threshold for quantum advantage in derivative pricing. *Quantum* **5**, 463 (2021).
56. F. Black, M. Scholes, The pricing of options and corporate liabilities. *J. Polit. Econ.* **81**, 637–654 (1973).
57. R. C. Merton, Theory of rational option pricing. *Bell J. Econ. Manage. Sci.* **4**, 141–183 (1973).
58. M. Feit, J. Fleck, A. Steiger, Solution of the Schrödinger equation by a spectral method. *J. Comput. Phys.* **47**, 412–433 (1982).
59. M. D. Feit, J. A. Fleck, Solution of the Schrödinger equation by a spectral method II: Vibrational energy levels of triatomic molecules. *J. Chem. Phys.* **78**, 301–308 (1983).
60. R. Kosloff, D. Kosloff, Absorbing boundaries for wave propagation problems. *J. Comput. Phys.* **63**, 363–376 (1986).
61. S. Choi, J. Vaníček, Efficient geometric integrators for nonadiabatic quantum dynamics. I. The adiabatic representation. *J. Chem. Phys.* **150**, 204112 (2019).
62. J. Roulet, S. Choi, J. Vaníček, Efficient geometric integrators for nonadiabatic quantum dynamics. II. The diabatic representation. *J. Chem. Phys.* **150**, 204113 (2019).
63. R. Kosloff, Time-dependent quantum-mechanical methods for molecular dynamics. *J. Phys. Chem.* **92**, 2087–2100 (1988).
64. M. Suzuki, General theory of fractal path integrals with applications to many-body theories and statistical physics. *J. Math. Phys.* **32**, 400–407 (1991).
65. A. M. Childs, Y. Su, M. C. Tran, N. Wiebe, S. Zhu, Theory of Trotter error with commutator scaling. *Phys. Rev. X* **11**, 011020 (2021).

66. T. Häner, M. Roetteler, K. M. Svore, Optimizing Quantum circuits for arithmetic. arXiv:1805.12445 [quant-ph] (31 May 2018).
67. A. Y. Kitaev, Quantum measurements and the Abelian Stabilizer Problem. arXiv:quant-ph/9511026 (20 November 1995).
68. M. Dobšíček, G. Johansson, V. Shumeiko, G. Wendin, Arbitrary accuracy iterative quantum phase estimation algorithm using a single ancillary qubit: A two-qubit benchmark. *Phys. Rev. A* **76**, 030306 (2007).
69. D. S. Abrams, S. Lloyd, Simulation of many-body fermi systems on a universal quantum computer. *Phys. Rev. Lett.* **79**, 2586–2589 (1997).
70. A. Szabo, N. Ostlund, *Modern Quantum Chemistry: Introduction to Advanced Electronic Structure Theory* (Dover Publications, 1996).
71. D. Poulin, P. Wocjan, Preparing ground states of quantum many-body systems on a quantum computer. *Phys. Rev. Lett.* **102**, 130503 (2009).
72. T. Liu, J.-G. Liu, H. Fan, Probabilistic nonunitary gate in imaginary time evolution. *Quantum Inf. Process.* **20**, 204 (2021).
73. U. V. Riss, H. D. Meyer, Reflection-free complex absorbing potentials. *J. Phys. B At. Mol. Opt. Phys.* **28**, 1475–1493 (1995).
74. G. Jolicard, E. J. Austin, Optical potential stabilisation method for predicting resonance levels. *Chem. Phys. Lett.* **121**, 106–110 (1985).
75. D. Neuhauser, M. Baer, The time-dependent Schrödinger equation: Application of absorbing boundary conditions. *J. Chem. Phys.* **90**, 4351–4355 (1989).
76. U. V. Riss, H. D. Meyer, Calculation of resonance energies and widths using the complex absorbing potential method. *J. Phys. B At. Mol. Opt.* **26**, 4503–4535 (1993).
77. G. G. Balint-Kurti, Á. Vibók, in *Complex Absorbing Potentials in Time Dependent Quantum Dynamics*, C. Cerjan, Ed. (Springer Netherlands, 1993), pp. 195–205.
78. S. Scheit, H.-D. Meyer, N. Moiseyev, L. S. Cederbaum, On the unphysical impact of complex absorbing potentials on the Hamiltonian and its remedy. *J. Chem. Phys.* **124**, 034102 (2006).
79. F. Gatti, B. Lasorne, H.-D. Meyer, A. Nauts, in *Introduction to Numerical Methods* (Springer International Publishing, 2017), pp. 201–285.
80. A. Richards, *University of Oxford Advanced Research Computing* (2015); https://zenodo.org/record/22558.

81. R. Kosloff, Propagation methods for quantum molecular dynamics. *Annu. Rev. Phys. Chem.* **45**, 145–178 (1994).
82. D. Tannor, *Introduction to Quantum Mechanics* (University Science Books, 2007).
83. R. Sadeghi, R. T. Skodje, Spectral quantization of high energy transition state resonances in the H + H2 reaction. *J. Chem. Phys.* **99**, 5126–5140 (1993).
84. D. H. Zhang, J. Z. H. Zhang, Quantum calculations of reaction probabilities for HO + CO→ H + CO_2 and bound states of HOCO. *J. Chem. Phys.* **103**, 6512–6519 (1995).
85. V. N. Serov, V. B. Sovkov, V. S. Ivanov, O. Atabek, Split operator method for the nonadiabatic (J=0) bound states and (A←X) absorption spectrum of NO_2. *J. Chem. Phys.* **115**, 6450–6458 (2001).
86. X. Chen, V. S. Batista, Matching-pursuit/split-operator-Fourier-transform simulations of excited-state nonadiabatic quantum dynamics in pyrazine. *J. Chem. Phys.* **125**, 124313 (2006).
87. A. D. Bandrauk, H. Shen, Exponential split operator methods for solving coupled time-dependent Schrödinger equations. *J. Chem. Phys.* **99**, 1185–1193 (1993).
88. S. M. Greene, V. S. Batista, Tensor-Train Split-Operator Fourier Transform (TT-SOFT) method: Multidimensional nonadiabatic quantum dynamics. *J. Chem. Theory Comput.* **13**, 4034–4042 (2017).
89. B. F. E. Curchod, T. J. Martínez, Ab initio nonadiabatic quantum molecular dynamics. *Chem. Rev.* **118**, 3305–3336 (2018).
90. Y. Wu, V. S. Batista, Matching-pursuit for simulations of quantum processes. *J. Chem. Phys.* **118**, 6720–6724 (2003).
91. Y. Wu, V. S. Batista, Quantum tunneling dynamics in multidimensional systems: A matching-pursuit description. *J. Chem. Phys.* **121**, 1676–1680 (2004).
92. V. A. Trofimov, N. Peskov, Comparison of finite-difference schemes for the Gross-Pitaevskii equation. *Math. Model. Anal.* **14**, 109–126 (2009).
93. C. Dion, A. Hashemloo, G. Rahali, Program for quantum wave-packet dynamics with time-dependent potentials. *Comput. Phys. Commun.* **185**, 407–414 (2014).
94. B. Buonacorsi, B. Shaw, J. Baugh, Simulated coherent electron shuttling in silicon quantum dots. *Phys. Rev. B* **102**, 125406 (2020).
95. P. L. DeVries, Application of the split operator Fourier transform method to the solution of the nonlinear Schrödinger equation. *AIP Conf. Proc.* **160**, 269–271 (1987).
96. J. A. C. Weideman, B. M. Herbst, Split-step methods for the solution of the nonlinear Schrödinger equation. *SIAM J. Numer. Anal.* **23**, 485–507 (1986).

97. E. Faou, L. Gauckler, C. Lubich, Plane wave stability of the split-step Fourier method for the nonlinear Schrödinger equation. *Forum Math. Sigma* **2**, e5 (2014).
98. R. Kosloff, in *The Fourier Method*, C. Cerjan, Ed. (Springer Netherlands, 1993), pp. 175–194.
99. R. Babbush, N. Wiebe, J. McClean, J. McClain, H. Neven, G. K.-L. Chan, Low-depth quantum simulation of materials. *Phys. Rev. X* **8**, 011044 (2018).
100. J. P. Boyd, *Chebyshev and Fourier Spectral Methods: Second Revised Edition* (Dover Publications Inc., 2001).
101. H. Buhrman, R. Cleve, J. Watrous, R. de Wolf, Quantum fingerprinting. *Phys. Rev. Lett.* **87**, 167902 (2001).
102. Y. R. Sanders, D. W. Berry, P. C. S. Costa, L. W. Tessler, N. Wiebe, C. Gidney, H. Neven, R. Babbush, Compilation of fault-tolerant quantum heuristics for combinatorial optimization. *PRX Quantum* **1**, (2020).
103. B. Poirier, Efficient evaluation of exponential and Gaussian functions on a quantum computer. arXiv:2110.05653 [quant-ph] (2021).
104. T. G. Draper, Addition on a quantum computer. arXiv:quant-ph/0008033 (2000).
105. Wolfram Research Incorporated, *Wolfram Engine* (2021); https://wolfram.com/engine/.
106. L. Grover, T. Rudolph, Creating superpositions that correspond to efficiently integrable probability distributions. arXiv:quant-ph/0208112 (2002).
107. P. Kaye, M. Mosca, Quantum networks for generating arbitrary quantum states, in *Optical Fiber Communication Conference and International Conference on Quantum Information, 2001 OSA Technical Digest Series* (Optica Publishing Group, 2001), paper PB28.
108. M. Mottonen, J. J. Vartiainen, V. Bergholm, M. M. Salomaa, Transformation of quantum states using uniformly controlled rotation. *Quantum Info. Comput.* **5**, 467–473 (2005).
109. G. Marin-Sanchez, J. Gonzalez-Conde, M. Sanz, Quantum algorithms for approximate function loading. arXiv:2111.07933 [quant-ph] (2021).
110. A. Kitaev, W. A. Webb, Wavefunction preparation and resampling using a quantum computer. arXiv:0801.0342 [quant-ph] (2009).
111. A. Holmes, A. Y. Matsuura, in *2020 IEEE International Conference on Quantum Computing and Engineering (QCE)* (Los Alamitos, CA, USA, 2020), pp. 169–179.
112. A. Carrera Vazquez, S. Woerner, Efficient state preparation for quantum amplitude estimation. *Phys. Rev. Appl.* **15**, 034027 (2021).

113. A. G. Rattew, Y. Sun, P. Minssen, M. Pistoia, The efficient preparation of normal distributions in quantum registers. *Quantum* **5**, 609 (2021).
114. A. G. Rattew, B. Koczor, Preparing arbitrary continuous functions in quantum registers with logarithmic complexity. arXiv:2205.00519 [quant-ph] (2022).
115. E. Farhi, J. Goldstone, S. Gutmann, M. Sipser, Quantum computation by adiabatic evolution. arXiv:quant-ph/0001106 (2000).
116. L. Lin, Y. Tong, Near-optimal ground state preparation. *Quantum* **4**, 372 (2020).
117. Y. Dong, L. Lin, Y. Tong, Ground state preparation and energy estimation on early fault-tolerant quantum computers via quantum eigenvalue transformation of unitary matrices. *PRX Quantum* **3**, 040305 (2022).
118. F. M. Fernandez, Analytical bound eigenstates and eigenvalues of a truncated Coulomb potential. *J. Phys. A Math. Gen.* **24**, 1351–1353 (1991).
119. R. L. Hall, N. Saad, K. D. Sen, H. Ciftci, Energies and wave functions for a soft-core Coulomb potential. *Phys. Rev. A* **80**, 032507 (2009).
120. C. Li, Exact analytical solution of the ground-state hydrogenic problem with soft Coulomb potential. *J. Phys. Chem. A.* **125**, 5146–5151 (2021).
121. J. Spencer, A. Alavi, Efficient calculation of the exact exchange energy in periodic systems using a truncated Coulomb potential. *Phys. Rev. B* **77**, 193110 (2008).
122. F. J. Rogers, H. C. Graboske, D. J. Harwood, Bound eigenstates of the static screened Coulomb potential. *Phys. Rev. A* **1**, 1577–1586 (1970).
123. H. J. Silverstone, Long-range behavior of electronic wave functions. generalized Carlson-Keller expansion. *Phys. Rev. A* **23**, 1030–1037 (1981).
124. D. C. Ghosh, R. Biswas, Theoretical calculation of absolute radii of atoms and ions. Part 1. The atomic radii. *Int. J. Mol. Sci.* **3**, 87–113 (2002).
125. R. D. J. III, NIST Computational Chemistry Comparison and Benchmark Database, in *NIST Standard Reference Database Number 101 Release 22* (2022).
